# Multi-stimuli Control over Assembly and Guest Binding in Metallo-supramolecular Hosts Based on Dithienylethene Photoswitches

**DOI:** 10.1021/jacs.0c12188

**Published:** 2021-03-05

**Authors:** Ru-Jin Li, Jacopo Tessarolo, Haeri Lee, Guido H. Clever

**Affiliations:** Faculty of Chemistry & Chemical Biology, TU Dortmund University, Otto-Hahn-Straße 6, 44227 Dortmund, Germany

## Abstract

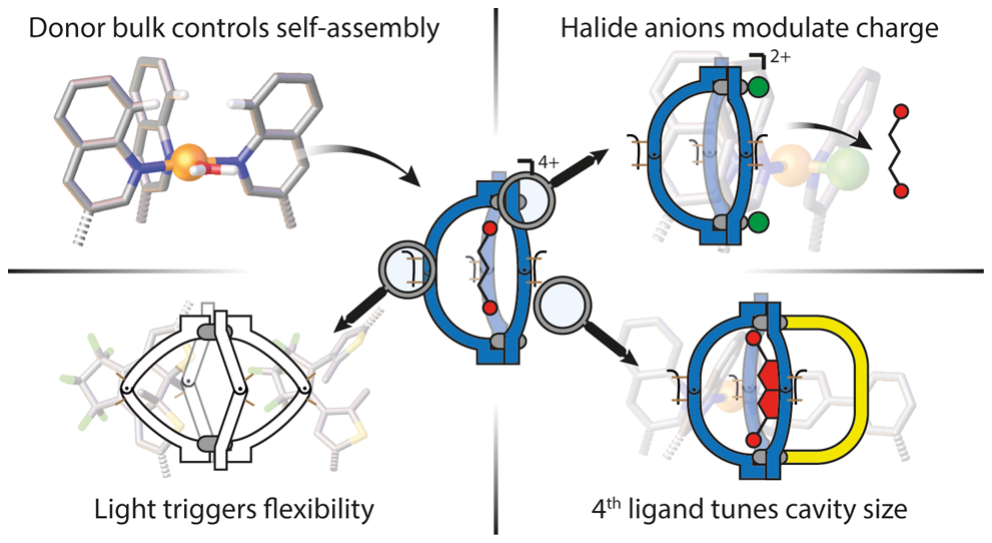

It is difficult to assemble multi-component metallo-supramolecular architectures in a non-statistical fashion, which limits their development toward functional materials. Herein, we report a system of interconverting bowls and cages that are able to respond to various selective stimuli (light, ligands, anions), based on the self-assembly of a photochromic dithienylethene (DTE) ligand, **L**^**a**^, with Pd^II^ cations. By combining the concept of “coordination sphere engineering”, relying on bulky quinoline donors, with reversible photoswitching between the ligand’s open (*o*-**L**^**a**^) and closed (*c*-**L**^**a**^) forms, a [Pd_2_(*o*-**L**^**a**^)_4_] cage (*o*-**C**) and a [Pd_2_(*c*-**L**^**a**^)_3_] bowl (*c*-**B**) were obtained, respectively. This structural rearrangement modulates the system’s guest uptake capabilities. Among three bis-sulfonate guests (**G1**, **G2**, and **G3**), the cage can encapsulate only the smallest (**G1**), while the bowl binds all of them. Bowl *c*-**B** was further used to synthesize a series of heteroleptic cages, [Pd_2_L^A^_3_L^B^], representing a motif never reported before. Additional ligands (**L**^**c-f**^), with short or long arms, tune the cavity size, thus enabling or preventing guest uptake. Addition of Br^–^/Ag^+^ makes it possible to change the overall charge, again triggering guest uptake and release, as well as fourth ligand de-/recomplexation. In combination, site-selective introduction of functionality and application of external stimuli lead to an intricate system of hosts with different guest preferences. A high degree of complexity is achieved through cooperativity between only a few components.

## Introduction

Natural systems are capable of fabricating supramolecular assemblies with multiple components in a precise and controlled manner. For example, ribosomes and virus capsids are assembled in a non-covalent fashion, starting from smaller building blocks. To mimic the function and complexity of natural nanostructures, chemists have developed supramolecular self-assembly, a field that has become a burgeoning area of study.^1^ In particular, the design of coordination-driven architectures represents a powerful strategy. Owing to the directionality and reversibility of the metal–ligand bond, it is possible to create a plethora of architectures with different sizes, shapes, and functions.^2^ In the past years, metallacages have received great attention due to their peculiar structures^3^ and promising functions,^4^ e.g., in selective recognition^5^ and catalysis.^6^ However, while most natural assemblies are complex multi-functional systems comprising several different building blocks, most artificial metallo-supramolecular assemblies known today are highly symmetric structures, formed from only one type of ligand. Increasing such a system’s complexity via the introduction of multiple different ligands is challenging, since metal-mediated self-assembly under thermodynamic control easily results in a statistical mixture of products.^7^ In recent years, several strategies to combine distinguishable ligands in a non-statistical fashion, leading to the clean assembly of heteroleptic cages, have been reported. For example, the Yoshizawa group explored the use of a templating guest,^8^ Crowley et al. took advantage of hydrogen-bonding between adjacent ligands,^9^ and Zhou,^10a^ Fujita,^10b^ Mukherjee,^10c,10d^ Chand,^10e,10f^ and our group^10g−10i^ utilized geometric principles to form heteroleptic cages containing two different types of ligands. Recently, our group set out to investigate a “coordination sphere engineering” approach based on the introduction of steric hindrance around the metal centers to increase the structural complexity in a series of Pd^II^-mediated assemblies. On one hand, banana-shaped ditopic ligands with picoline donors allowed us to form *cis*-configured [Pd_2_L^A^_2_L^B^_2_]^4+^ cages,^11^ while the introduction of quinoline or acridine donor groups resulted in the assembly of [Pd_2_L_3_X_2_] bowl-shaped structures or [Pd_2_L_2_X_4_] rings, respectively (X = solvent or halide anion).^12^ Despite these few examples, rational multi-component assembly to give a single product remains challenging.^13^ Even more so, exploiting the multi-ligand character of heteroleptic cages to introduce functional complexity is still largely unexplored, in particular with respect to emergent properties resulting from an interplay of different components. Owing to their dynamic behavior and the presence of an accessible cavity, coordination cages with stimuli-responsive functionality represent a promising platform to develop novel materials^14^ and adaptive host–guest systems.^15^

It has been shown that interaction of such cages with chemical stimuli, e.g., additional ligands, allows structural rearrangement and guest release.^16^ The addition of halide anions to positively charged hosts can trigger the uptake of neutral guest molecules.^17^ Guest uptake can also be tuned in redox-responsive systems.^18^ Sophisticated pH effects have recently been investigated^19^ and combined with light, leading to reversible cage disassembly.^20^ Light as a waste-free, precisely administered external stimulus has gained high popularity in the control of supramolecular systems.^14a,21^ We previously reported light-responsive cages in which photochromic dithienylethene (DTE) ligands made it possible to control structural rearrangements as well as guest uptake and release in a fully reversible fashion.^22^ Most reported examples of stimuli-responsive, metallo-supramolecular systems are based on homoleptic structures, though.^23^ Hence, the introduction of more than one functionality or response to more than one stimulus is a challenge.

**Figure 1:**
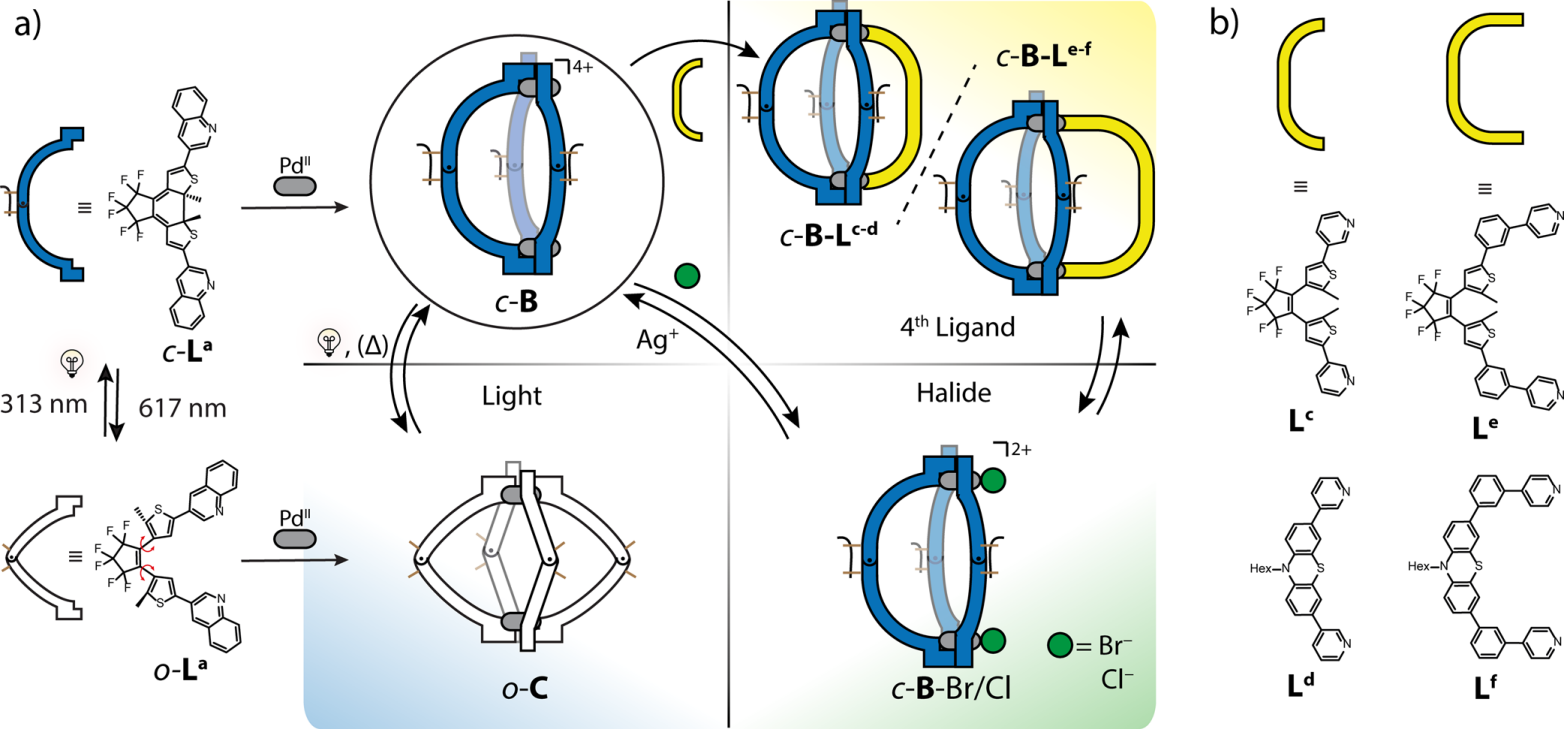
(a) Self-assembly of **L**^**a**^ with Pd^II^ to form a [Pd_2_**L**^**a**^_4_] cage (*o*-**C**) or a [Pd_2_**L**^**a**^_3_] bowl (*c-***B**) that reacts to multiple stimuli: light triggers reversible structural rearrangement between *c-***B** and *o*-**C** (blue panel), halides substitute solvents to saturate the Pd^II^ coordination environments while reducing the overall charge from 4+ to 2+ (green panel), and addition of pyridine-based fourth ligands leads to [Pd_2_L^A^_3_L^B^] heteroleptic cages (*c-***B**-**L**^**c-f**^) (yellow panel). (b) List of short- (**L**^**c,d**^) and long-arm (**L**^**e,f**^) bis-pyridyl ligands.

We herein demonstrate that multi-stimuli-responsive systems can be realized by combining three well-established trigger elements via non-statistical, heteroleptic self-sorting. We combine a strategy we coined “coordination sphere engineering” in tandem with light-responsive DTE backbones to achieve the self-assembly of an interconvertible pair of [Pd_2_L^A^_4_] cage- and [Pd_2_L^A^_3_] bowl-shaped hosts, the former deriving from the open-form photoisomer (*o*-**L**^**a**^) and the latter from the closed isomer (*c*-**L**^**a**^; Figure 1a). The latter system then serves as a platform for the non-statistical formation of a series of heteroleptic cages [Pd_2_L^A^_3_L^B^] with tunable cavity size and guest binding affinity (Figure 1a, yellow panel). Besides its susceptibility to light, the system shows stimuli-responsive behavior triggered by chemical species (fourth ligands L^B^ and halide anions), making it possible to differentiate a series of anionic guests and control their uptake and release.

## Results and Discussion

### Self-Assembly of Homoleptic Cages and Bowls

**Figure 2:**
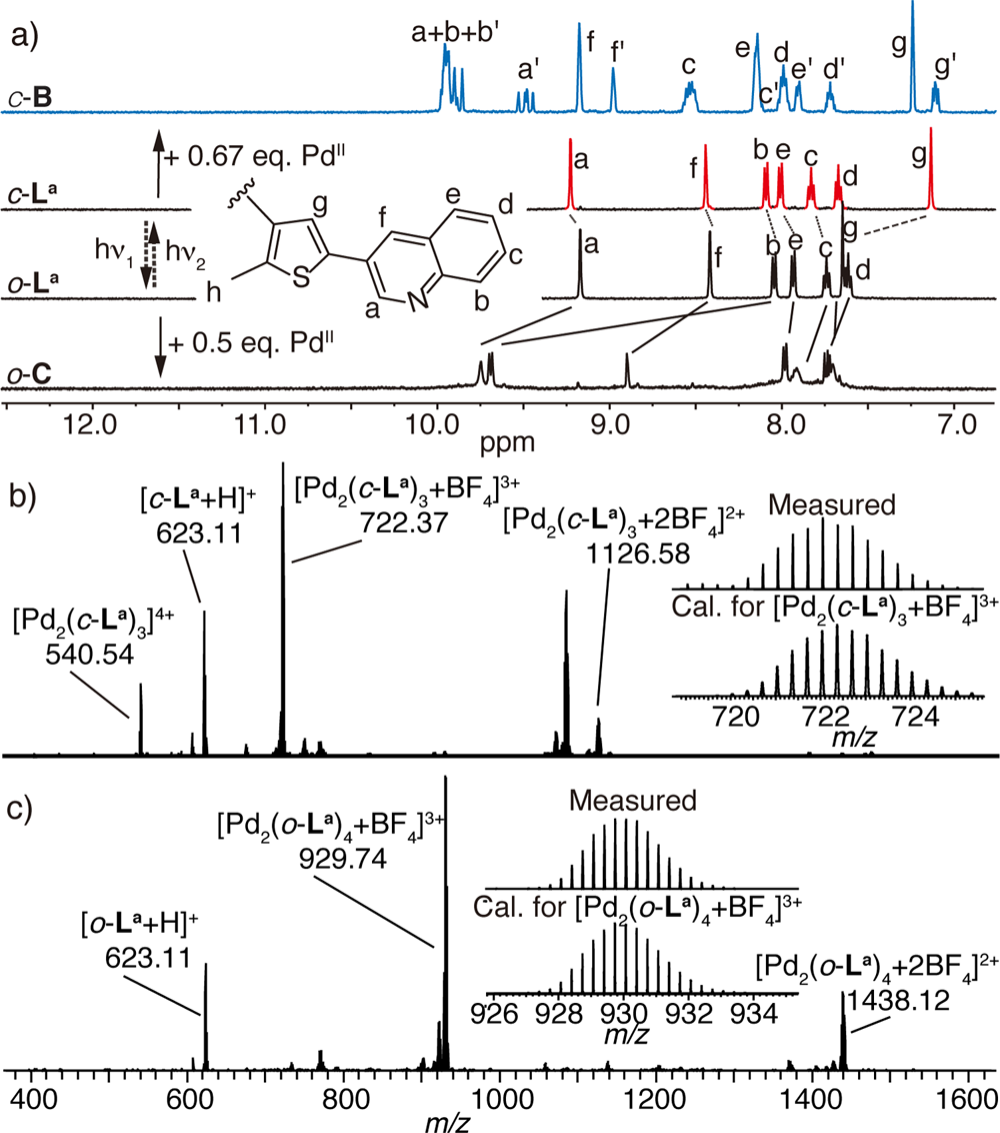
(a) ^1^H NMR spectra (500 MHz, CD_3_CN, 298 K) of ligands *o*-**L**^**a**^, *c*-**L**^**a**^, *o*-**C**, and *c*-**B**. (b, c) ESI-MS spectra of (b) *c*-**B** with isotope pattern of [*c*-**B**+BF_4_]^3+^ shown in the inset and (c) *o*-**C** with isotope pattern of [*o*-**C**+BF_4_]^3+^.

**Figure 3:**
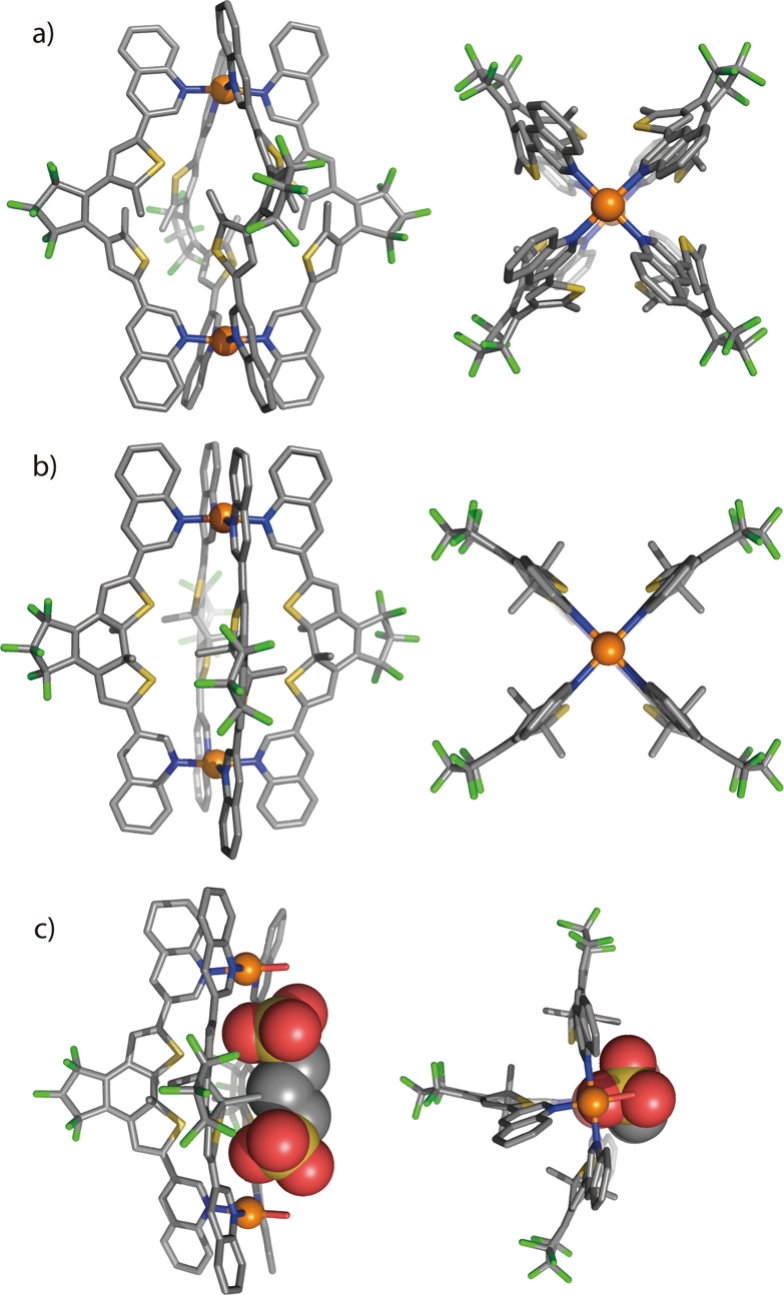
X-ray structures of (a) *o*-**C**, (b) *c*-**C**, and (c) host–guest complex **G1**@*c*-**B** (fourth Pd^II^ positions coordinated by H_2_O) in side (left) and top (right) views. All hydrogen atoms, solvent molecules, counteranions, and minor disorders are omitted for clarity. In *c*-**C** and **G1**@*c*-**B**, all backbone methyl groups are disordered over both possible positions with approximately 50% occupancy (here only one diastereomer is shown; C gray, N blue, F green, S yellow, O red, Pd orange).

The general design of the system is based on previous work from our group on photochromic [Pd_2_L_2*n*_] assemblies formed from banana-shaped bis-pyridine ligands having DTE backbones.^14a,21^ In this work, we combine the effect of the photochromic backbones with coordination sphere engineering by installing quinolin-3-yl donor groups that introduce significant steric hindrance around the coordinated Pd^II^ cations. We had shown previously that, in such quinoline-based assemblies, a fine balance separates the formation of sterically less crowded [Pd_2_L_3_] bowl-shaped structures and more constrained [Pd_2_L_4_] cages.^12^ Here, we show how this balance can be tipped by photoisomerizing the ligand backbones in one direction or the other. Ligand *o*-**L**^**a**^ was synthesized by a Suzuki cross-coupling reaction of perfluoro-1,2-bis(2-iodo-5-methylthien-4-yl)cyclopentene and 2 equiv of quinolin-3-yl boronic acid. Self-assembly of *o*-**L**^**a**^, with the photoswitch in its open form, and [Pd(CH_3_CN)_4_](BF_4_)_2_ in a 2:1 ratio in CD_3_CN at 70 °C led to the quantitative formation of cage compound [Pd_2_(*o*-**L**^**a**^)_4_](BF_4_)_4_ (*o*-**C**; Figure 1a, blue panel). Assembly was unambiguously confirmed by NMR spectroscopy and high-resolution electrospray ionization mass spectrometry (HR-ESI-MS) (Figure 2a,c) as well as single-crystal X-ray structure determination (Figure 3a). Slow vapor diffusion of Et_2_O into a CD_3_CN solution of *o*-**C** allowed us to grow single crystals suitable for synchrotron diffraction analysis. The compound crystallized in the tetragonal space group *P*4_2_, and the structure was confirmed to be a [Pd_2_L_4_] cage. Compared with our reported [Pd_2_L_4_] DTE-based cage derivatives,^21^ in *o*-**C**, the *C*_2_-symmetric ligands are all slightly helically twisted along the Pd–Pd axis, presumably helping to release steric hindrance around the two coordination centers. This becomes clear by measuring the angles between the normals to the plane created by Pd and the four coordinating N atoms and the quinoline plane, having an averaged value of about 74°, as compared to 88° for our previously reported pyridyl-DTE cage (further details in the Supporting Information, Figure S121). Cage *o*-**C** features a pair of *PPPP/MMMM* enantiomers in the crystal structure (Figure S52).^24^ Irradiation of a CD_3_CN solution of *o*-**L**^**a**^ using 313 nm light allows conversion into *c*-**L**^**a**^ with >99% yield. Reacting *c-***L**^**a**^, featuring the photoswitch in its closed form, with [Pd(CH_3_CN)_4_](BF_4_)_2_ in a 3:2 ratio at 70 °C led to the formation of the bowl-shaped structure [Pd_2_(*c*-**L**^**a**^)_3_(Solvent)_2_](BF_4_)_4_ (*c*-**B**) (Figure 1a). The first result corroborating the bowl structure came from NMR analysis, since the ^1^H spectrum clearly shows two sets of signals in a 2:1 ratio (Figure 2a, Figure S13).

Due to symmetry reasons, one set of signals is expected to derive from the middle ligand, while the second set can be assigned to the two ligands flanking the side of the bowl structure.^12a^ Moreover, ^1^H DOSY NMR (298 K) further confirmed the presence of a single species in solution (Figure S17). As configurationally stable ligand *c*-**L**^**a**^ was used in its racemic form, we would expect bowl *c*-**B** to exist in six diastereomeric forms, producing altogether four sets of ^1^H NMR signals, each representing a unique chemical environment (see details in Supporting Information, Figure S52). Indeed, a 4-fold signal splitting was observed for protons H_a′_ and H_g′_ (Figure 2a, Figure S55). When the structure was assembled from enantiopure *R,R*-*c*-**L**^**a**^ or *S,S*-*c*-**L**^**a**^ (resolved by chiral HPLC), however, much clearer and sharper 1D and 2D ^1^H NMR spectra were obtained (Figures S55–S57). Further confirmation of the bowl structure arose from HR-ESI-MS, in which a series of prominent peaks assigned to the bowl with a variable number of BF_4_^–^ counterions were clearly detected ([Pd_2_(*c*-**L**^**a**^)_3_]^4+^, [Pd_2_(*c*-**L**^**a**^)_3_+BF_4_]^3+^, and [Pd_2_(*c*-**L**^**a**^)_3_+2BF_4_]^2+^) (Figure 2b). Interestingly, when *c*-**L**^**a**^ and Pd^II^ cations are reacted in the correct stoichiometry to form a [Pd_2_L_4_]^2+^ cage, ^1^H NMR analysis clearly shows signals for the free ligand (besides the bowl), corresponding to one-third of the total amount. However, after addition of one-sixth more equivalent of [Pd(CH_3_CN)_4_](BF_4_)_2_, the free ligand was completely consumed and the bowl species *c*-**B** remained quantitatively (Figure S101). Diffusion of Et_2_O into a CD_3_CN solution of *c*-**B** under strict exclusion of light gave a single crystal whose X-ray diffraction analysis revealed the structure of cage [Pd_2_(*c*-**L**^**a**^)_4_](BF_4_)_4_ (*c*-**C**) rather than the expected bowl *c*-**B**. While seemingly surprising, this result agrees with previous findings from our group, where quinoline donor groups were shown to lead to [Pd_2_L_3_] bowls in solution but [Pd_2_L_4_] cages in the solid state.^12a^ Gas-phase DFT studies indicate that energies for the formal cage/bowl transformation do not depend significantly on the photoisomeric state, further corroborating a fine energetic balance between solution and solid-state behavior (Figure S60). The compound crystallizes in the *P*4_2_/*mcm* space group, and the asymmetric unit contains both possible enantiomers with 50:50 occupancy. The crystal structure further showed that all ligands *c*-**L**^**a**^ are arranged without any twisting with respect to the Pd–Pd axis (Figure 3b; further details in the Supporting Information).

### Homoleptic Cages: Photoswitching and Host–Guest Behavior

Irradiation of *o*-**C** under UV light (313 nm) led to the formation of bowl *c*-**B**, accompanied by the release of free ligand *c*-**L**^**a**^, as observed by ^1^H NMR analysis (Figure S102). Addition of Pd^II^ cations (0.17 equiv) and further heating at 70 °C for 1 h made it possible to convert the excess free ligand *c*-**L**^**a**^ into *c*-**B** quantitatively. The obtained broad ^1^H NMR spectrum indicated that *c*-**B** was formed as a mixture of diastereomers. *Vice versa*, irradiation of *c*-**B** at room temperature with 617 nm light resulted in a broad ^1^H NMR spectrum, not showing any signs of free ligand (Figure S102). HR-ESI and even cold-spray ionization (CSI) mass spectrometry (−30 °C) could not detect any peaks related to a tentative *o*-**B** species. However, a low-temperature (−35 °C) ^1^H NMR measurement resulted in much sharper signals, allowing us to identify *o*-**B** (Figure S108). Heating this *o*-**B** solution for 1 h accelerates complete reassembly to the thermodynamic product *o*-**C** (Figure S102; 0.33 equiv of *o*-**L**^**a**^ can be added to account for the change in stoichiometry). UV–vis analysis of ligand and cage samples corroborated quantitative switching fidelity in both cases, with the ligand showing faster photoisomerization as the cage, in accordance with our previous results for related systems (Figures S122–S124).^22^ Cage–bowl interconversion was studied over 10 cycles, showing very good fatigue resistance (Figure S125).

**Figure 4:**
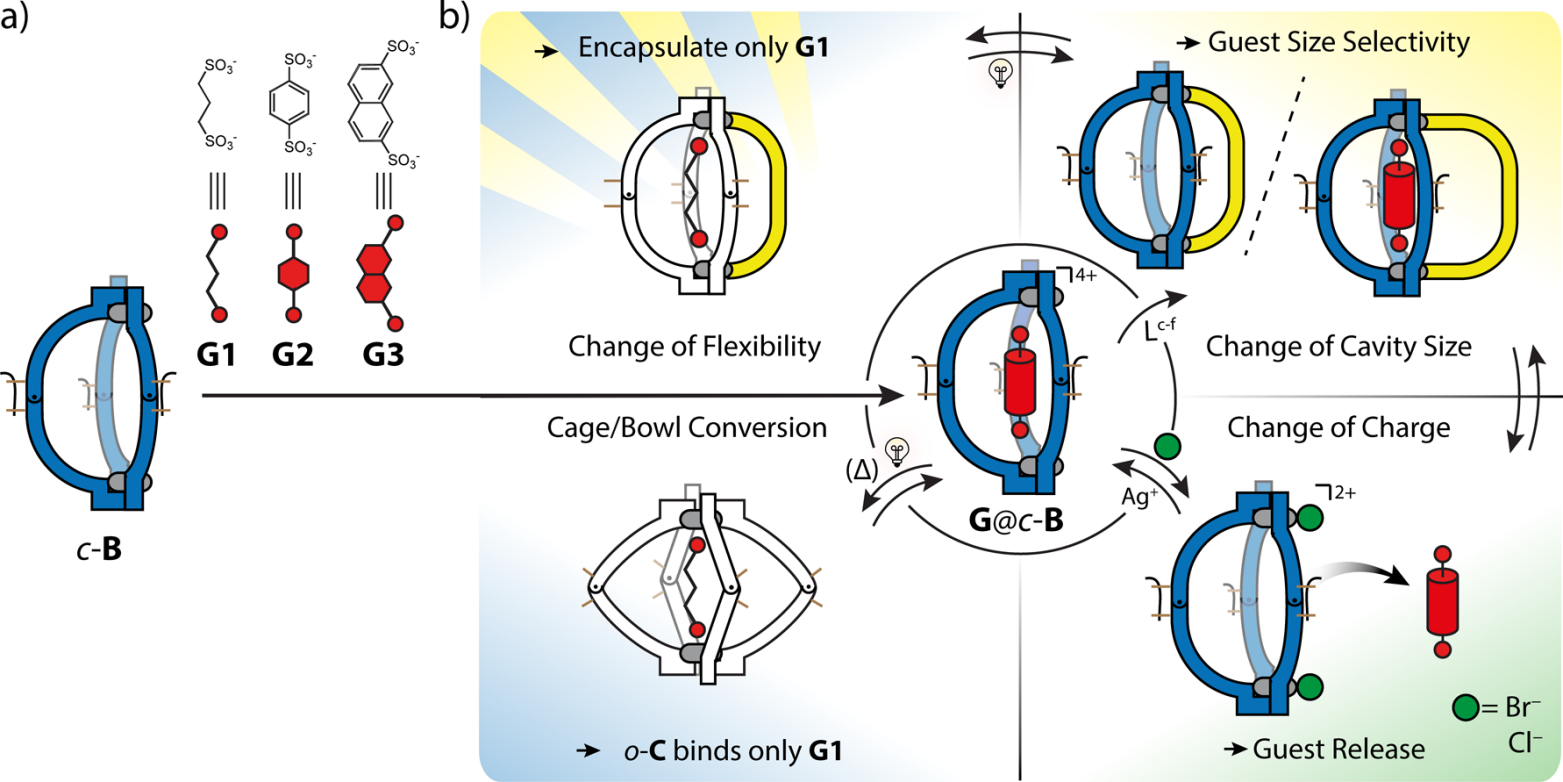
(a) *c-***B** binds a series of guests to form a **G**@*c-***B** host–guest system. (b) Stimuli response of G@c-B. Blue panel: light irradiation makes it possible to form *o-***C** that binds only **G1**. Green panel: halide coordination tunes the overall system charge, triggering guest release. Yellow panel: addition of a fourth ligand tunes the cavity size and the system’s selectivity toward **G1**, **G2**, and **G3**. Blue/yellow panel: irradiation of *c-***B**-**L**^**c-f**^ heteroleptic cages opens the photoswitches and increases the structural flexibility, modulating guest uptake selectivity and system stability.

This photo-controlled reversible interconversion between cage and bowl makes it possible to tune the size and accessibility of the inner cavity. Therefore, size-dependent guest encapsulation of *o*-**C** and *c*-**B** was investigated. To do this, we performed ^1^H NMR titrations with three different anionic bis-sulfonate guests, **G1–G3** (with NBu_4_^+^ as counterion) (Figure 4a). Stepwise addition of the smallest guest, **G1** (propane-1,3-bis-sulfonate), into a CD_3_CN solution of *o*-**C** led to the appearance of a new set of sharp ^1^H NMR signals and significant downfield shifts of the signals assigned to inward-pointing protons (H_a_, Δδ = 0.67). This indicated encapsulation and slow exchange of guest **G1** (Figure 4b, blue panel, Figure S61). The formation of host–guest complex [**G1**@*o*-**C**]^2+^ was further confirmed by HR-ESI-MS spectrometry (Figure S62). For the slightly larger guests **G2** (benzene-1,4-bis-sulfonate) and **G3** (naphthalene-2,7-bis-sulfonate), however, no encapsulation into *o*-**C** could be detected (Figures S63 and S64). On the contrary, due to its more open cavity, *c*-**B** was indeed found to be able to bind all three guest molecules, **G1**, **G2**, and **G3**, as confirmed by NMR spectroscopy and HR-ESI-MS (Figures S65–S70). To better evaluate the ^1^H NMR signal shifting, enantiopure *S*-*c*-**B** was used in the experiments. After stepwise titration with **G1**, two new signals arose around δ = 11.0 ppm, showing the formation of a new species in solution. Observation of a large peak assignable to [**G1**@*c*-**B**]^2+^ in the HR-ESI-MS spectrum further indicated binding with a 1:1 stoichiometry (Figure S66). After the addition of 3.0 equiv of the guest, a crystal suitable for single-crystal X-ray diffraction was obtained by leaving the sample overnight. Pleasingly, X-ray analysis confirmed the assumed structure of [**G1**@*c*-**B**], where guest **G1** is accommodated in the concave well of the bowl between the two Pd atoms (Figure 3c). Titration of *S-c*-**B** with **G2** and **G3** also resulted in encapsulation with a 1:1 stoichiometry, as confirmed by ^1^H NMR experiments and HR-ESI-MS spectrometry (Figures S67–S70). Unfortunately, determination of association constants for the host–guest complexes from ^1^H NMR titration data was not possible due to an onset of precipitation during the experiments. Notably, the stable interaction of **G2** with bowl-shaped c-**B** inspired us to exploit this host–guest interaction with the kinetic photoswitch product *o*-**B** at room temperature. After the addition of 1.0 equiv of **G2** into a freshly prepared solution of *o*-**B**, ^1^H NMR signals sharpened, allowing the characterization of the assembly (Figures S103–S106). HR-ESI-MS further supported the formation of host–guest complex **G2**@*o*-**B**, showing a single peak assigned to [**G2**@Pd_2_(*o*-**L**^**a**^)_3_]^2+^ (Figure S107). Moreover, **G2**@*o*-**B** and **G2**@*c*-**B** could be reversibly interconverted by irradiation with UV light (313 nm) and red light (617 nm), respectively, as verified by ^1^H NMR spectroscopy (Figure S103).

### Heteroleptic Cages: Self-Assembly, Guest Binding, and Response to Chemical Stimuli

**Figure 5:**
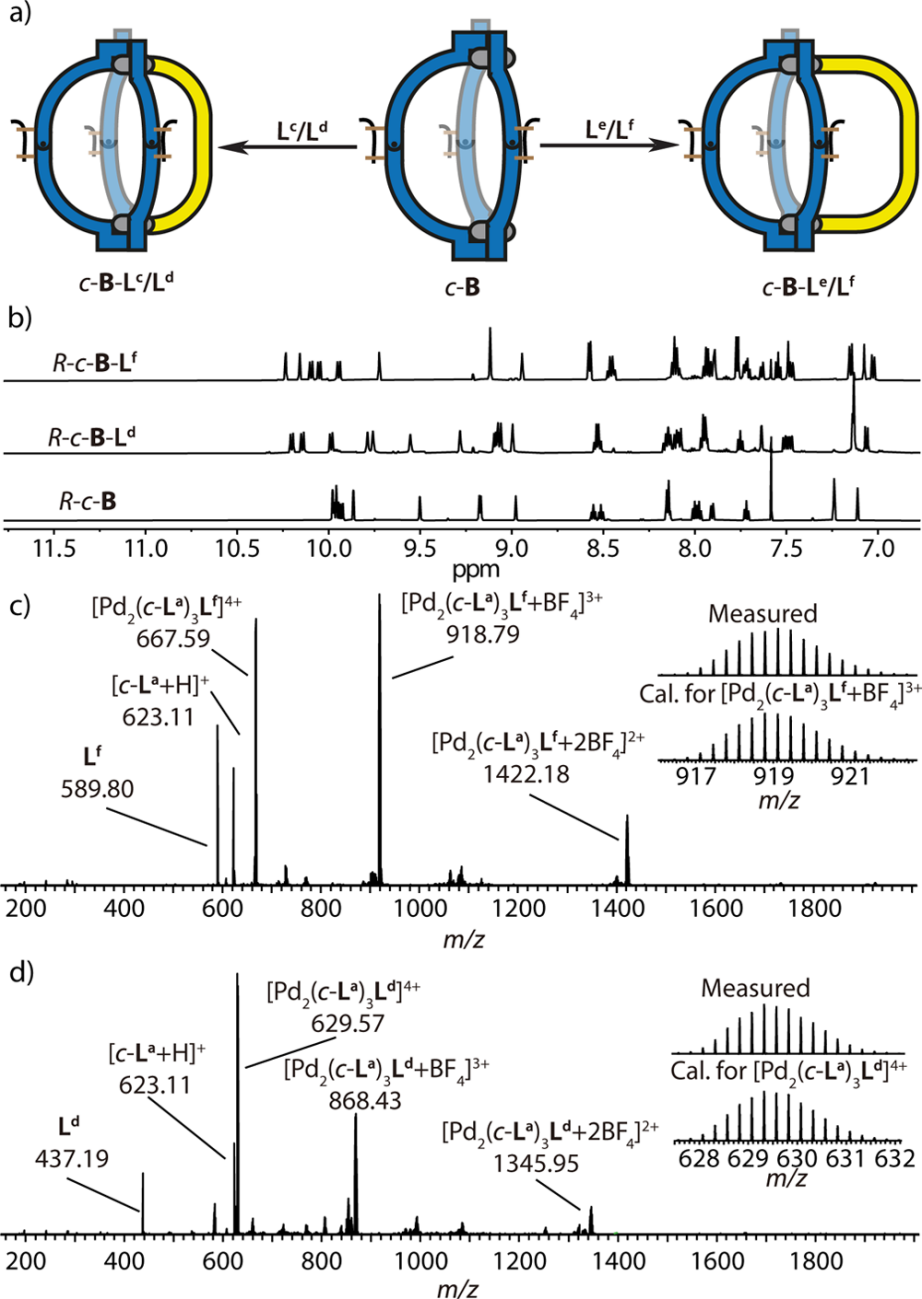
(a) Assembly of [Pd_2_L^A^_3_L^B^] heteroleptic cages: addition of short-armed ligands **L**^**c**^/**L**^**d**^ (left) or long-armed ligands **L**^**e**^/**L**^**f**^ (right) to *c*-**B** leads to the synthesis of cages *c*-**B**-**L**^**c-f**^. (b) ^1^H NMR spectra (500 MHz, CD_3_CN, 298 K) of homochiral *R*-*c*-**B**, *R*-*c*-**B**-**L**^**d**^, and *R*-*c*-**B**-**L**^**f**^. (c) ESI-MS spectrum of *c*-**B**-**L**^**f**^, with isotope pattern of [Pd_2_(*c*-**L**^**a**^)_3_**L**^**f**^ + BF_4_]^3+^ shown in the inset. (d) ESI-MS spectrum *c*-**B**-**L**^**d**^, with isotope pattern of [Pd_2_(*c*-**L**^**a**^)_3_**L**^**f**^]^4+^.

In bowl species such as *c*-**B**, each Pd^II^ center is coordinated by three quinoline-based ligands, while the fourth coordination site is occupied by a solvent molecule (here acetonitrile or H_2_O) to complement the square-planar coordination sphere.^12^ This encouraged us to synthesize a new series of banana-shaped bis-pyridine ligands (**L**^**c-f**^, Figure 1b), having a length similar to that of **L**^**a**^ (in terms of their N···N distances). While the pyridine group is a stronger donor as compared to the solvent used, it is also less sterically demanding than the quinoline donors on the other ligands, thus allowing it to replace the coordinated solvent as a fourth ligand, leading to the formation of unprecedented [Pd_2_L^A^_3_L^B^] heteroleptic cages with a 3:1 ligand stoichiometry (Figure 1a, yellow panel). Consequently, addition of 1.0 equiv of the ligands to *c*-**B** results in the formation of four heteroleptic cages with the general formula [Pd_2_(*c*-**L**^**a**^)_3_**L**^**c-f**^](BF_4_)_4_ (*c*-**B**-**L**^**c-f**^), which were fully characterized by NMR techniques and HR-ESI-MS (Figure 5, Figures S26–S51). Again, enantiopure *R/S*-*c*-**B** was used in order to obtain better resolved ^1^H NMR spectra. Taking *c*-**B**-**L**^**d**^ as an example, reaction of **L**^**d**^ with *c*-**B** resulted in the transformation of the ^1^H NMR spectrum in which neither signals of the parental bowl species nor the free ligand **L**^**d**^ are present (Figure 5b). HR-ESI-MS (Figure 5d) clearly identified peaks corresponding to [Pd_2_(*c*-**L**^**a**^)_3_**L**^**d**^ + *n*BF_4_]^(4–*n*)+^ (*n* = 0–2). Moreover, a recorded ^1^H DOSY spectrum confirmed the clear formation of one single species (Figure S35). It is worth noting that, to the best of our knowledge, this is the first example of a non-statistical self-assembly of a [Pd_2_L^A^_3_L^B^] heteroleptic cage. In addition, we explored the possibility of using also the photoisomeric *o*-**B** as a platform for the formation of heteroleptic cages. For this purpose, **L**^**d**^ and **L**^**f**^ were tested as fourth ligands. After addition of 1.0 equiv of the respective ligand to a solution of *o*-**B** at room temperature, ^1^H NMR spectra of the resulting compounds were found to suffer from signal broadening, pointing at a highly dynamic behavior of the system. Interestingly, NMR signals turned sharp, allowing clear assignment, after guest **G1** was added and apparently encapsulated (Figure 4b, blue/yellow panel, Figures S46 and S50). HR-ESI-MS spectra revealed prominent peaks consistent with the formula [Pd_2_(*o*-**L**^**a**^)_3_**L**^**d,f**^(BF_4_)_*n*_]^(4–*n*)+^ (*n* = 1–2), even in the absence of the guest, suggesting that the formation of heteroleptic cages *o*-**B**-**L**^**d,f**^ from *o*-**B** and **L**^**d**^ or **L**^**f**^ proceeds smoothly, despite the difficult-to-analyze NMR spectra (Figures S49 and S51).

**Figure 6:**
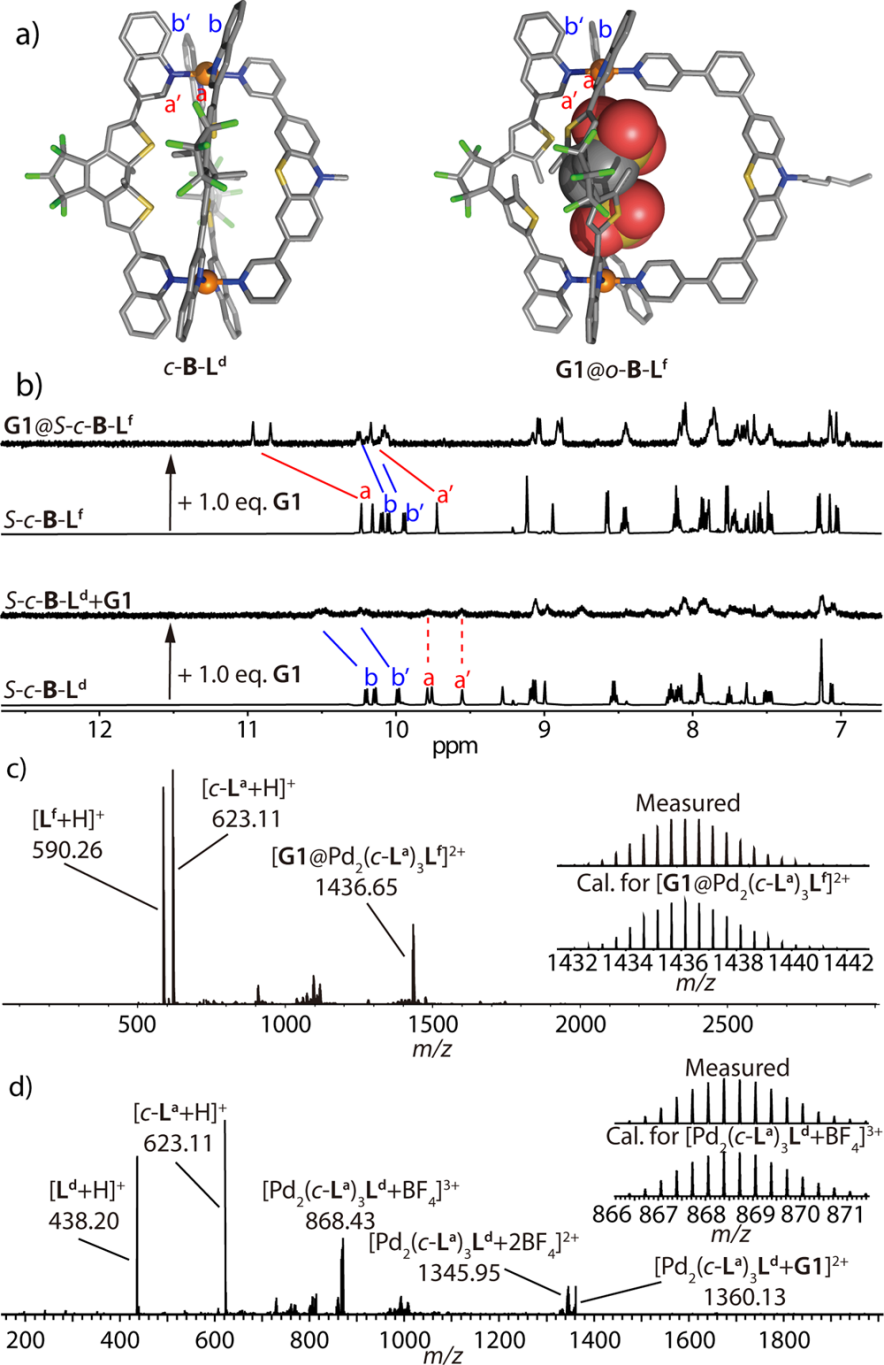
(a) DFT-optimized structure of *c*-**B**-**L**^**d**^ (left) and X-ray structure of **G1**@*o*-**B**-**L**^**f**^ (right). (b) ^1^H NMR spectra (500 MHz, CD_3_CN, 298 K) of homochiral *S*-*c*-**B**-**L**^**d**^ and *S*-*c*-**B**-**L**^**f**^, and their corresponding spectra after addition of 1.0 equiv of **G1**. (c, d) ESI-MS spectra of (c) **G1**@*o*-**B**-**L**^**f**^, with isotope pattern of [**G1**@Pd_2_(*c*-**L**^**a**^)_3_**L**^**f**^]^2+^ shown in the inset, and (d) *c*-**B**-**L**^**d**^ after addition of 1.0 equiv of **G1**, with isotope pattern of [Pd_2_(*c*-**L**^**a**^)_3_**L**^**d**^ + BF_4_]^3+^.

Next, we investigated light-triggered switching between the two photoisomeric heteroleptic assemblies, *o*-**B**-**L**^**d**^ and *c*-**B**-**L**^**d**^ (Figure S111). Irradiation of *c*-**B**-**L**^**d**^ under 617 nm light led to the same ^1^H NMR spectrum as obtained for the self-assembly of *o*-**B**-**L**^**d**^ from *o*-**B** and **L**^**d**^. Conversely, irradiation of cage *o*-**B**-**L**^**d**^ with light of 313 nm wavelength restored the original ^1^H NMR spectrum, showing full reversibility between the closed and open heteroleptic cages. Despite numerous attempts, we were not able to obtain single crystals suitable for X-ray analysis for any of the guest-free heteroleptic cages. To help understanding their structural features, we performed DFT (ωB97XD/LanL2DZ) geometry optimizations (Figure 6a, Figure S59). The models reveal a notable enlargement of the cavity volume for *c*-**B**-**L**^**f**^ as compared to *c*-**B**-**L**^**d**^. We therefore assumed that this should lead to a distinguishable guest uptake behavior.

As described above, the interaction of the three guest molecules, **G1**, **G2**, and **G3**, with these heteroleptic cages was characterized by means of ^1^H NMR titration experiments. The addition of **G1** to the smaller cage *c*-**B**-**L**^**d**^ resulted in shifting of proton signals of H_b_ and H_b′_, pointing outside from the cage cavity, while the inward-looking protons did not experience any shifting (Figure 6b). Furthermore, ESI-MS analysis only indicates unspecific association with the anionic guest (Figure 6d). The same result was obtained using **G2** and **G3**, suggesting for all three guests merely interaction via the outside of the cage structure (Figures S77–S79). It is worth emphasizing that, as shown above, bowl *c*-**B** is able to bind all three guest molecules, while heteroleptic cage *c*-**B**-**L**^**d**^ does not. On the other hand, all three guests could be encapsulated inside cage *c*-**B**-**L**^**f**^ (Figure 4b, yellow panel). Hence, the larger fourth ligand **L**^**f**^ (carrying phenylene spacers between backbone and coordinating pyridines as opposed to smaller **L**^**d**^) allows the inner cavity to be sufficiently large to bind guest molecules, as confirmed by ^1^H NMR and HR-ESI-MS experiments (Figure 6b,c). After addition of **G1** into a solution of *c*-**B**-**L**^**f**^, a new set of ^1^H NMR signals appeared (Figure 6b). Further confirmation came from ESI-MS, where a peak assigned to [**G1**@*c*-**B**-**L**^**f**^]^2+^ was clearly detected (Figure 6c), indicating a 1:1 host–guest stoichiometry. Also, NMR titrations with **G2** and **G3** show shifting of the inward-pointing protons H_a_ and H_a′_ until 1.0 equiv of the guest was added (Figures S82 and S84). Addition of more guest equivalents led to interaction with the cage’s outer side, as confirmed by shifting of the signals of outward-pointing protons H_b_ and H_b′_, followed by precipitation of the sample. The formation and stoichiometry of the host–guest complexes with **G2** and **G3** were further confirmed by ESI-MS (Figures S83 and S85). Finally, guest affinity was also investigated for cage *c*-**B**-**L**^**e**^, leading to results comparable to those obtained for *c*-**B**-**L**^**f**^ (Figures S86–S91).

Interestingly, the host–guest behavior of the *o*-**B**-**L** cages is different from that of the analogous assemblies with the closed-form DTE photoswitch, presumably due to the higher flexibility of the backbone in its open photoisomeric form. Thus, addition of **G1** to the small-cavity cage *o*-**B**-**L**^**d**^ led to the formation of a [**G1**@*o*-**B**-**L**^**d**^] host–guest compound. While the NMR spectrum of *o*-**B**-**L**^**d**^ is characterized by broad signals, stepwise titration with **G1** led to a new set of sharp signals, showing at the same time guest uptake and stabilization of a more defined conformer of the host (Figure S92). This allowed a better characterization of the system with 2D NMR spectra (Figures S47 and S48), and therefore an indirect confirmation of the *o*-**B**-**L**^**d**^ heteroleptic cage assembly. Further support came from ESI-MS analysis, with a prominent peak assigned to [**G1**@*o*-**B**-**L**^**d**^]^2+^, confirming once more the binding of **G1** with a 1:1 stoichiometry (Figure S93). Photoswitching of **G1**@*o*-**B**-**L**^**d**^ triggers guest release to give *c*-**B**-**L**^**d**^, for which NMR results indicate only outside guest interaction (Figure S113 compared to Figure S77). Titration of *o*-**B**-**L**^**d**^ with the bigger guests **G2** and **G3** showed no signs of encapsulation, comparable to the closed-ligand cage analogue (Figures S94 and S95). On the other hand, when using heteroleptic cage derivative *o*-**B**-**L**^**f**^, again possessing a larger cavity size, uptake of guest **G1** was certainly anticipated and indeed confirmed by ^1^H NMR titration and ESI-MS. As for the shorter analogue, the titration with **G1** led to a new set of ^1^H NMR signals, converting the broad signals of the empty cage into a better resolved NMR spectrum (Figure S96). HR-ESI-MS proved a 1:1 binding stoichiometry, showing only the peaks of [**G1**@*o*-**B**-**L**^**f**^]^2+^ (Figure S97). Addition of an excess of guest (3 equiv) led to precipitate formation, together with the growth of single crystals suitable for X-ray analysis (Figure 6a). The compound crystallizes in monoclinic space group *P*2_1_/*n* and was confirmed to be **G1**@*o*-**B-L**^**f**^. The structure clearly shows the formation of a [Pd_2_L^A^_3_L^B^] heteroleptic cage consisting of three ligands *o*-**L**^**a**^ and one ligand **L**^**f**^, where the pyridines of **L**^**f**^ saturate the coordination spheres of the Pd^II^ ions. **G1** is found to sit inside the cavity, in full agreement with the NMR and MS data.

Unfortunately, experiments probing the uptake of the bigger guests **G2** and **G3** inside *o*-**B**-**L**^**f**^ were complicated by concomitant cage disassembly. Titration with **G2** indeed showed the appearance of a new set of weak ^1^H NMR signals, however, accompanied by release of free ligand **L**^**f**^ (Figure S98), suggesting partial decomposition of the cage. Titration with **G3** led to decomposition of *o*-**B**-**L**^**f**^ right from the beginning of the guest addition (Figure S100).

**Figure 7:**
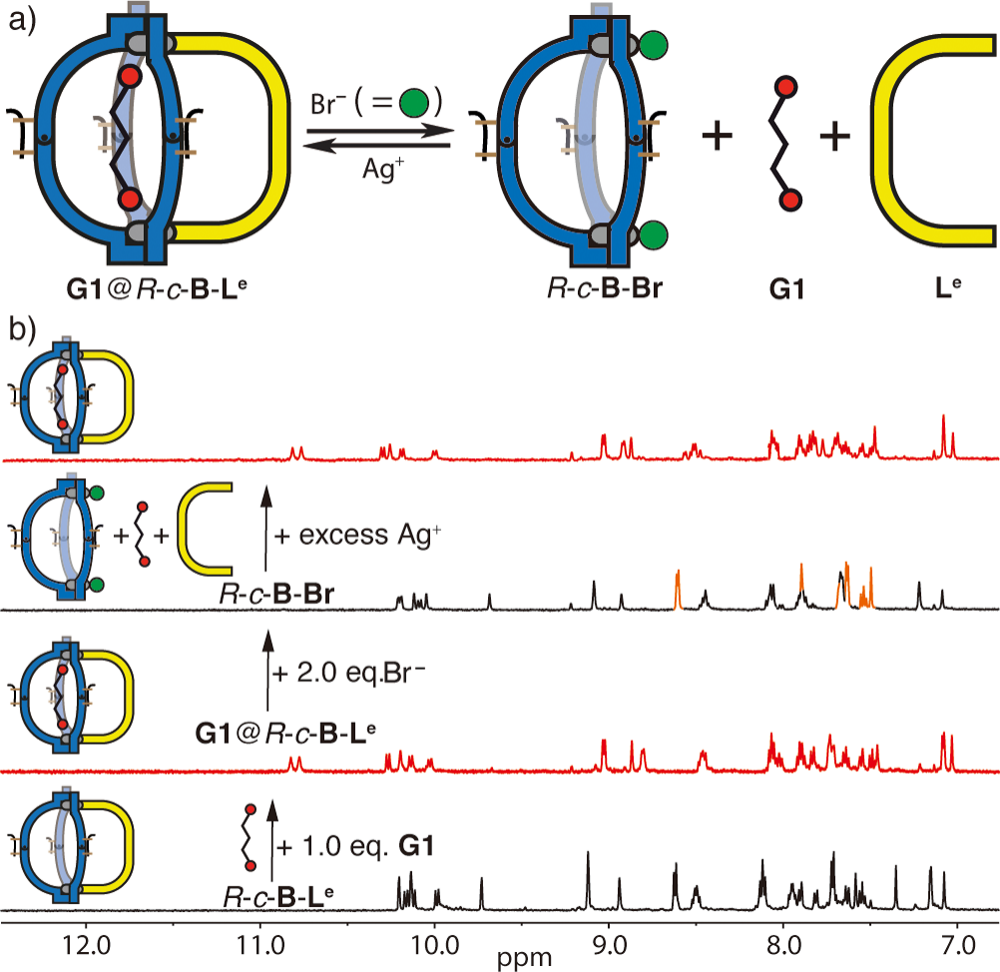
(a) Scheme and (b) ^1^H NMR spectra (500 MHz, CD_3_CN, 298 K) showing how addition of Br^–^/Ag^+^ triggers the reversible release and binding of **G1** from the heteroleptic cage. The ^1^H NMR spectra show, from bottom to top: *R*-*c*-**B**-**L**^**e**^; addition of 1.0 equiv of **G1**, resulting in **G1**@*R-c*-**B**-**L**^**e**^ (red marked); addition of 2.0 equiv of Br^–^, releasing “free” ligand **L**^**e**^ (orange signals) and **G1** simultaneously, thus forming *R-c*-**B**-**Br**; and addition of an excess of Ag^+^ (up to 20 equiv), removing the halide anions and restoring the heteroleptic host–guest complex **G1**@*R-c*-**B**-**L**^**e**^.

Finally, we explored the possibility to use halide anions as an external stimulus and competitor, either to the solvent molecules or to the pyridine-based ligands occupying the fourth Pd^II^ coordination sites (Figure 1a, green panel). Following this assumption, 2.0 equiv of Cl^–^ or Br^–^ (NBu_4_^+^ as countercation) was added to a *c*-**B** solution. The clean formation of [Pd_2_(*c*-**L**^**a**^)_3_(Cl/Br)_2_](BF_4_)_2_ (*c*-**B-Cl**/**Br**) was confirmed by a combination of NMR and HR-ESI-MS experiments (Figures S18–S25). Interestingly, this stimulus could be reversed by addition of a Ag^+^ salt (AgBF_4_), leading to recovery of the initial bowl compound (Figure S115). Hence, the two species can reversibly interconvert under chemical control: the addition and removal of halide ions trigger the formation of either *c*-**B-Cl/Br** or *c*-**B**, respectively. The same concept can be applied to the host–guest complexes **G**@*c*-**B**. As coordination of the halide anions affects the overall charge of the supramolecular compound, going from a +4 charged species to a +2 cationic assembly, the induction of guest release was the consequence (Figure 4b, green panel). For example, addition of 2 equiv of Br^–^ to a solution of enantiomerically pure **G2**@*R-c*-**B** forms the species *R-c*-**B-Br** and triggers release of the guest from the bowl’s pocket, as confirmed by ^1^H NMR spectroscopy. Again, this process is reversible: addition of Ag^+^ cations led to precipitation of AgBr salt and restoration of the **G2**@*R-c*-**B** host–guest compound, as confirmed by ^1^H NMR spectroscopy (Figure S117). Subsequently, we applied this approach to the heteroleptic cages [Pd_2_L^A^_3_L^B^] and corresponding host–guest compounds. Rewardingly, a reversible structural conversion between the heteroleptic cages and *c*-**B-Br** bowl is achieved by addition of bromide and silver ions (Figure 1a, yellow and green panels). Taking *c*-**B**-**L**^**e**^ as an example, the cage was disassembled after addition of 2.0 equiv of Br^–^ into a *c*-**B**-**L**^**e**^ solution, as clearly seen from ^1^H NMR spectra (Figure S118), giving rise to *c*-**B**-**Br** and releasing free **L**^**e**^ at the same time. In this case, a stoichiometric amount of Ag^+^ (2.0 equiv) was not enough to restore the starting heteroleptic cage. However, adding an excess amount of silver salt (up to 10 equiv) into the mixture at room temperature allowed us to reassemble cage *c*-**B**-**L**^**e**^. This approach can be extended to an even more complex system, where four components (Pd^II^, **L**^**a**^, **L**^**e**^, **G1**) are assembled through integrative self-sorting to give the host–guest system **G1**@*c*-**B**-**L**^**e**^. Herein, the combination of guest uptake properties and chemical stimuli led to a reversible on/off guest binding event (Figure 7a). Upon addition of 2.0 equiv of Br^–^ to a **G1**@*c*-**B**-**L**^**e**^ solution, the ^1^H NMR spectra showed the complete release of **G1** and the decomplexation of ligand **L**^**e**^, together with the formation of *c*-**B**-**Br** (Figure 7b). Subsequently, addition of an excess of Ag^+^ allows the complex mixture to reassemble, forming selectively only the host–guest complex **G1**@*c*-**B**-**L**^**e**^ following a non-statistical integrative self-sorting process (Figure 7b).

## Conclusions

In summary, the appropriate design of a photochromic ditopic banana-shaped DTE-based ligand, **L**^**a**^, carrying bulky quinoline donor groups, allowed us to combine light-switchable backbone flexibility with steric hindrance around a Pd^II^ coordination center, leading to a multi-stimuli-responsive system. Self-assembly of **L**^**a**^ photoisomers with Pd^II^ ions leads to the controlled formation of a coordination cage, *o*-**C**, or a bowl-shaped object, *c*-**B**, reversibly interconvertible by irradiation with light of different wavelengths. Their structural diversity results in a different guest binding behavior: compared to the small pocket of cage *o*-**C**, bowl *c*-**B** has a larger cavity for guest uptake. Moreover, the bowl structure can act as platform to form an unprecedented type of [Pd_2_L^A^_3_L^B^] heteroleptic cages in a non-statistical way. Proper design of the fourth ligand, alone or in combination with the light stimulus, allowed us to further tune the guest binding affinity. Finally, the addition of halide and silver ions led to a reversible, charge-modulating stimulus to trigger on/off guest binding. Herein, we report a new strategy to form heteroleptic, multi-functional cages, where the host–guest properties derive from the synergy of all contained building blocks: a photochromic ligand backbone, a quinoline-based, congested coordination environment, and a fourth ligand coming in different sizes. This generates a multi-stimuli-responsive system where light, halides, and additional ligands act either individually or in a cooperative fashion.

## Supporting Information Available

Synthetic procedures, NMR, MS, X-ray, further spectroscopic data, and DFT calculation results, including Schemes S1–S14, Figures S1–S125, and Tables S1–S4 (PDF)

## Accession Codes

CCDC 2003429–2003433 contain the supplementary crystallographic data for this paper. These data can be obtained free of charge via www.ccdc.cam.ac.uk/data_request/cif, or by emailing data_request@ccdc.cam.ac.uk, or by contacting The Cambridge Crystallographic Data Centre, 12 Union Road, Cambridge CB2 1EZ, UK; fax: +44 1223 336033.

## References

[ref1] aWu G.-Y.; Chen L.-J.; Xu L.; Zhao X.-L.; Yang H.-B. Construction of Supramolecular Hexagonal Metallacycles via Coordination-Driven Self-Assembly: Structure, Properties and Application. Coord. Chem. Rev. 2018, 369, 39–75. 10.1016/j.ccr.2018.05.009.

[ref2] aCook T. R.; Stang P. J. Recent Developments in the Preparation and Chemistry of Metallacycles and Metallacages via Coordination. Chem. Rev. 2015, 115, 7001–7045. 10.1021/cr5005666. 25813093

[ref3] aWang L.; Song B.; Khalife S.; Li Y.; Ming L.-J.; Bai S.; Xu Y.; Yu H.; Wang M.; Wang H.; Li X. Introducing Seven Transition Metal Ions into Terpyridine-Based Supramolecules: Self-Assembly and Dynamic Ligand Exchange Study. J. Am. Chem. Soc. 2020, 142, 1811–1821. 10.1021/jacs.9b09497. 31910337 PMC7375339

[ref4] aYamashina M.; Tanaka Y.; Lavendomme R.; Ronson T. K.; Pittelkow M.; Nitschke J. R. An Antiaromatic-Walled Nanospace. Nature 2019, 574, 511–515. 10.1038/s41586-019-1661-x. 31645731

[ref5] aRizzuto F. J.; von Krbek L. K. S.; Nitschke J. R. Strategies for Binding Multiple Guests in Metal–Organic Cages. Nat. Rev. Chem. 2019, 3, 204–222. 10.1038/s41570-019-0085-3.

[ref6] aLeenders S. H. A. M.; Gramage-Doria R.; de Bruin B.; Reek J. N. H. Transition Metal Catalysis in Confined Spaces. Chem. Soc. Rev. 2015, 44, 433–448. 10.1039/C4CS00192C. 25340992

[ref7] aRizzuto F. J.; Kieffer M.; Nitschke J. R. Quantified Structural Speciation in Self-Sorted Co^II^_6_L4 Cage Systems. Chem. Sci. 2018, 9, 1925–1930. 10.1039/C7SC04927G. 29719682 PMC5894586

[ref8] Yamashina M.; Yuki T.; Sei Y.; Akita M.; Yoshizawa M. Anisotropic Expansion of an M2L4 Coordination Capsule: Host Capability and Frame Rearrangement. Chem. - Eur. J. 2015, 21, 4200–4204. 10.1002/chem.201406445. 25677602

[ref9] Preston D.; Barnsley J. E.; Gordon K. C.; Crowley J. D. Controlled Formation of Heteroleptic [Pd2(L a)2(Lb)2]^4+^ Cages. J. Am. Chem. Soc. 2016, 138, 10578–10585. 10.1021/jacs.6b05629. 27463413

[ref10] aLi J.-R.; Zhou H.-C. Bridging-ligand-substitution strategy for the preparation of metal–organic polyhedral. Nat. Chem. 2010, 2, 893–898. 10.1038/nchem.803. 20861907

[ref11] Zhu R.; Bloch W. M.; Holstein J. J.; Mandal S.; Schäfer L.; Clever G. H. Donor-Site-Directed Rational Assembly of Heteroleptic cis-[Pd2L2L2] Coordination Cages from Picolyl Ligands. Chem. - Eur. J. 2018, 24, 12976–12982. 10.1002/chem.201802188. 29924444 PMC6174927

[ref12] aChen B.; Holstein J. J.; Horiuchi S.; Hiller W. G.; Clever G. H. Pd(II) Coordination Sphere Engineering: Pyridine Cages, Quinoline Bowls, and Heteroleptic Pills Binding One or Two Fullerenes. J. Am. Chem. Soc. 2019, 141, 8907–8913. 10.1021/jacs.9b02207. 31067401 PMC6609009

[ref13] aBloch W. M.; Clever G. H. Integrative Self-Sorting of Coordination Cages Based on ‘Naked’ Metal Ions. Chem. Commun. 2017, 53, 8506–8516. 10.1039/C7CC03379F. PMC5672845 28661517

[ref14] aGu Y.; Alt E. A.; Wang H.; Li X.; Willard A. P.; Johnson J. A. Photoswitching Topology in Polymer Networks with Metal–Organic Cages as Crosslinks. Nature 2018, 560, 65–69. 10.1038/s41586-018-0339-0. 30022167

[ref15] Kim T. Y.; Vasdev R. A. S.; Preston D.; Crowley J. D. Strategies for Reversible Guest Uptake and Release from Metallosupramolecular Architectures. Chem. - Eur. J. 2018, 24, 14878–14890. 10.1002/chem.201802081. 29939443

[ref16] aKishi N.; Akita M.; Yoshizawa M. Selective Host–Guest Interactions of a Transformable Coordination Capsule/Tube with Fullerenes. Angew. Chem., Int. Ed. 2014, 53, 3604–3607. 10.1002/anie.201311251. 24590625

[ref17] aLöffler S.; Lübben J.; Krause L.; Stalke D.; Dittrich B.; Clever G. H. Triggered Exchange of Anionic for Neutral Guests inside a Cationic Coordination Cage. J. Am. Chem. Soc. 2015, 137, 1060–1063. 10.1021/ja5130379. 25569812

[ref18] aCroué V.; Goeb S.; Szalóki G.; Allain M.; Sallé M. Reversible Guest Uptake/Release by Redox-Controlled Assembly/Disassembly of a Coordination Cage. Angew. Chem., Int. Ed. 2016, 55, 1746–1750. 10.1002/anie.201509265. 26693832

[ref19] aXu L.; Zhang D.; Ronson T. K.; Nitschke J. R. Improved Acid Resistance of a Metal–Organic Cage Enables Cargo Release and Exchange between Hosts. Angew. Chem., Int. Ed. 2020, 59, 7435–7438. 10.1002/anie.202001059. PMC7217015 32073709

[ref20] Jansze S. M.; Cecot G.; Severin K. Reversible Disassembly of Metallasupramolecular Structures Mediated by a Metastable-State Photoacid. Chem. Sci. 2018, 9, 4253–4257. 10.1039/C8SC01108G. 29780555 PMC5944229

[ref21] aKathan M.; Hecht S. Photoswitchable molecules as key ingredients to drive systems away from the global thermodynamic minimum. Chem. Soc. Rev. 2017, 46, 5536–5550. 10.1039/C7CS00112F. 28857096

[ref22] aHan M.; Michel R.; He B.; Chen Y.; Stalke D.; John M.; Clever G. H. Light-Triggered Guest Uptake and Release by a Photochromic Coordination Cage. Angew. Chem., Int. Ed. 2013, 52, 1319–1323. 10.1002/anie.201207373. 23208865

[ref23] aWezenberg S. J. Light-switchable Metal-Organic Cages. Chem. Lett. 2020, 49, 609–615. 10.1246/cl.200076.

[ref24] Our previous work has demonstrated, however, that the non-configurationally stable open-form ligands within the cage architecture can quickly epimerize in solution. See ref (22).

