# Supplementary material for: Multi-stimuli Control over Assembly and Guest Binding in Metallo-supramolecular Hosts Based on Dithienylethene Photoswitches
Source: J Am Chem Soc. 2021 Mar 5;143(10):3865–73. doi: 10.1021/jacs.0c12188 (PMC7975281; doi:10.1021/jacs.0c12188)
Supplement: Supplementary file 1 — ja0c12188_si_001.pdf [file ja0c12188_si_001.pdf]

Multi-Stimuli Control over Assembly and Guest Binding in Metallo-Supramolecular Hosts based on DTE Photoswitches

Ru-Jin Li,†,‡ Jacopo Tessarolo,†,‡ Haeri Lee,†,§ and Guido H. Clever*,†

†Faculty of Chemistry & Chemical Biology, TU Dortmund University, Otto Hahn Str. 6, 44227 Dortmund, Germany.

Supporting Information

Contents

1. General procedures 2

2. Synthesis 2

2.1. Ligand synthesis 2

2.1.1 Synthesis of 3,3'-((perfluorocyclopent-1-ene-1,2-diyl)bis(5-methylthiophene-4,2-diyl))diquinoline (o-$\mathbf{L}^a$) 2

2.1.2 Synthesis of 4,4'-(((perfluorocyclopent-1-ene-1,2-diyl)bis(5-methylthiophene-4,2-diyl))bis(3,1-phenylene))dipyridine (o-$\mathbf{L}^c$) 3

2.1.3 Synthesis of 4,4'-(((perfluorocyclopent-1-ene-1,2-diyl)bis(5-methylthiophene-4,2-diyl))bis(3,1-phenylene))dipyridine ($\mathbf{L}^f$) 5

2.2. Cage synthesis 7

2.2.1 Synthesis of $[Pd_2(o\text{-}\mathbf{L}^a)_4](BF_4)_4$ (o-**C**) 7

2.2.2 Synthesis of $[Pd_2(c\text{-}\mathbf{L}^a)_3(Solvent)_2](BF_4)_4$ (c-**B**) 8

2.2.3 Synthesis of $[2Cl@Pd_2(c\text{-}\mathbf{L}^a)_3](BF_4)_2$ (c-**B-Cl**) 12

2.2.4 Synthesis of $[2Br@Pd_2(c\text{-}\mathbf{L}^a)_3](BF_4)_2$ (c-**B-Br**) 14

2.3. Heteroleptic cage synthesis 17

2.3.1 Synthesis of $[Pd_2(c\text{-}\mathbf{L}^a)_3(o\text{-}\mathbf{L}^c)_3](BF_4)_4$ (c-**B**-$\mathbf{L}^c$) 17

2.3.2 Synthesis of $[Pd_2(c\text{-}\mathbf{L}^a)_3\mathbf{L}^d](BF_4)_4$ (c-**B**-$\mathbf{L}^d$) 20

2.3.3 Synthesis of $[Pd_2(c\text{-}\mathbf{L}^a)_3(o\text{-}\mathbf{L}^e)](BF_4)_4$ (c-B-$\mathbf{L}^e$) 22

2.3.4 Synthesis of $[Pd_2(c\text{-}\mathbf{L}^a)_3\mathbf{L}^f](BF_4)_4$ (c-**B**-$\mathbf{L}^f$) 25

3. Symmetry 31

4. Absolute configuration 32

5. Computational studies 35

5.1 Optimized structure of heteroleptic cages 36

5.2 Comparison of the energy between cage and bowl structures 36

6. Titrations 37

7. Photoswitching 56

8. Stability of *c*-B 61

9. Structure interconversion 62

10. X-Ray data 64

10.1. Data Collection 64

10.2. Refinement Details 67

10.3. Structural analysis 67

11. UV-vis spectroscopy 69

12. References 70

1. General procedures

All chemicals, except otherwise specified, were obtained from commercial sources, and used without further purification. Perfluoro-1,2-bis(2-iodo-5-methylthien-4-yl)cyclopentene[S1], $\mathbf{L}^{c}$,[S2] $\mathbf{L}^{d}$[S3] were prepared according to a literature procedure. The tetrabutylammonium salts of the guests (**G1**, **G2** and **G3**) were synthesized according to a previously reported procedure. [S4] Corresponding starting materials were obtained commercially as sodium salt or free sulfonic acids in the highest available purity. Recycling gel permeation chromatography was performed on a JAI LC-9210 II NEXT GPC system equipped with Jaigel 1H and 2H columns in series using chloroform as the eluent (HPLC grade). NMR measurements were all conducted at 298 K on Avance-500, Avance-600 and Avance-700 instruments from Bruker and INOVA 500 MHz machine from Varian. High-resolution ESI mass spectrometric measurements were carried out on maXis ESI-TOF MS, Compact and ESI-timsTOF machines from Bruker. Irradiations at 313 nm were performed by placing a quartz NMR tube in a distance of 5 cm in front of a 300 W Hg arc lamp from LOT-Oriel equipped with a dichroic mirror and 313 nm bandpass filter. Irradiations at 617 nm were performed by placing a quartz NMR tube 2.5 cm in front of a LED irradiation apparatus (3x 1.4 W 617 nm Power LED, 25 nm FWHM) from Sahlmann Photonics, Kiel. Chiral HPLC was performed on an Agilent Technologies 1260 infinity HPLC system equipped with Daicel CHIRALPAK IC columns (250 x 4.6 mm and 250 x 10 mm) using a dichloromethane/hexane/methanol (39%/60%/1%) mixture as eluent for the separation of *c*-$\mathbf{L}^{a}$. UV-Vis spectra were recorded on an Agilent 8453 UV-Visible spectrophotometer. CD spectra were measured on an Applied Photophysics Chirascan circular dichroism spectrometer. All heteroleptic cage and *c*-**B** models were constructed using SPARTAN[S5] and were first optimized on a PM6 level of theory (no counter ions were included) without constrains. The resulting structures were than further refined by DFT calculations (B3LYP/LANL2DZ) using Gaussian 09 software.[S6]

2. Synthesis

2.1. Ligand synthesis

2.1.1 Synthesis of 3,3'-((perfluorocyclopent-1-ene-1,2-diyl)bis(5-methylthiophene-4,2-diyl))diquinoline (o-$\mathbf{L}^{a}$)

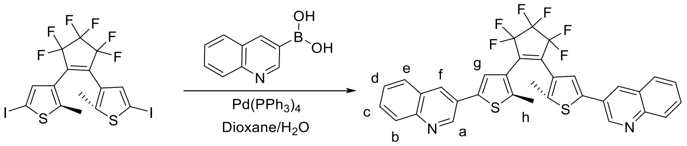

Scheme S1 Synthesis of *o*-$\mathbf{L}^{a}$.

A mixture of perfluoro-1,2-bis(2-iodo-5-methylthien-4-yl)cyclopentene[S1] (310 mg, 0.5 mmol), quinolin-3-ylboronic acid (259.6 mg, 1.5 mmol), $Pd(PPh_3)_4$ (34.7 mg, 0.03 mmol), K_3PO_4 (1.73 g, 7.5 mmol) and

degassed dioxane/H_2O (30 mL V/V = 1:1) were combined in an oven-dried Schlenk tube and stirred at 90 °C for overnight. After cooling down to room temperature, CH_2Cl_2 was added, and the solution was washed with water and brine, dried over anhydrous $MgSO_4$ and concentrated *in vacuo*. The crude product was separated through column chromatography (silica) using CH_2Cl_2/MeOH (50:1) as eluents and further purified by GPC using $CHCl_3$ as eluents to give pure *o*-**L**$^{\mathbf{a}}$ (199.3 mg, 0.32 mmol) as a bluish solid in 64% yield. ^{1}H NMR (500 MHz, acetonitrile-*d*$_3$) δ 9.17 (d, *J* = 2.4 Hz, 2H), 8.42 (d, *J* = 2.4 Hz, 2H), 8.04 (d, *J* = 8.5 Hz, 2H), 7.93 (d, *J* = 8.0 Hz, 2H), 7.74 (ddd, *J* = 8.4, 6.9, 1.5 Hz, 2H), 7.65 (s, 2H), 7.64 – 7.59 (m, 2H), 2.11 (s, 6H). ^{13}C NMR (151 MHz, acetonitrile-*d*$_3$) δ 149.05, 148.37, 144.13, 140.02, 132.21, 130.65, 130.04, 129.11, 128.73, 128.42, 127.33, 126.71, 125.25, 14.88 (three signals missing due to ^{19}F coupling effects as described previously[S15]). ^{19}F NMR (471 MHz, acetonitrile-*d*$_3$) δ -110.75 (t, *J* = 5.2 Hz), -132.49 (q, *J* = 5.1 Hz). ESI-HRMS calculated for $C_{33}H_{21}F_6N_2S_2$ ($[M+H]^+$) *m/z* = 623.1050, found *m/z* = 623.1025.

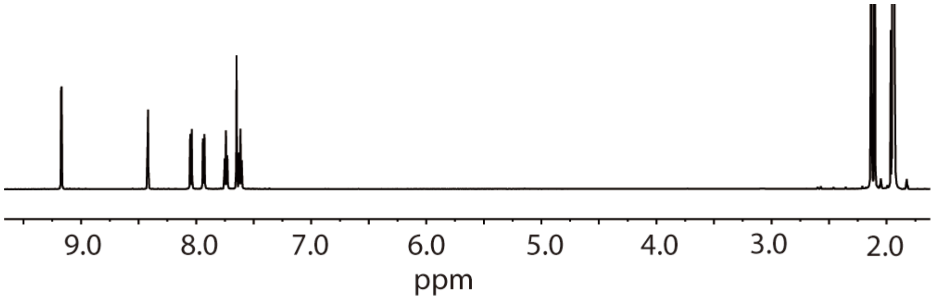

Figure S1. ^{1}H NMR spectrum (500 MHz, acetonitrile-*d*$_3$) of *o*-**L**$^{\mathbf{a}}$.

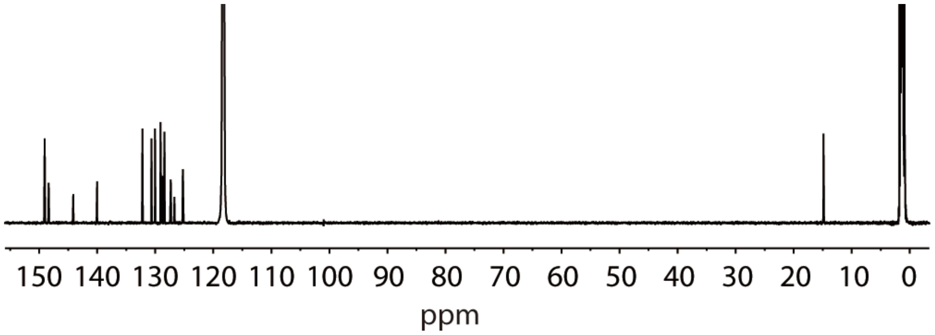

Figure S2. ^{13}C NMR spectrum (151 MHz, acetonitrile-*d*$_3$) of *o*-**L**$^{\mathbf{a}}$.

*2.1.2 Synthesis of 4,4'-(((perfluorocyclopent-1-ene-1,2-diyl)bis(5-methylthiophene-4,2-diyl))bis(3,1-phenylene))dipyridine (o-**L**^{e})*

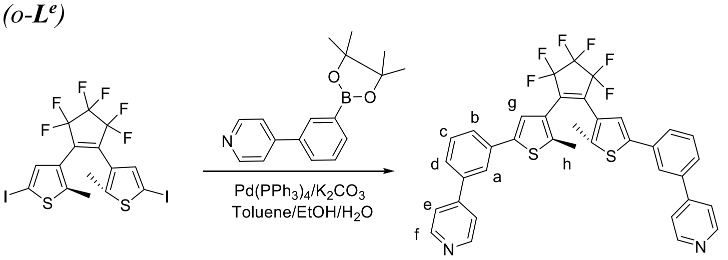

Scheme S2 Synthesis of *o*-**L**$^{\mathbf{e}}$.

A mixture of perfluoro-1,2-bis(2-iodo-5-methylthien-4-yl)cyclopentene (310 mg, 0.5 mmol), 4-(3-(4,4,5,5-tetramethyl-1,3,2-dioxaborolan-2-yl)phenyl)pyridine (421.7 mg, 1.5 mmol), $Pd(PPh_3)_4$ (34.7 mg, 0.03 mmol), K_2CO_3 (1.1 g, 8.0 mmol) and degassed toluene/EtOH/H_2O (30 mL V/V/V = 2:1:1) were combined in an oven-dried Schlenk tube and stirred at 90 °C for overnight. After cooling down to room temperature, CH_2Cl_2 was added, and the solution was washed with water and brine, dried over anhydrous $MgSO_4$ and concentrated *in vacuo*. The crude product was separated through column chromatography (silica) using CH_2Cl_2/MeOH (40:1) as eluents and further purified by GPC ($CHCl_3$ as eluents) to give pure *o*-$\mathbf{L^e}$ (124.3 mg, 0.2 mmol) as a bluish solid in 36.8% yield. ^1H NMR (500 MHz, acetonitrile-*d₃*) δ 8.63 – 8.59 (m, 4H), 7.90 (t, *J* = 1.8 Hz, 2H), 7.68 (p, *J* = 1.1 Hz, 2H), 7.66 (t, *J* = 2.0 Hz, 2H), 7.65 – 7.62 (m, 4H), 7.54 (t, *J* = 7.7 Hz, 2H), 7.50 (s, 2H), 2.07 (s, 6H). ^{13}C NMR (151 MHz, acetonitrile-d_3) δ 151.26, 148.12, 143.27, 142.47, 139.88, 134.93, 130.97, 127.62, 127.17, 126.51, 124.96, 124.61, 122.54, 14.76 (three signals missing due to ^{19}F coupling effects as described previously[S15]). ^{19}F NMR (471 MHz, chloroform-*d*) δ -109.94 (d, *J* = 5.6 Hz), -131.85. ESI-HRMS calculated for $C_{37}H_{25}F_6N_2S_2$ $[M+H]^+$ *m/z* = 675.1358, found *m/z* = 675.1368.

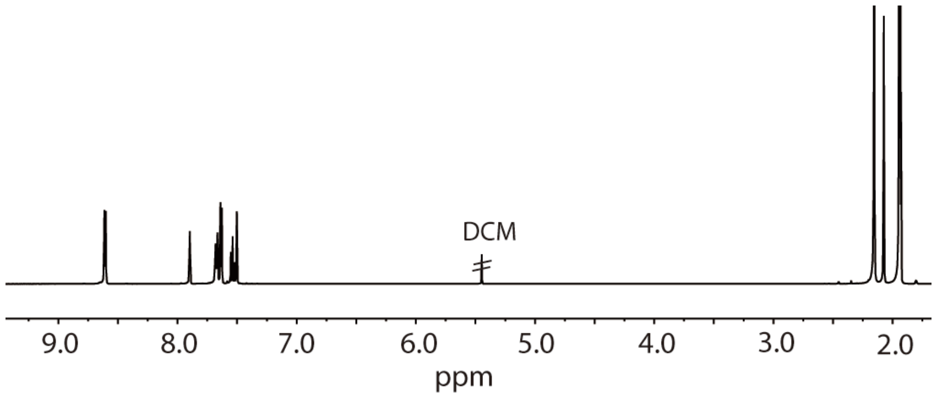

Figure S3. ^1H NMR spectrum (500 MHz, acetonitrile-d_3) of *o*-$\mathbf{L^e}$.

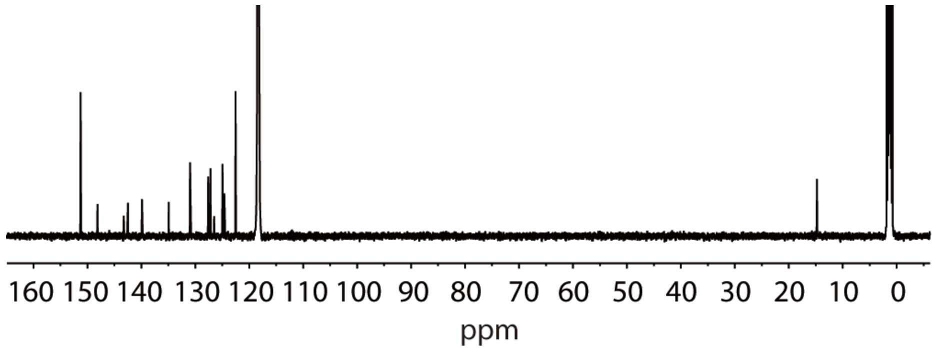

Figure S4. ^{13}C NMR spectrum (151 MHz, acetonitrile-d_3) of *o*-$\mathbf{L^e}$.

2.1.3 Synthesis of *4,4'-(((perfluorocyclopent-1-ene-1,2-diyl)bis(5-methylthiophene-4,2-diyl))bis(3,1-phenylene))dipyridine* (***L***^{f})

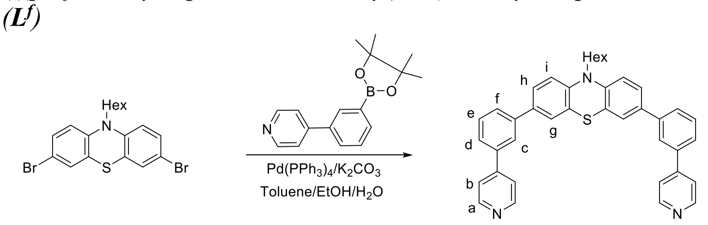

Scheme S3 Synthesis of **L**$^{\mathbf{f}}$.

A mixture of 3,7-dibromo-10-hexyl-10*H*-phenothiazine (220.6 mg, 0.5 mmol), 4-(3-(4,4,5,5-tetramethyl-1,3,2-dioxaborolan-2-yl)phenyl)pyridine (421.7 mg, 1.5 mmol), $Pd(PPh_3)_4$ (34.7 mg, 0.03 mmol), K_2CO_3 (1.1 g, 8.0 mmol) and degassed toluene/EtOH/H_2O (30 mL V/V/V = 2:1:1) were combined in an oven-dried Schlenk tube and stirred at 90 °C for overnight. After cooling down to room temperature, CH_2Cl_2 was added, and the solution was washed with water and brine, dried over anhydrous $MgSO_4$ and concentrated *in vacuo*. The crude product was separated by column chromatography (silica) using CH_2Cl_2/MeOH (40:1) as eluents and further purified through GPC to give pure **L**$^{\mathbf{f}}$ (253.7 mg, 0.4 mmol) as a yellow solid in 86% yield. ^{1}H NMR (500 MHz, chloroform-*d*) δ 8.79 – 8.64 (m, 4H), 7.80 (t, *J* = 1.8 Hz, 2H), 7.65 (ddd, *J* = 6.1, 3.5, 1.6 Hz, 6H), 7.61 (dt, *J* = 7.7, 1.5 Hz, 2H), 7.57 (t, *J* = 7.6 Hz, 2H), 7.47 (d, *J* = 2.2 Hz, 2H), 7.44 (d, *J* = 2.3 Hz, 2H), 6.99 (d, *J* = 8.2 Hz, 2H), 3.94 (t, *J* = 7.2 Hz, 2H), 1.96 – 1.83 (m, 2H), 1.57 – 1.46 (m, 2H), 1.36 (tt, *J* = 6.3, 2.9 Hz, 4H), 0.96 – 0.86 (m, 3H). ^{13}C NMR (126 MHz, chloroform-*d*) δ 149.42, 149.21, 144.61, 141.06, 138.34, 134.82, 129.73, 127.54, 126.15, 125.90, 125.71, 125.29, 124.84, 122.07, 115.65, 47.68, 31.50, 26.84, 26.69, 22.65, 14.06. ESI-HRMS calculated for $C_{40}H_{36}N_3S$ $[M+H]^+$ m/z = 590.2624, found m/z = 590.2599.

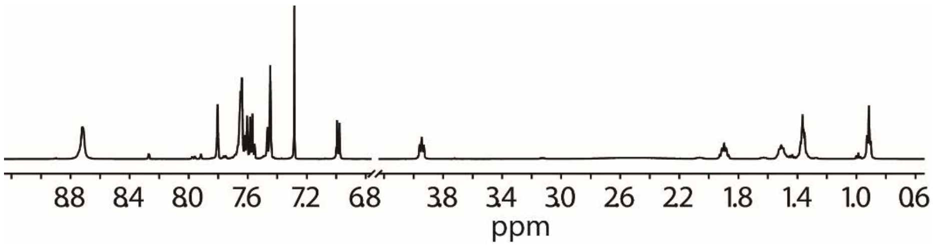

Figure S5. ^{1}H NMR spectrum (500 MHz, chloroform-*d*) of **L**$^{\mathbf{f}}$.

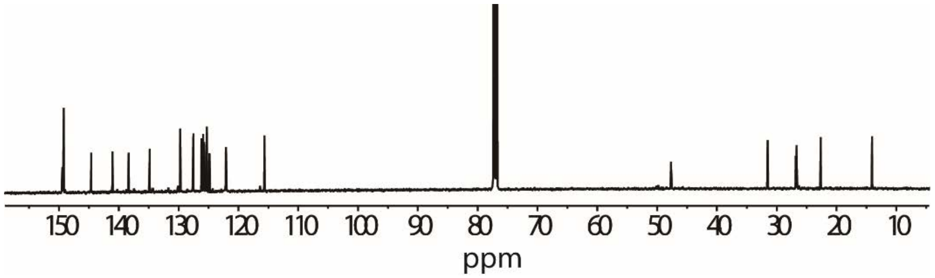

Figure S6. ^{13}C NMR spectrum (126 MHz, chloroform-*d*) of **L**$^{\mathbf{f}}$.

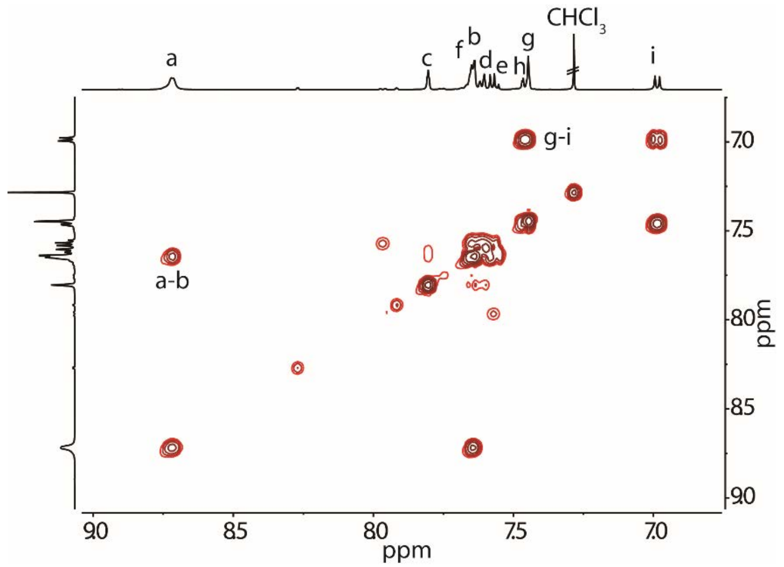

Figure S7. ^{1}H–^{1}H COSY spectrum (500 MHz, chloroform-*d*) of $\mathbf{L^{f}}$ (only showing aromatic region).

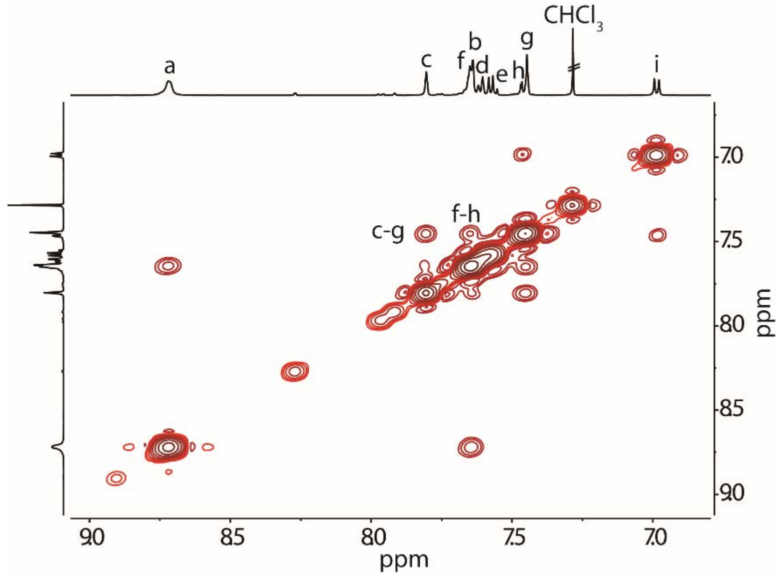

Figure S8. ^{1}H–^{1}H NOESY spectrum (500 MHz, chloroform-*d*) of $\mathbf{L^{f}}$ (only showing aromatic region).

2.2. Cage synthesis

2.2.1 Synthesis of *[Pd$_2$(o-**L**^{a})$_4$](BF$_4$)$_4$* (o-***C***)

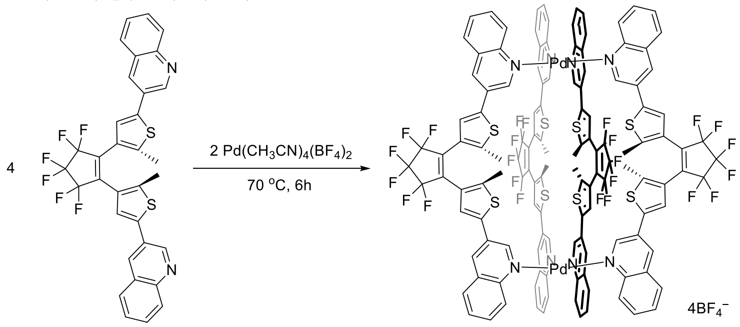

Scheme S4 Synthesis of *o*-**C**.

The open cage compound [Pd$_2$(*o*-**L**^{a})$_4$](BF$_4$)$_4$ (*o*-**C**) was synthesized in quantitative yield by heating a mixture of the ligand *o*-**L**^{a} in CD$_3$CN (4 μmol, 2.5 mg, 866.7 μL) and Pd(CH$_3$CN)$_4$(BF$_4$)$_2$ (2 μmol, 133.3 μL of a 15 mM stock solution in CD$_3$CN) at 70 °C for 6 h to give 1000 μl of a 1 mM solution of *o*-**C**. ^{1}H NMR (500 MHz, acetonitrile-*d*$_3$) δ 9.75 (s, 8H), 9.69 (d, *J* = 8.7 Hz, 8H), 8.90 (s, 8H), 7.98 (d, *J* = 8.2 Hz, 8H), 7.92 (m, 8H), 7.74 (t, *J* = 7.6 Hz, 8H), 7.70 (s, 8H), 2.00 (s, 24H). ESI-HRMS calculated for [Pd$_2$(*o*-**L**^{a})$_4$+BF$_4$]$^{3+}$ *m/z* = 929.7325, found *m/z* = 929.7429.

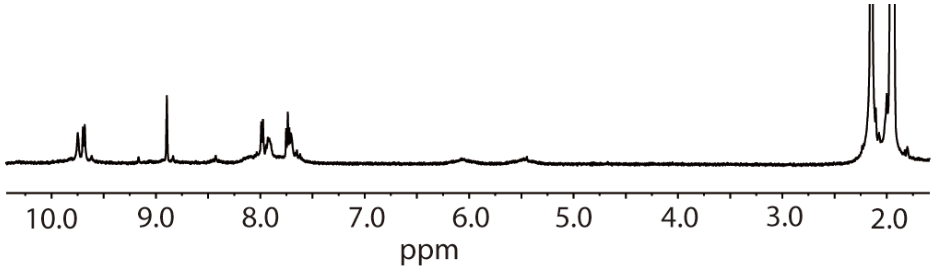

Figure S9. ^{1}H NMR spectrum (500 MHz, acetonitrile-*d*$_3$) of *o*-**C**.

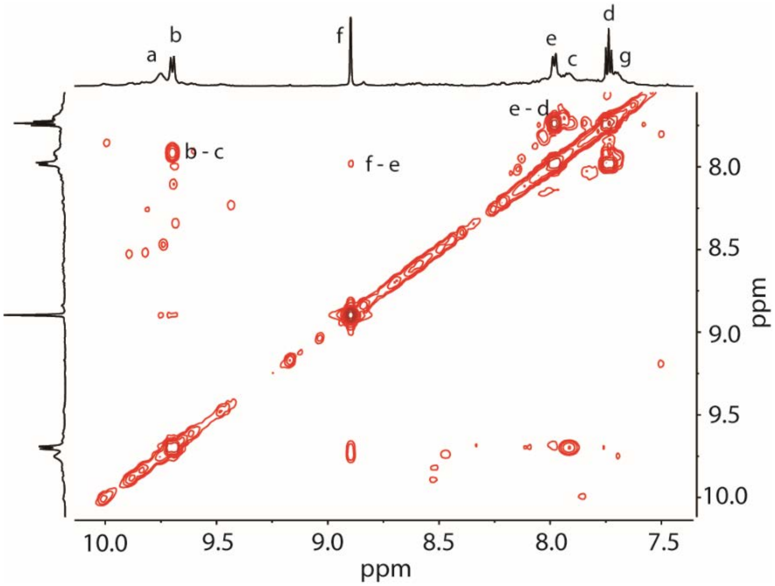

Figure S10. ^{1}H–^{1}H COSY spectrum (600 MHz, acetonitrile-*d*$_3$) of *o*-**C** (only showing aromatic region).

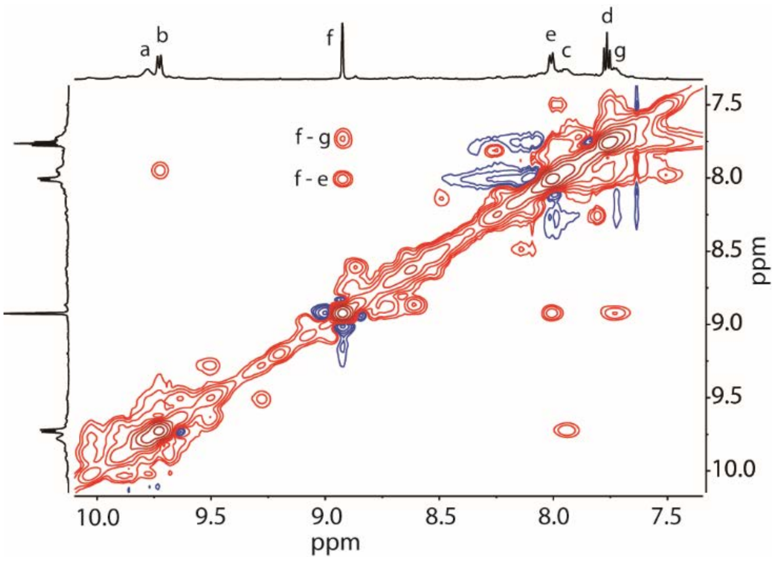

Figure S11. ^{1}H–^{1}H NOESY spectrum (600 MHz, acetonitrile-d_3) of *o*-**C** (only showing aromatic region).

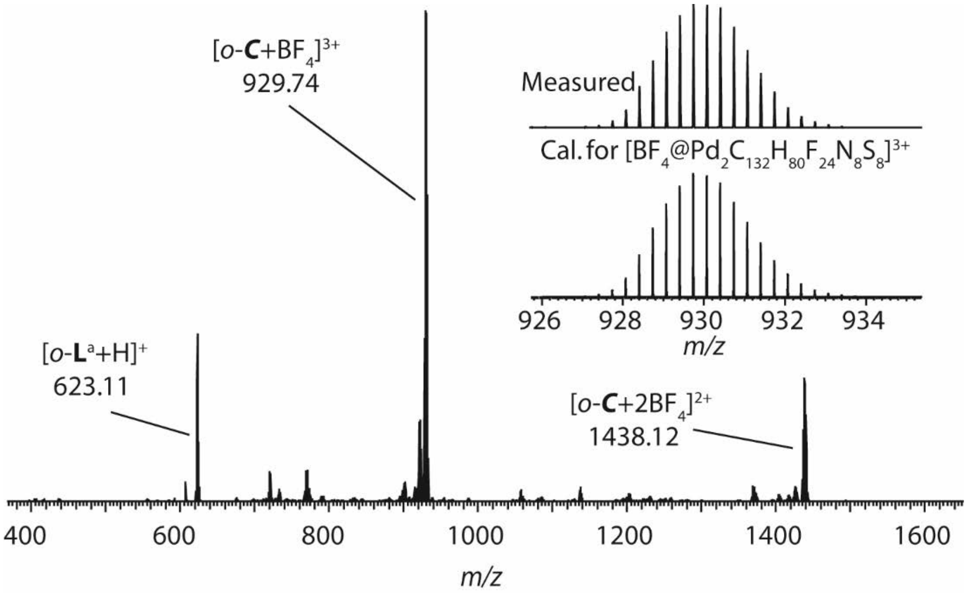

Figure S12. ESI-HRMS spectrum of *o*-**C**.

2.2.2 Synthesis of *[Pd₂(c-**L**ᵃ)₃(Solvent)₂](BF₄)₄* (c-***B***)

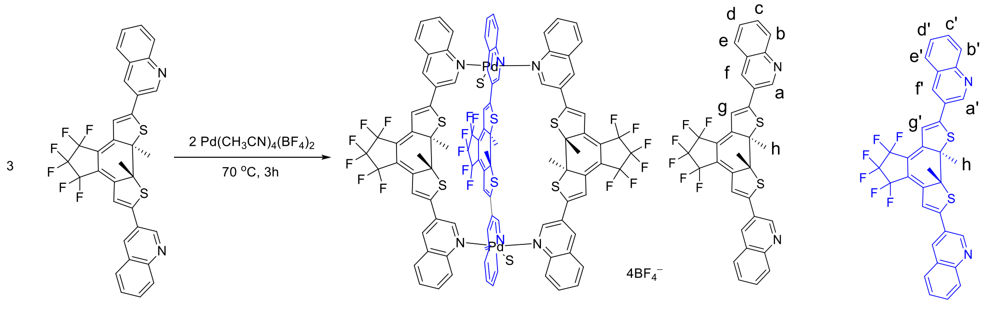

Scheme S5 Synthesis of *c*-**B**, S means coordinated solvents on Pd^{II}.

The closed bowl compound $[Pd_2(c\text{-}\mathbf{L^a})_3(Solvent)_2](BF_4)_4$ (*c*-**B**) was synthesized in quantitative yield by heating a mixture of the ligand *c*-**L**[a] in CD_3CN (3 µmol, 1.9 mg, 866.7 µL) and $[Pd(CH_3CN)_4](BF_4)_2$ (2 µmol, 133.3 µL of a 15 mM stock solution in CD_3CN) at 70 °C for 6 h to give 1000 µl of a 1.0 mM solution of the cage compound *c*-**B**. ^{1}H NMR (700 MHz, acetonitrile-d_3) δ 10.02 – 9.83 (10H), 9.55 – 9.41 (2H), 9.18 (4H), 8.98 (2H), 8.60 – 8.47 (4H), 8.20 – 8.09 (6H), 7.99 (4H), 7.94 – 7.87 (2H), 7.76 – 7.68 (2H), 7.27 – 7.22 (4H), 7.11 (2H), 2.51 – 2.46 (6H), 2.46 – 2.42 (12H). ESI-HRMS calculated for $[Pd_2(c\text{-}\mathbf{L^a})_3+BF_4]^{3+}$ *m/z* = 722.3668, found *m/z* = 722.3737.

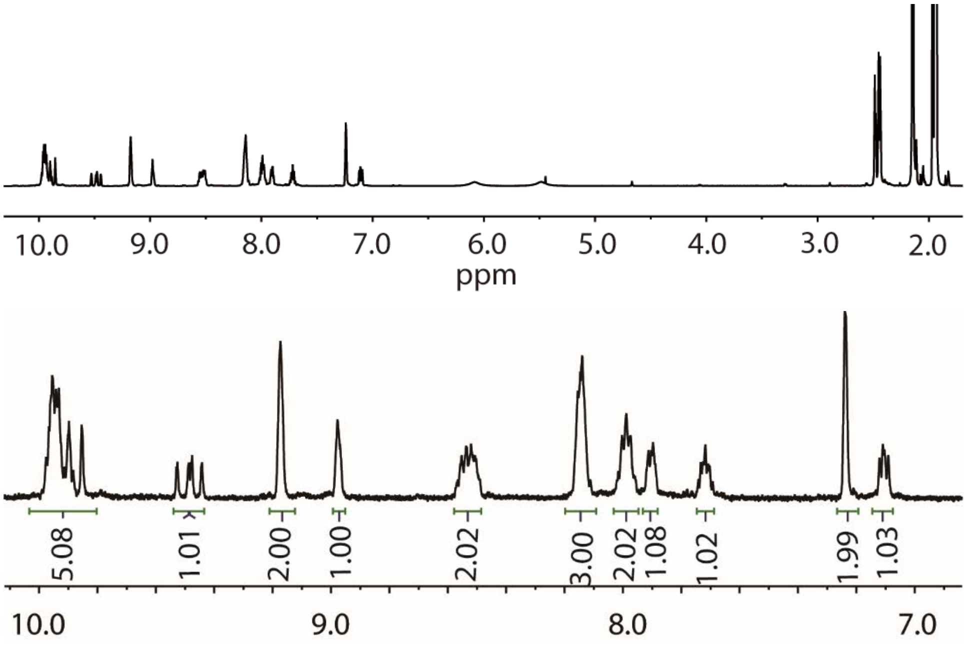

Figure S13. ^{1}H NMR spectrum (700 MHz, acetonitrile-d_3) of *c*-**B**.

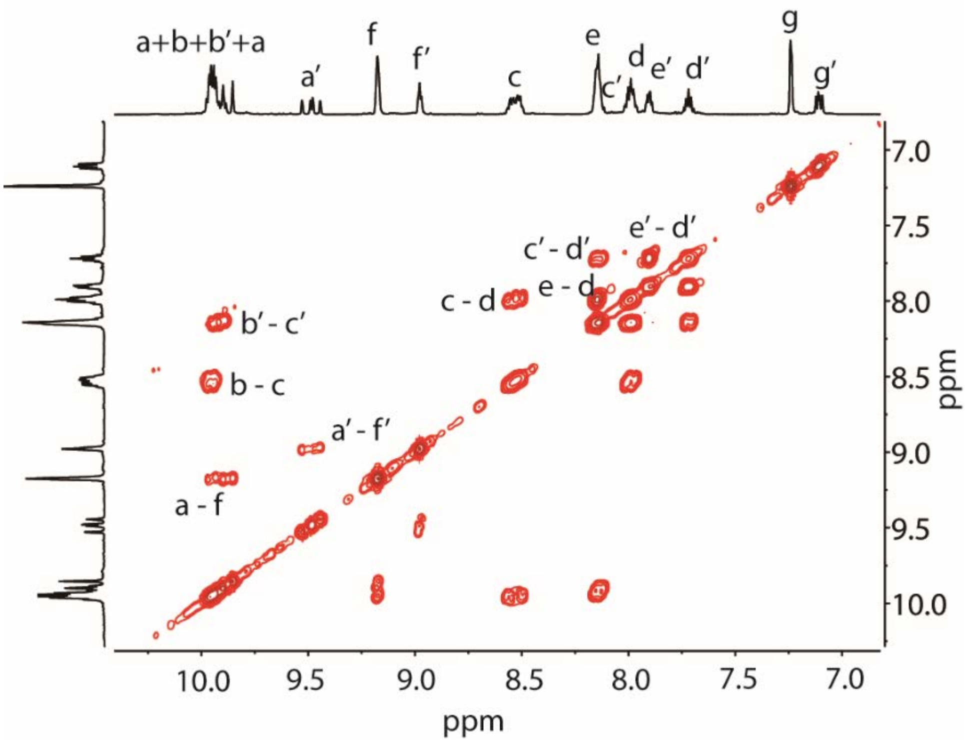

Figure S14. ^{1}H–^{1}H COSY spectrum (700 MHz, acetonitrile-d_3) of *c*-**B** (only showing aromatic region).

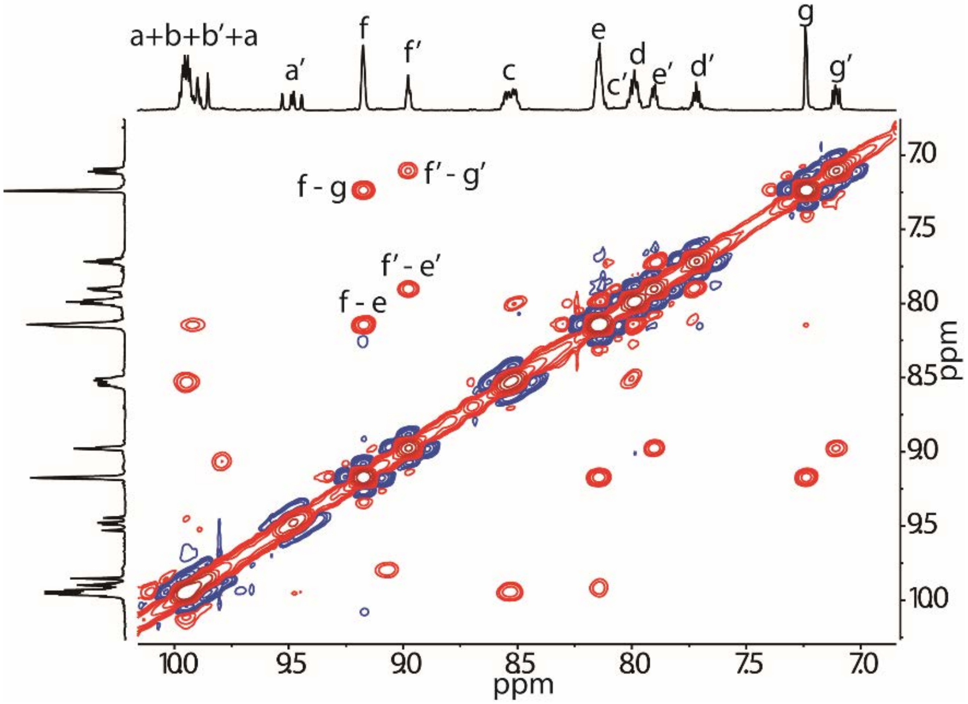

Figure S15. ^{1}H–^{1}H NOESY spectrum (700 MHz, acetonitrile-d_3) of *c*-**B** (only showing aromatic region).

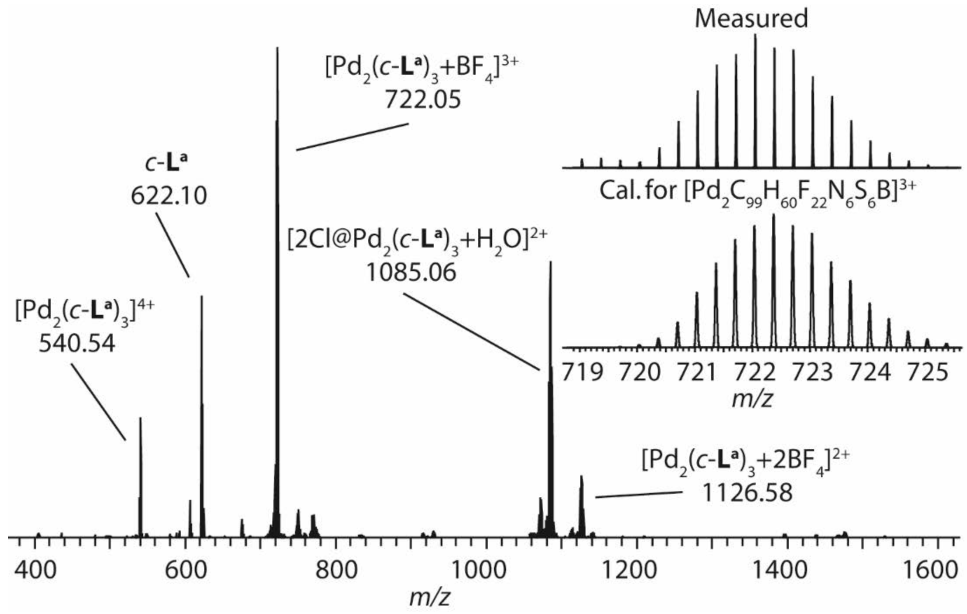

Figure S16. ESI-HRMS spectrum of *c*-**B**.

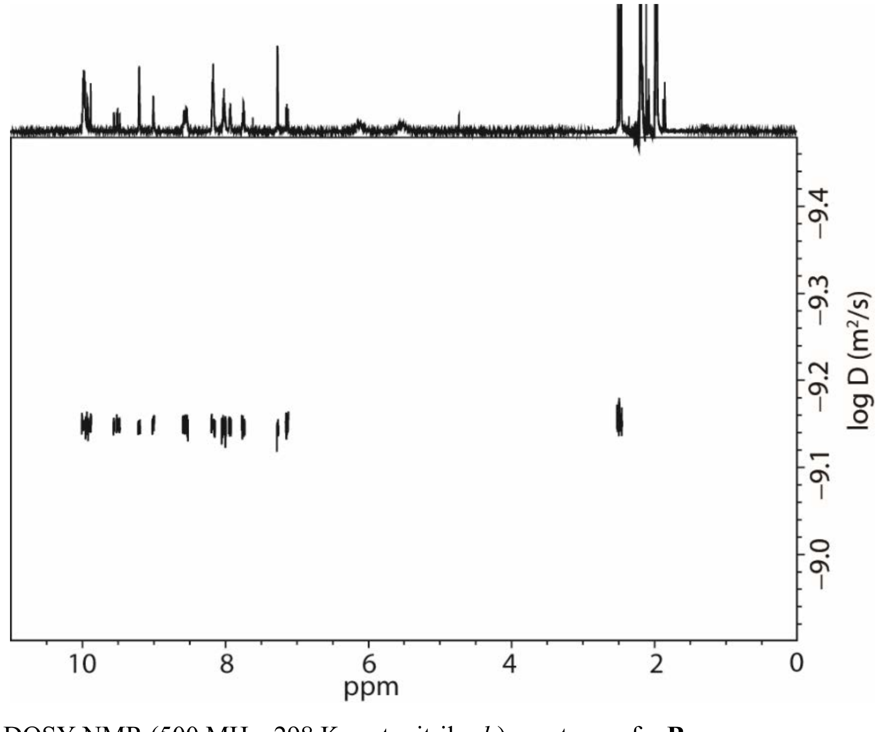

Figure S17. DOSY NMR (500 MHz, 298 K acetonitrile-d_3) spectrum of *c*-**B**.

2.2.3 Synthesis of *[2Cl@Pd$_2$(c-**L^a**)$_3$](BF$_4$)$_2$* (c-***B-Cl***)

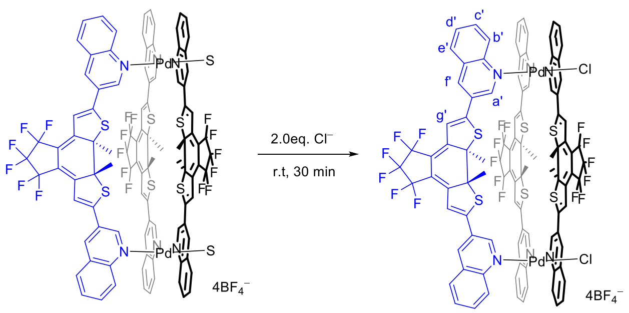

Scheme S6 Synthesis of *c*-**B**-**Cl**.

The closed bowl compound [2Cl@Pd$_2$(*c*-**L^a**)$_3$](BF$_4$)$_2$ (*c*-**B**-**Cl**)was synthesized in quantitative yield by stirring a mixture of *c*-**B** in CD$_3$CN (0.7 µmol, 906.7 µL) and NBu$_4$Cl (1.4 µmol, 99.3 µL of a 15 mM stock solution in CD$_3$CN) at room temperature for 30 minutes in a closed vial to yield 1000 µl of a 0.7 mM solution of *c*-**B**-**Cl**. For a clearer analysis, only the NMR spectra of enantiomerically pure ((*R*)-*c*-**B**-**Cl**) are given. ^{1}H NMR (600 MHz, acetonitrile-*d*$_3$) δ 10.21 (dd, *J* = 8.6, 4.3 Hz, 4H), 10.13 (d, *J* = 8.7 Hz, 2H), 10.10 (s, 2H), 10.02 (s, 2H), 9.71 (s, 2H), 9.09 (s, 4H), 8.91 (s, 2H), 8.44 (dddd, *J* = 10.2, 8.5, 7.0, 1.4 Hz, 4H), 8.10 (m, 4H), 8.06 (m, 2H), 7.91 (m, 4H), 7.87 (m, 2H), 7.66 (m, 2H), 7.20 (s, 4H), 7.08 (s, 2H), 2.48 (s, 6H), 2.44 (s, 6H), 2.43 (s, 6H). ESI-HRMS calculated for [2Cl@Pd$_2$(*c*-**L^a**)$_3$]$^{2+}$ *m/z* = 1075.5195, found *m/z* = 1075.5370.

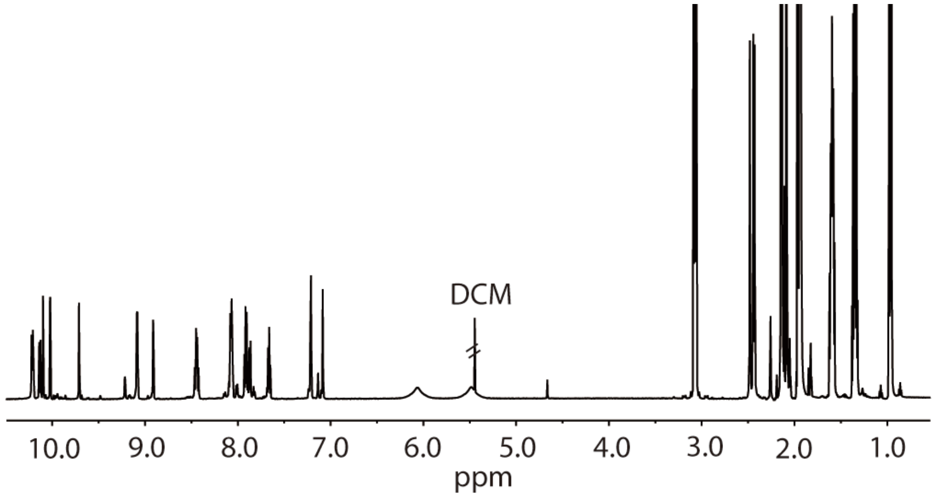

Figure S18. ^{1}H NMR spectrum (600 MHz, acetonitrile-*d*$_3$) of *c*-**B**-**Cl**.

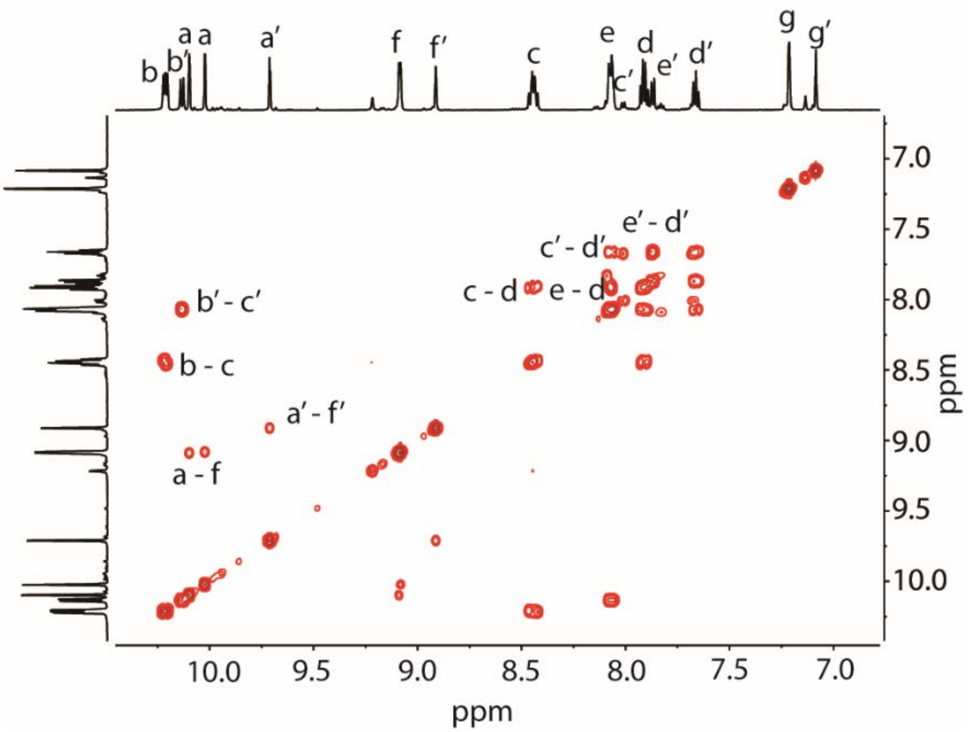

Figure S19. ^{1}H–^{1}H COSY spectrum (600 MHz, acetonitrile-d_3) of (*R*)-*c*-**B-Cl** (only showing aromatic region).

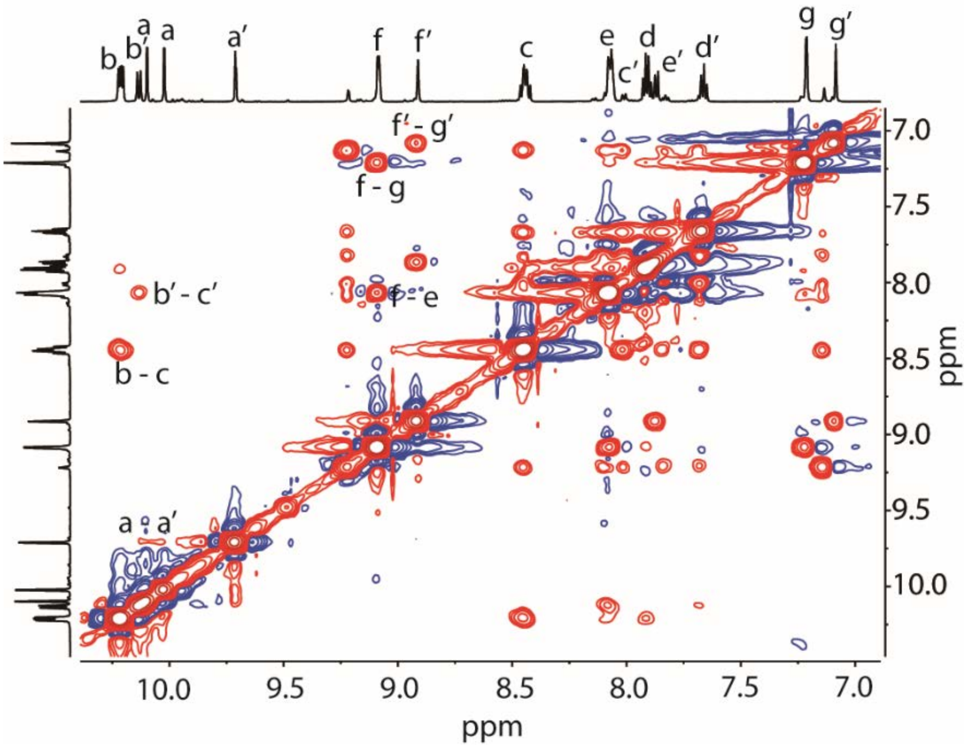

Figure S20. ^{1}H–^{1}H NOESY spectrum (600 MHz, acetonitrile-d_3) of (*R*)-*c*-**B**-Cl (only showing aromatic region).

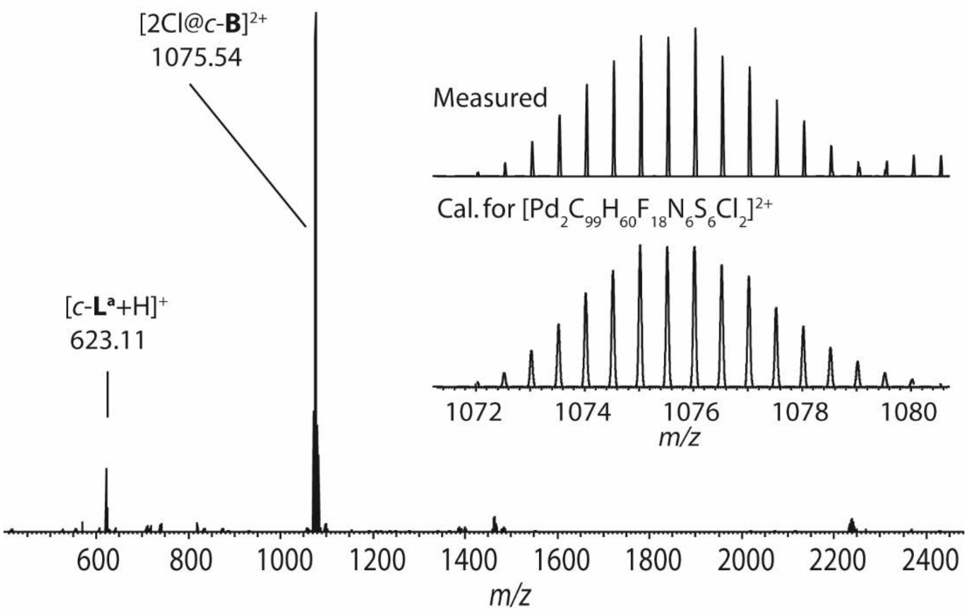

Figure S21. ESI-HRMS spectrum of *c*-**B**-**Cl**.

2.2.4 Synthesis of *[2Br@Pd$_2$(c-**L**^{a})$_3$](BF$_4$)$_2$* (c-***B***-***Br***)

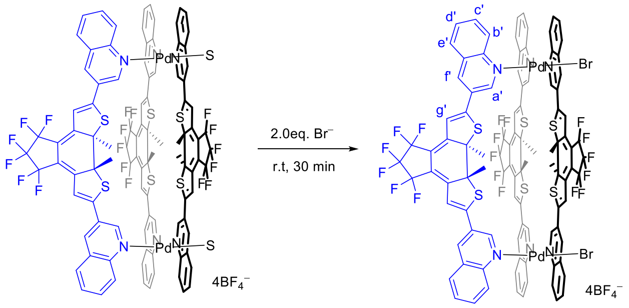

Scheme S7 Synthesis of *c*-**B**-**Br**.

The closed bowl compound [2Br@Pd$_2$(*c*-**L**^{a})$_3$](BF$_4$)$_2$ (*c*-**B**-**Br**) was synthesized in quantitative yield by stirring a mixture of the [Pd$_2$(*c*-**L**^{a})$_3$(CH$_3$CN)$_2$](BF$_4$)$_4$ in CD$_3$CN (0.7 µmol, 906.7 µL) and NBu$_4$Br (1.4 µmol, 99.3 µL of a 15 mM stock solution in CD$_3$CN) at room temperature for 30 minutes in a closed vial to yield 1000 µl of a 0.7 mM solution of *c*-**B**-**Br**. For clearer analysis, only NMR spectra of enantiomerically pure ((*R*)-*c*-**B**-**Br**) are given. ^{1}H NMR (600 MHz, acetonitrile-*d*$_3$) δ 10.19 (m, 4H), 10.12 (s, 2H), 10.10 (d, *J* = 8.5, 2H), 10.05 (s, 2H), 9.67 (s, 2H), 9.08 (s, 4H), 8.91 (s, 2H), 8.44 (dddd, *J* = 10.2, 8.5, 7.0, 1.4 Hz, 4H), 8.06 (m, 4H), 8.03 (m, 2H), 7.91 (m, 4H), 7.86 (m, 2H), 7.65 (ddd, *J* = 8.1, 7.2, 1.0 Hz, 2H), 7.22 (s, 4H), 7.08 (s, 2H), 2.47 (s, 6H), 2.45 (s, 6H), 2.43 (s, 6H). ESI-HRMS calculated for [2Br@Pd$_2$(*c*-**L**^{a})$_3$]$^{2+}$ *m/z* = 1119.9663, found *m/z* = 1119.9854.

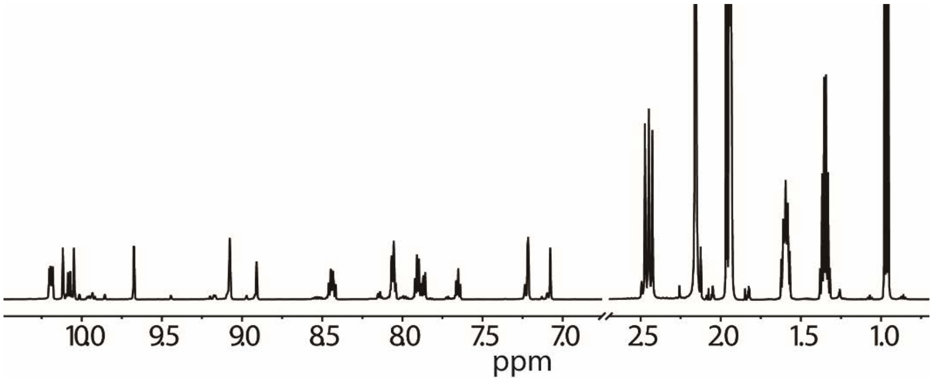

Figure S22. ^{1}H NMR spectrum (600 MHz, acetonitrile-d_3) of (*R*)-*c*-**B-Br**.

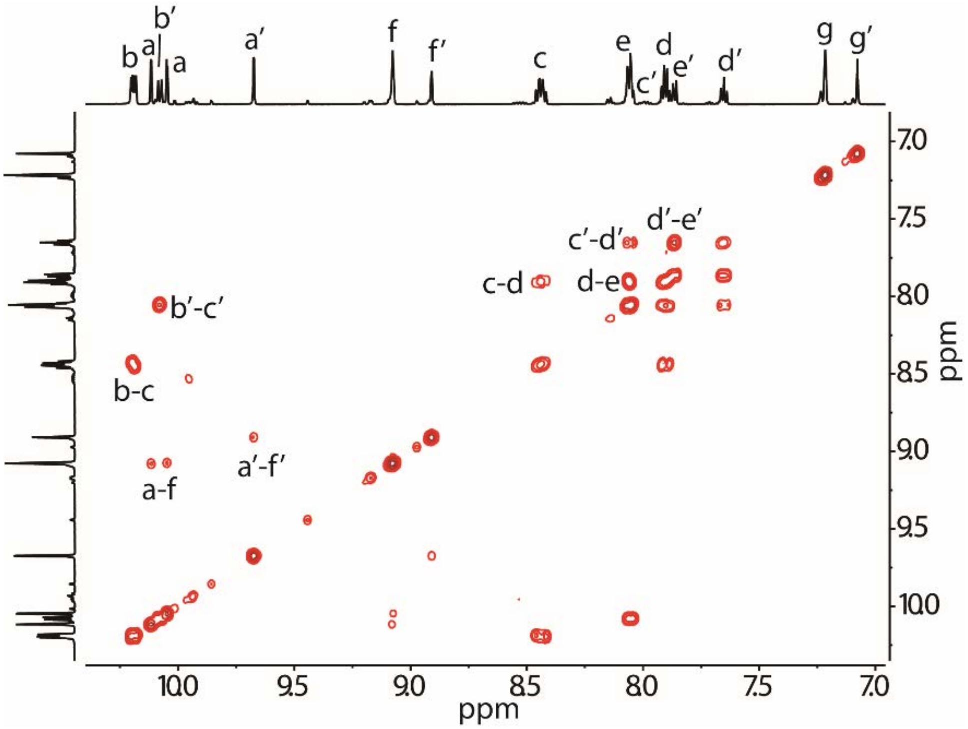

Figure S23. ^{1}H–^{1}H COSY spectrum (600 MHz, acetonitrile-d_3) of (*R*)-*c*-**B**-Br (only showing aromatic region).

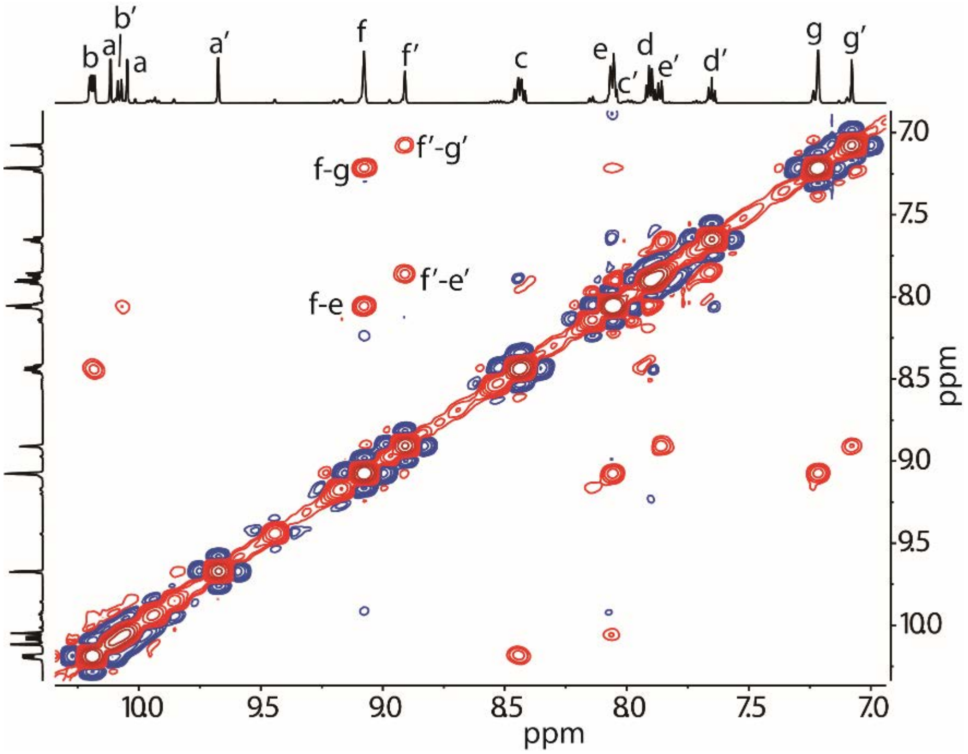

Figure S24. ^{1}H–^{1}H NOESY spectrum (600 MHz, acetonitrile-d_3) of (*R*)-*c*-**B**-Br (only showing aromatic region).

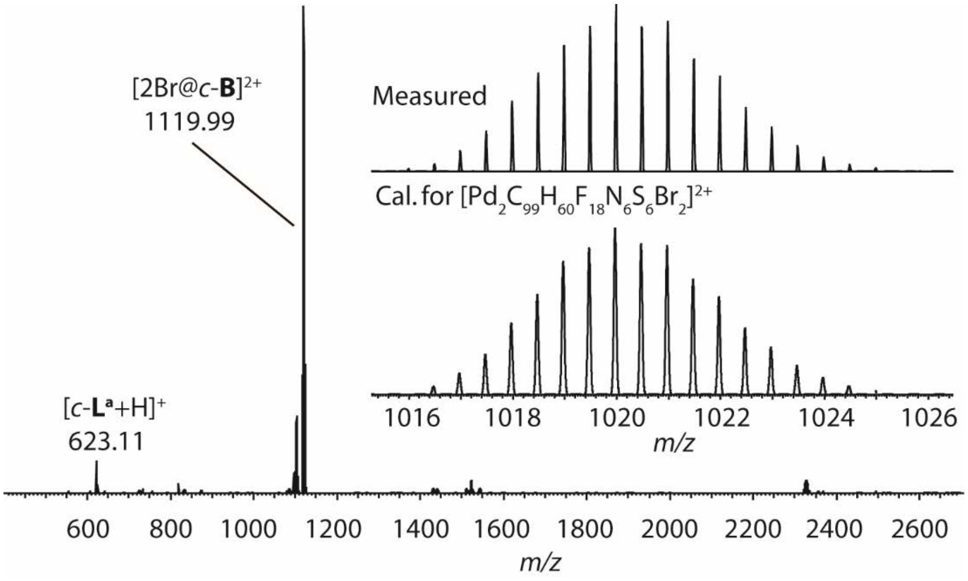

Figure S25. ESI-HRMS spectrum of [2Br@Pd_2(*c*-**L**^{a})$_3$](BF_4)$_2$.

2.3. Heteroleptic cage synthesis

2.3.1 Synthesis of *[Pd₂(c-**L**ᵃ)₃(o-**L**ᶜ)₃](BF₄)₄* (c-***B*-*L*ᶜ**)

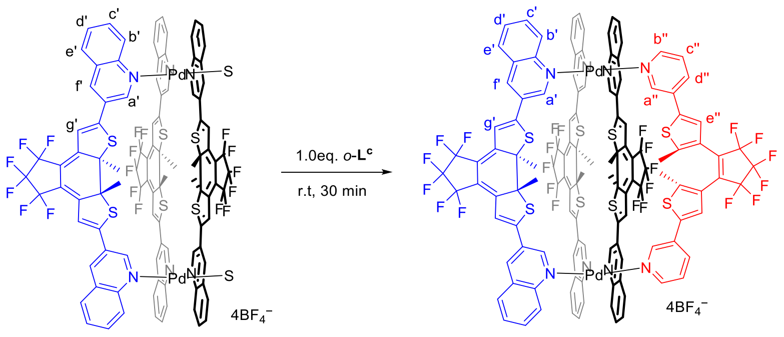

Scheme S8 Synthesis of *c*-**B**-**L**^{c}.

The heteroleptic cage compound [Pd$_2$(*c*-**L**^{a})$_3$(*o*-**L**^{c})] (*c*-**B**-**L**^{c})was synthesized in quantitative yield by stirring a mixture of the [Pd$_2$(*c*-**L**^{a})$_3$] in CD_3CN (0.7 µmol, 976.67 µL) and *o*-L^{4} (0.7 µmol, 23.33 µL of a 30 mM stock solution in CD_3CN) at room temperature for 30 minutes in a closed vial to yield 1000 µl of a 0.7 mM solution of *c*-**B**-**L**^{c}. For clearer analysis, only NMR spectra of enantiomerically pure ((*R*)-*c*-**B**-**L**^{c}) are given. ^{1}H NMR (600 MHz, acetonitrile-*d*$_3$) 10.38 (b, 2H), 10.12 (b, 2H), 9.92 – 9.75 (m, 10H), δ 9.13 (s, 2H), 9.11 (s, 2H), 8.99 (s, 2H), 8.86 (b, 2H), 8.65 – 8.54 (m, 4H), 8.19 (dt, *J* = 8.3, 1.5 Hz, 2H), 8.16 – 8.11 (m, 4H), 8.08 (d, *J* = 7.8 Hz, 2H), 8.00 – 7.93 (m, 4H), 7.92 (dd, *J* = 8.2, 1.4 Hz, 2H), 7.72 (ddd, *J* = 8.1, 7.2, 0.9 Hz, 2H), 7.50 (s, 2H), 7.47 (dd, *J* = 8.0, 5.9 Hz, 2H), 7.20 (s, 2H), 7.14 (s, 4H), 2.49 (s, 6H), 2.43 (s, 6H), 2.39 (s, 6H), 1.87 (s, 6H).

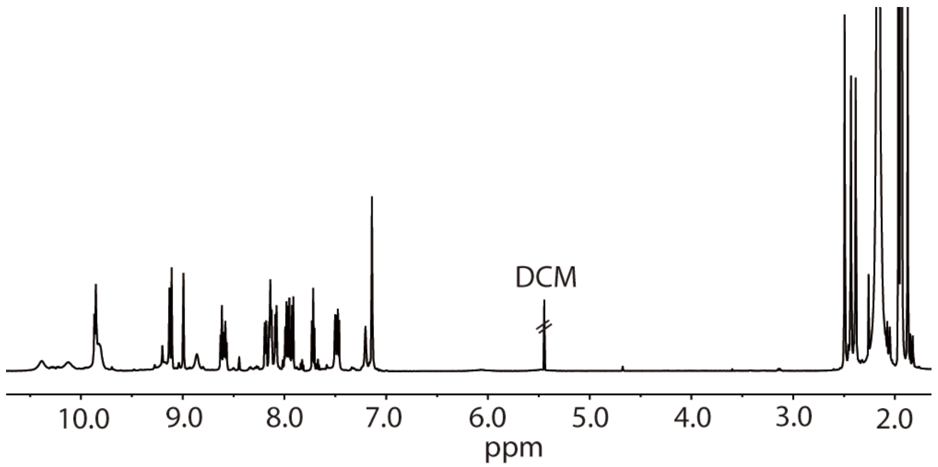

Figure S26. ^{1}H NMR spectrum (600 MHz, acetonitrile-*d*$_3$) of (*R*)-*c*-**B**-**L**^{c}.

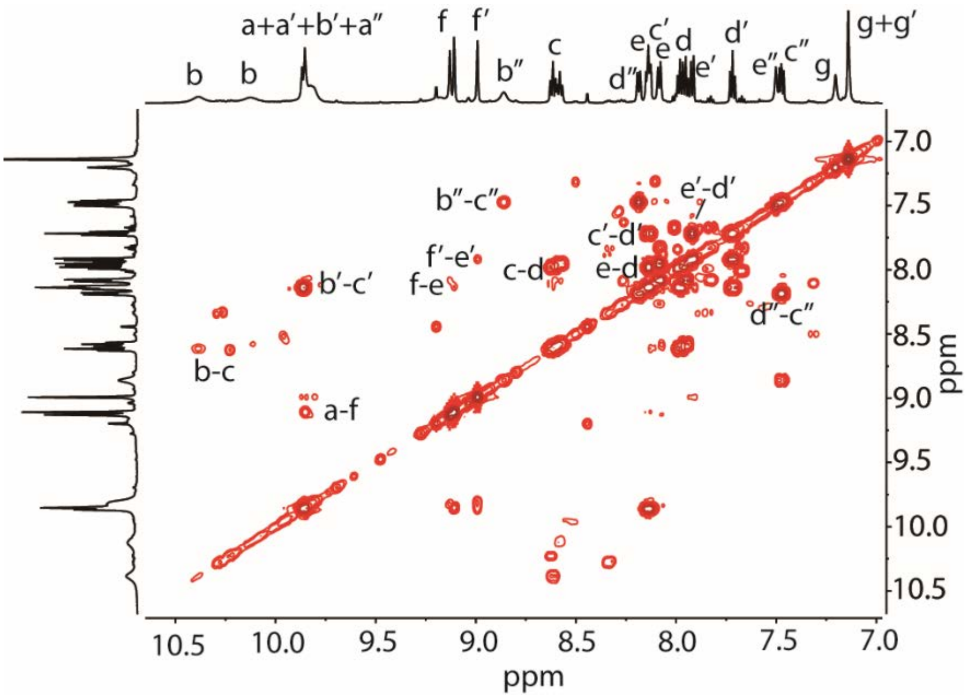

Figure S27. ^1H–^1H COSY spectrum (600 MHz, acetonitrile-d_3) of (*R*)-*c*-**B**-**L**^{c} (only showing aromatic region).

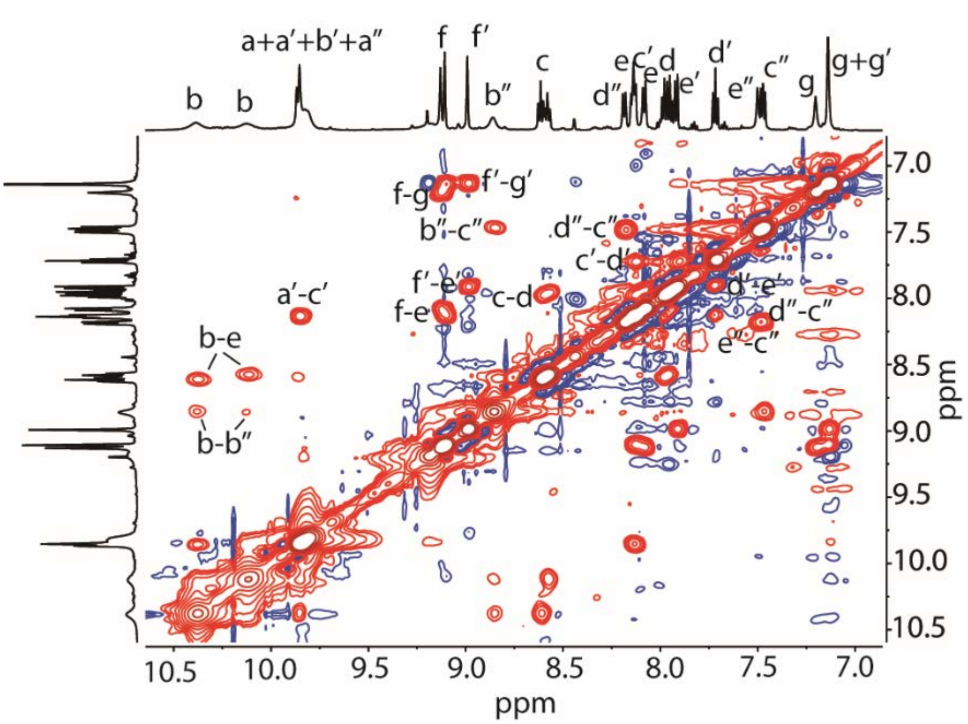

Figure S28. ^1H–^1H NOESY spectrum (600 MHz, acetonitrile-d_3) of (*R*)-*c*-**B**-**L**^{c} (only showing aromatic region).

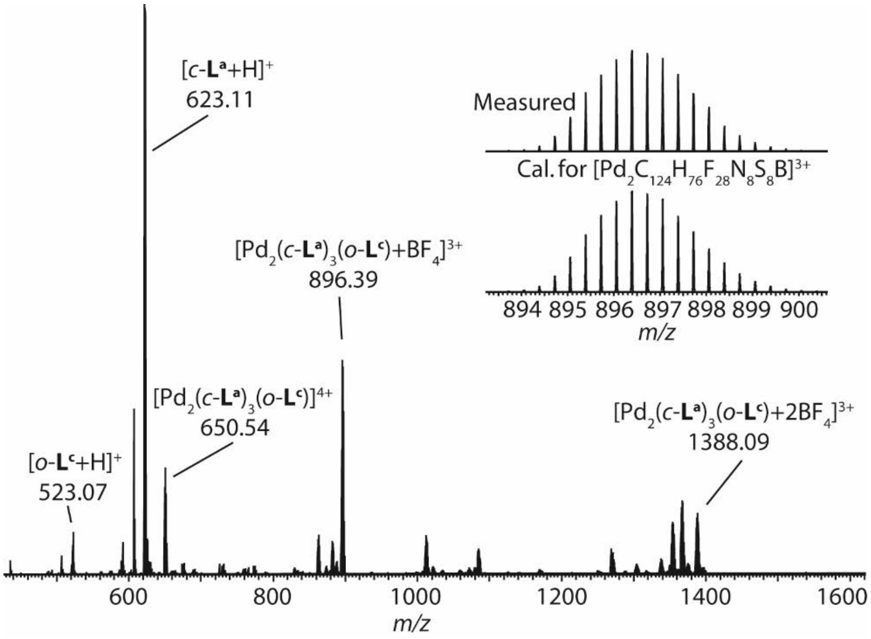

Figure S29. ESI-HRMS spectrum of *c*-**B**-**L**^{c}.

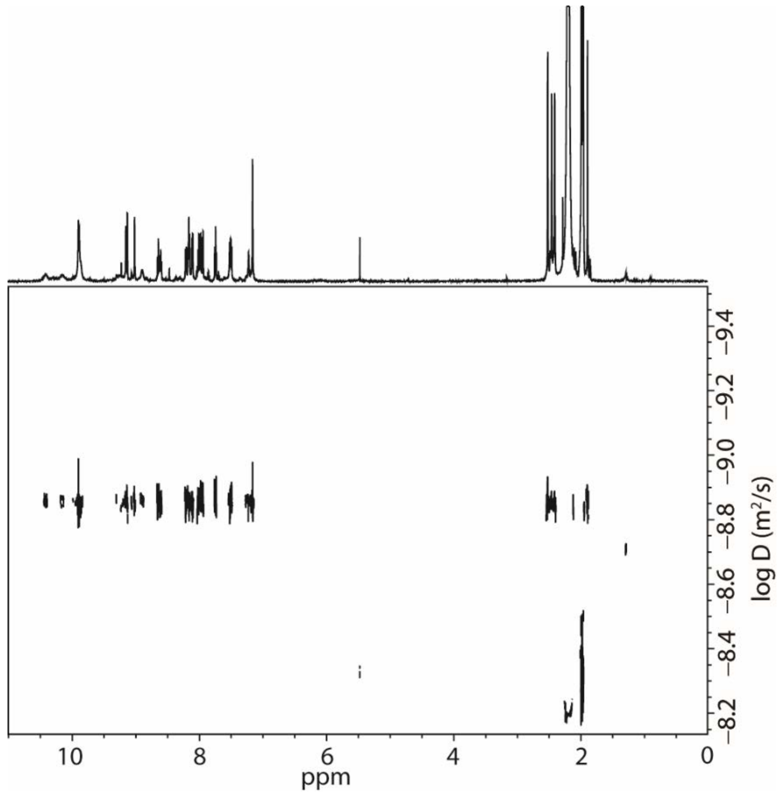

Figure S30. DOSY NMR (500 MHz, 298 K acetonitrile-d_3) spectrum of (*R*)-*c*-**B**-**L**^{c}.

2.3.2 Synthesis of *[Pd_2(c-**L^a**)$_3$**L^d**](BF_4)$_4$* (c-***B*-*L^d***)

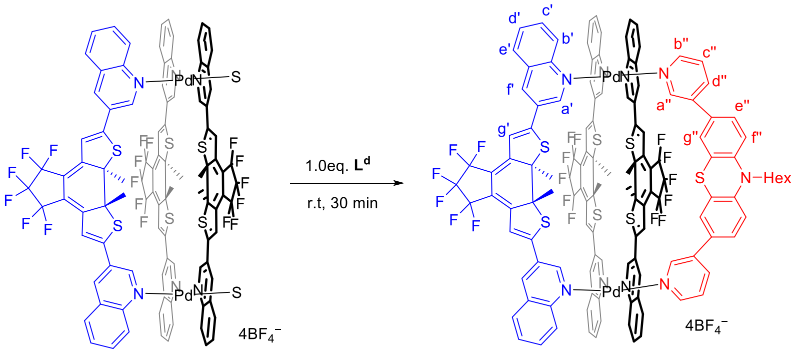

Scheme S9 Synthesis of *c*-**B**-**L^d**.

The heteroleptic cage compound [Pd_2(*c*-**L^a**)$_3$**L^d**](BF_4)$_4$ (*c*-**B**-**L^d**) was synthesized in quantitative yield by stirring a mixture of the [Pd_2(*c*-**L^a**)$_3$(CH_3CN)$_2$](BF_4)$_4$ in CD_3CN (0.7 µmol, 976.7 µL) and **L^d** (0.7 µmol, 23.3 µL of a 4 mM stock solution in CD_3CN) at room temperature for 30 minutes in a closed vial to yield 1000 µl of a 0.7 mM solution of *c*-**B**-**L^d**. For clearer analysis, only NMR spectra of enantiomerically pure ((*R*)-*c*-**B**-**L^d**) are given. ^{1}H NMR (600 MHz, acetonitrile-*d*$_3$) δ 10.20 (d, *J* = 8.6 Hz, 2H), 10.14 (d, *J* = 8.6 Hz, 2H), 9.99 (d, *J* = 8.7 Hz, 2H), 9.79 (s, 2H), 9.76 (s, 2H), 9.55 (s, 2H), 9.28 (s, 2H), 9.09 (d, *J* = 5.9 Hz, 2H), 9.08 (s, 2H), 9.06 (s, 2H), 9.00 (s,2H), 8.53 (m, *J* = 8.6, 7.1, 1.4 Hz, 4H), 8.16 (d, 2H), 8.14 (d, 2H), 8.10 (d, *J* = 8.4 Hz, 4H), 8.08 (d, *J* = 8.7 Hz, 2H), 7.96 (m, 4H), 7.94 (m, 2H),7.5 (m, 2H), 7.64 (s, 2H), 7.51 (dd, *J* = 8.1, 5.9 Hz, 2H), 7.48 (dd, *J* = 8.6, 2.3 Hz, 2H), 7.14 (s, 2H), 7.13 (s, 4H), 7.06 (d, *J* = 8.5 Hz, 2H), 3.91 (t, *J* = 6.9 Hz, 2H), 2.50 (s, 6H), 2.46 (s, 6H), 2.43 (s, 6H), 1.68 (p, *J* = 7.2 Hz, 2H), 1.35 (t, *J* = 7.4 Hz, 2H), 1.23 – 1.16 (m, 4H), 0.82 – 0.73 (m, 3H). ESI-HRMS calculated for [Pd_2(*c*-**L^a**)$_3$(**L^d**)]$^{4+}$ *m/z* = 629.5732, found *m/z* = 629.5746.

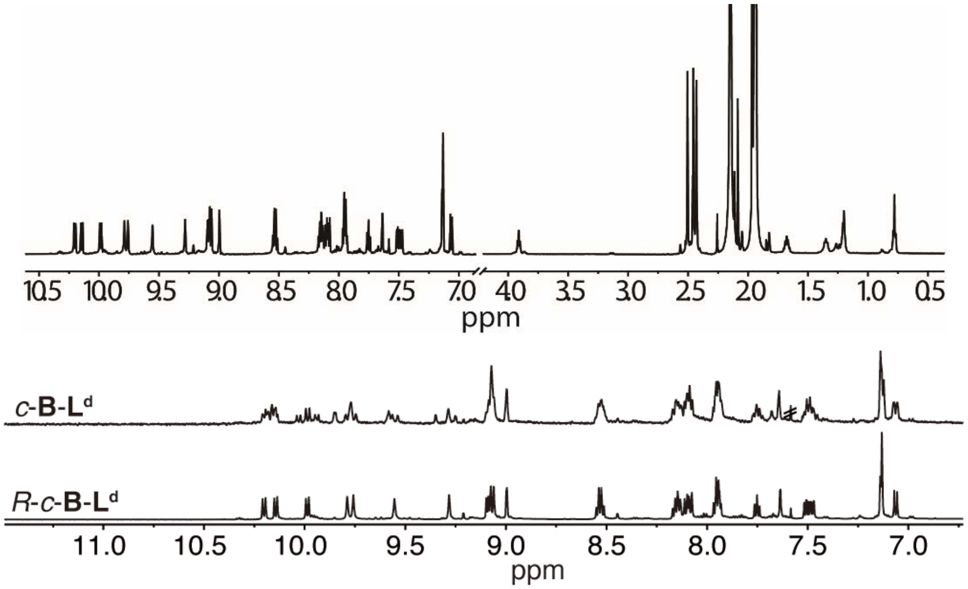

Figure S31. ^{1}H NMR spectrum (600 MHz, acetonitrile-*d*$_3$) of *R*-*c*-**B**-**L^d** and *c*-**B**-**L^d**.

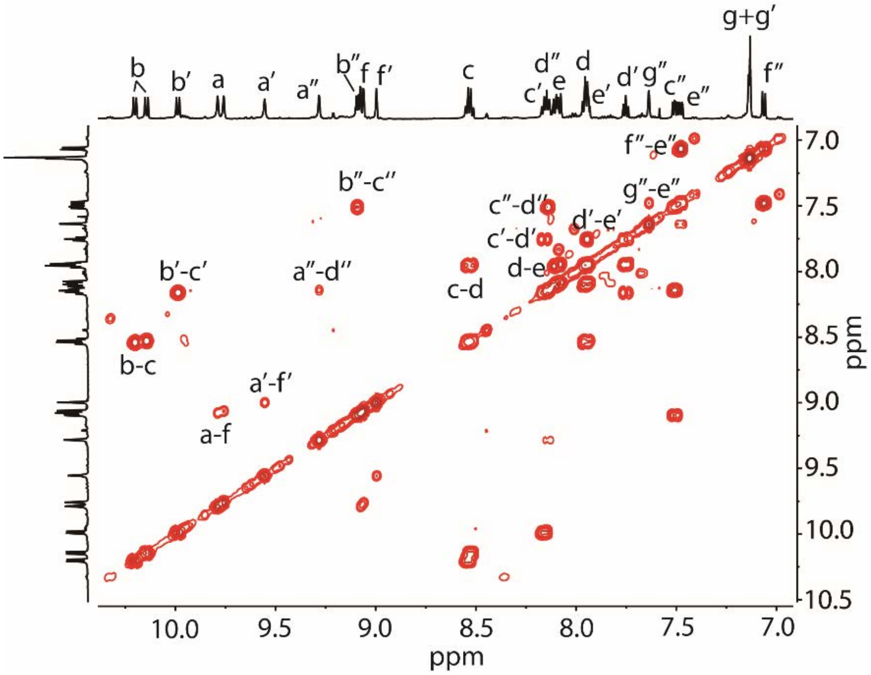

Figure S32. ^{1}H–^{1}H COSY spectrum (600 MHz, acetonitrile-d_3) of (*R*)-*c*-**B-L^{d}** (only showing aromatic region).

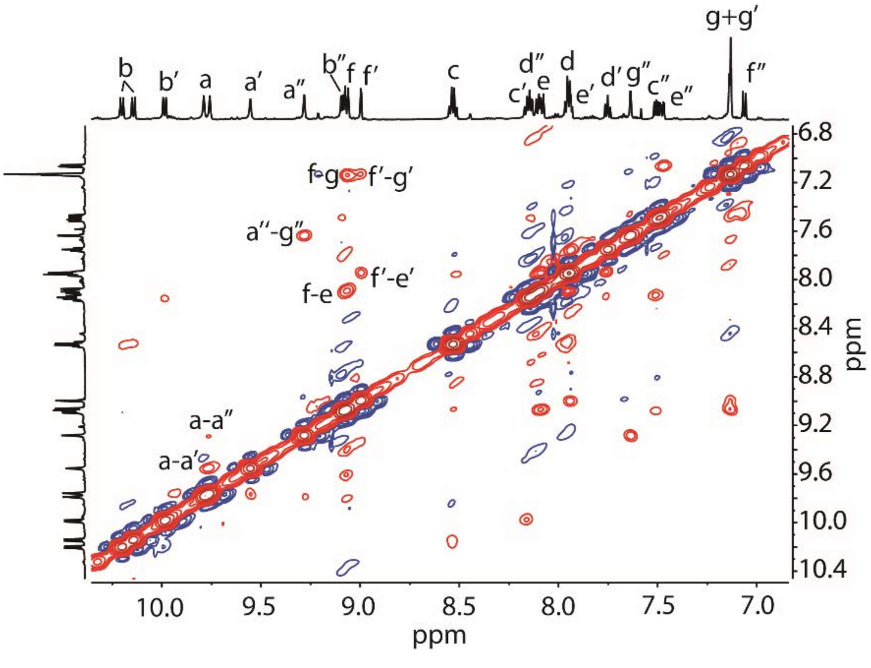

Figure S33. ^{1}H–^{1}H NOESY spectrum (600 MHz, acetonitrile-d_3) of (*R*)-*c*-**B-L^{d}** (only showing aromatic region).

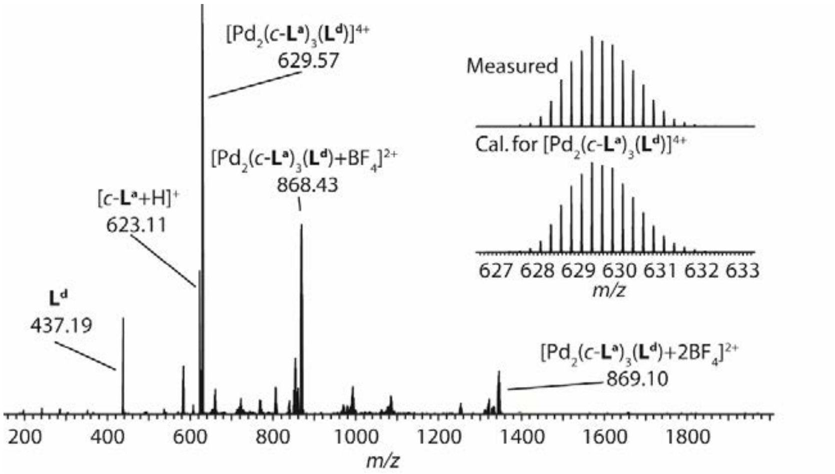

Figure S34. ESI-HRMS spectrum of *c*-**B**-**L**$^{\mathbf{d}}$.

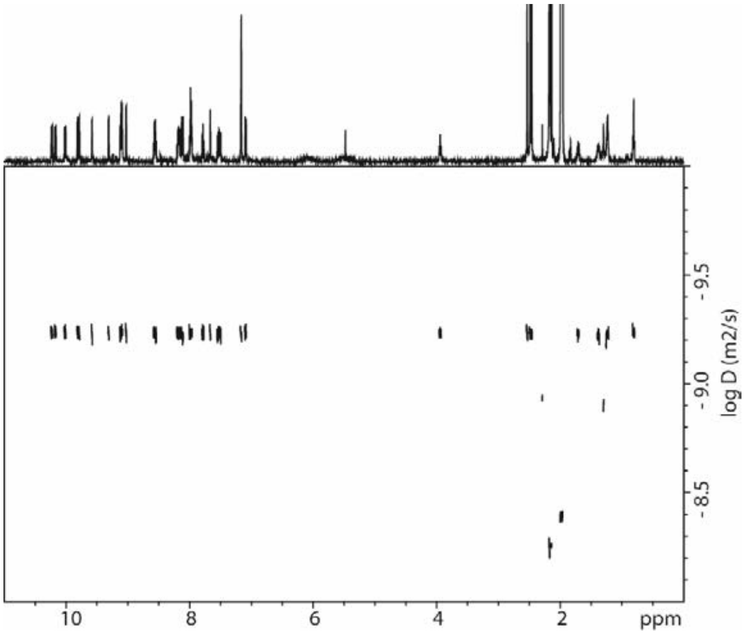

Figure S35. DOSY NMR (500 MHz, 298 K acetonitrile-d_3) spectrum of (*R*)-*c*-**B**-**L**$^{\mathbf{d}}$.

*2.3.3 Synthesis of [Pd$_2$(c-**L**^{a})$_3$(o-**L**^{e})](BF$_4$)$_4$ (c-B-**L**^{e})*

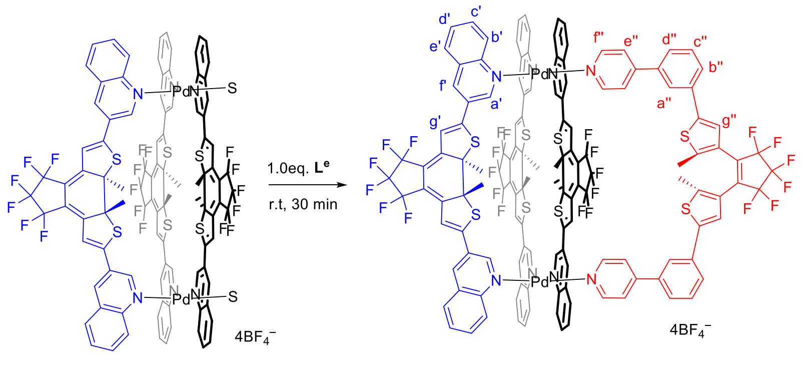

Scheme S10 Synthesis of *c*-**B**-**L**$^{\mathbf{e}}$.

The heteroleptic cage compound [Pd_2(c-$\mathbf{L^a}$)$_3$(o-$\mathbf{L^e}$)]$(BF_4)_4$ (c-**B**-$\mathbf{L^e}$) was synthesized in quantitative yield by stirring a mixture of the [Pd_2(c-$\mathbf{L^a}$)$_3$$(CH_3CN)_2$]$(BF_4)_4$ in CD_3CN (0.7 µmol, 976.7 µL) and o-$\mathbf{L^e}$ (0.7 µmol, 23.3 µL of a 4 mM stock solution in CD_3CN) at room temperature for 30 minutes in a closed vial to yield 1000 µl of a 0.7 mM solution of c-**B**-$\mathbf{L^e}$. For clearer analysis, only NMR spectra of enantiomerically pure ((R)-c-**B**-$\mathbf{L^e}$) are given. ^{1}H NMR (600 MHz, acetonitrile-d_3) δ 10.21 (s, 2H), 10.16 (d, J = 8.6 Hz, 2H), 10.14 (s, 2H), 10.12 (d, J = 8.7 Hz, 2H), 9.99 (d, J = 8.9 Hz, 1H), 9.73 (s, 2H), 9.12 (s, 4H), 8.94 (s, 2H), 8.61 (dd, 4H), 8.50 (dddd, J = 8.6, 7.2, 5.9, 1.4 Hz, 4H), 8.13 (m, 4H), 8.11 (m, 2H), 7.95 (dddd, J = 8.1, 7.1, 5.0, 0.9 Hz, 4H), 7.90 (m, 2H), 7.81 (ddd, J = 7.6, 1.8, 1.0 Hz, 2H), 7.72 (dd, J = 5.0, 3.4 Hz, 4H), 7.70 (d, J = 6.1 Hz, 2H), 7.63 (ddd, J = 8.0, 1.8, 1.0 Hz, 2H), 7.58 (s, 2H), 7.55 (t, J = 7.8 Hz, 2H), 7.35 (s, 2H), 7.16 (s, 2H), 7.14 (s, 2H),7.08 (s, 2H), 2.50 (s, 6H), 2.45 (s, 6H), 2.41 (s, 6H), 1.83 (s, 6H). ESI-HRMS calculated for [Pd_2(c-$\mathbf{L^a}$)$_3$(o-$\mathbf{L^e}$)+BF_4]$^{3+}$ m/z = 947.0763, found m/z = 947.0816.

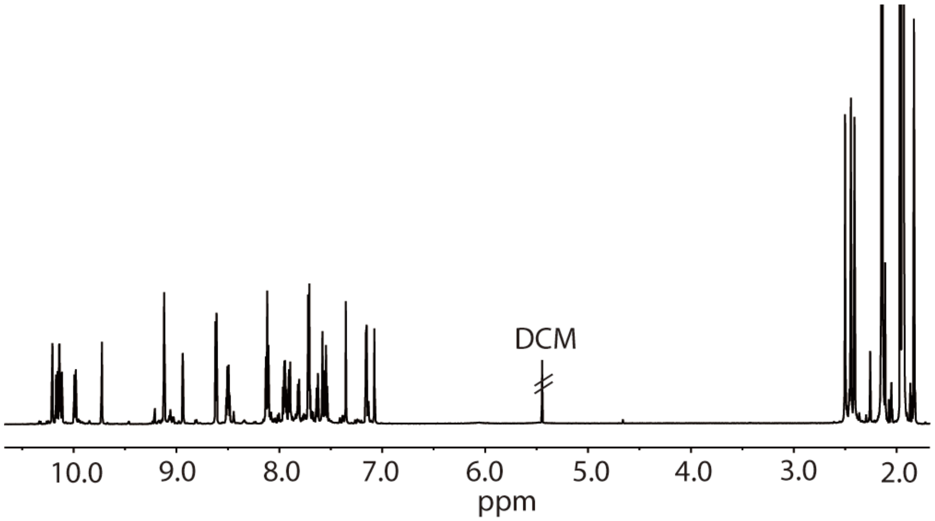

Figure S36. ^{1}H NMR spectrum (600 MHz, acetonitrile-d_3) of (R)-c-**B**-$\mathbf{L^e}$.

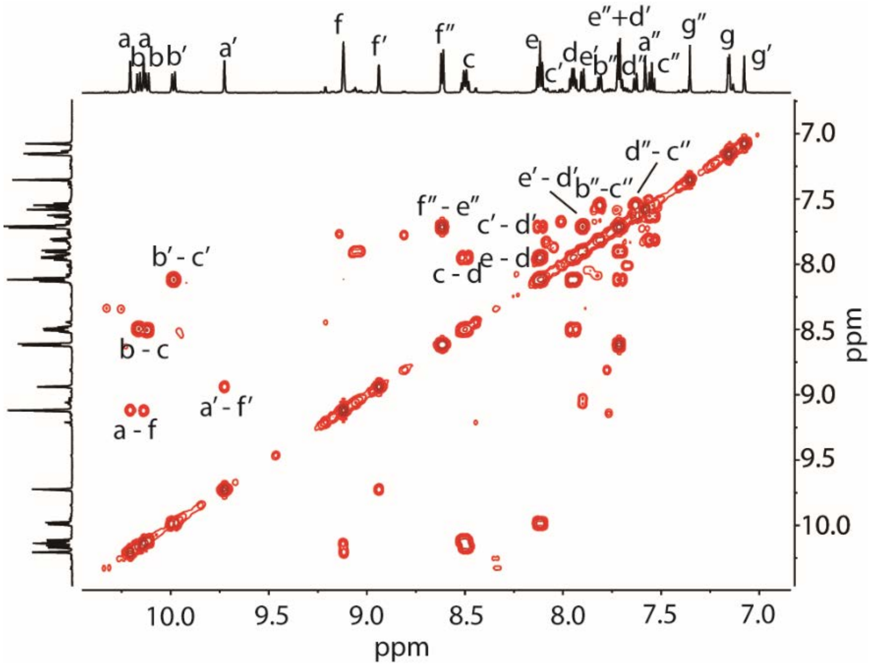

Figure S37. ^{1}H–^{1}H COSY spectrum (600 MHz, acetonitrile-d_3) of (*R*)-*c*-**B-L**^{e} (only showing aromatic region).

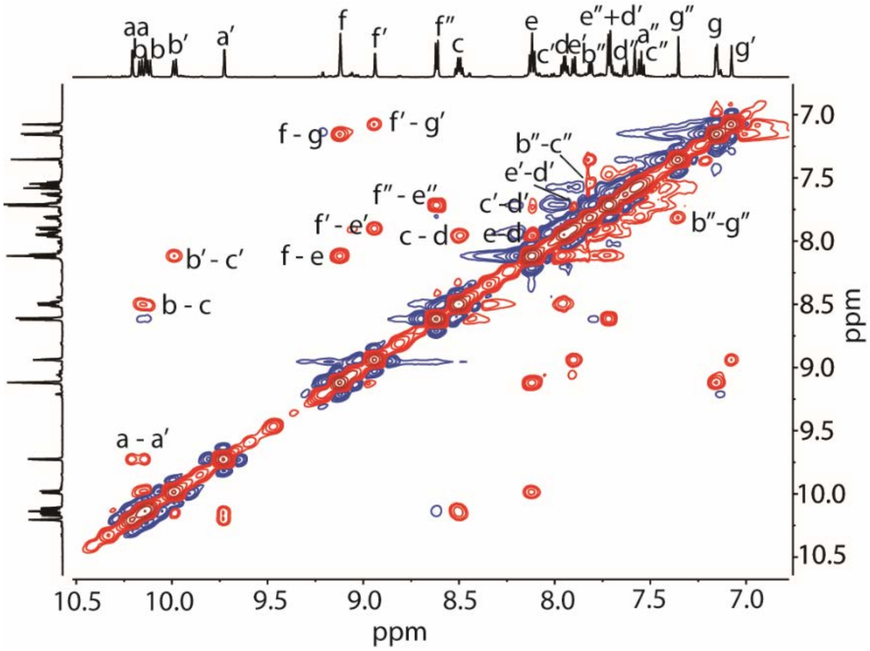

Figure S38. ^{1}H–^{1}H NOESY spectrum (600 MHz, acetonitrile-d_3) of (*R*)-*c*-**B-L**^{e} (only showing aromatic region).

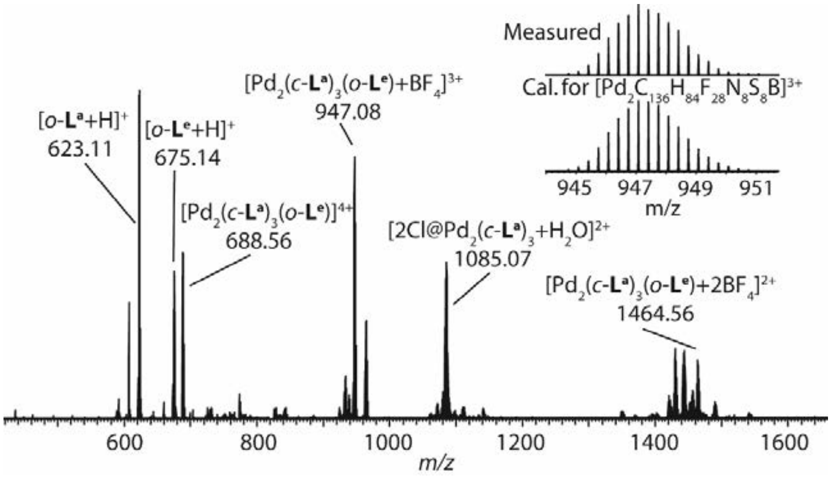

Figure S39. ESI-HRMS spectrum of *c*-**B**-**L**^{e}.

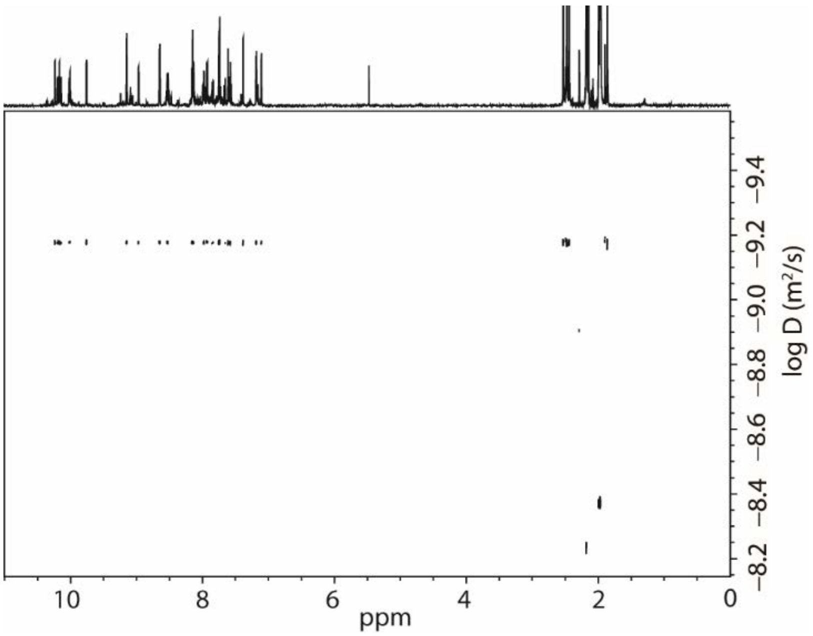

Figure S40. DOSY NMR (500 MHz, 298 K acetonitrile-d_3) spectrum of (*R*)-*c*-**B**-**L**^{e}.

2.3.4 Synthesis of *[Pd2(c-**L**^{a})3**L**^{f}](BF4)4* (c-***B***-***L**^{f}*)

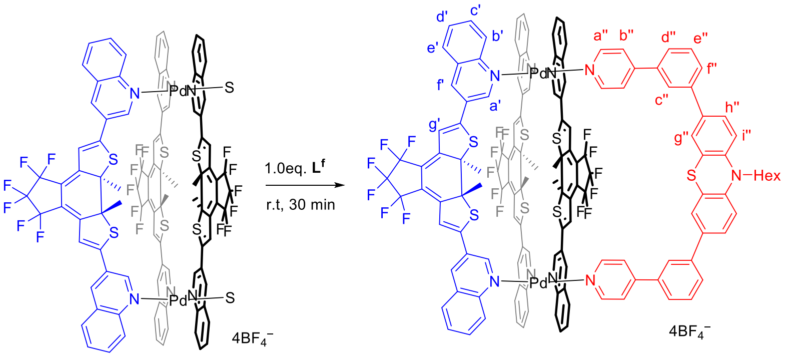

Scheme S11 Synthesis of *c*-**B**-**L**^{f}.

The heteroleptic cage compound $[Pd_2(c\text{-}\mathbf{L^a})_3\mathbf{L^f}](BF_4)_4$ (c-**B**-$\mathbf{L^f}$) was synthesized in quantitative yield by stirring a mixture of the $[Pd_2(c\text{-}\mathbf{L^a})_3(CH_3CN)_2](BF_4)_4$ in CD_3CN (0.7 µmol, 976.7 µL) and $\mathbf{L^f}$ (0.7 µmol, 23.3 µL of a 4 mM stock solution in CD_3CN) at room temperature for 30 minutes in a closed vial to yield 1000 µl of a 0.7 mM solution of c-**B**-$\mathbf{L^f}$. For clearer analysis, only NMR spectra of enantiomerically pure ((R)-c-**B**-$\mathbf{L^f}$) are given. ^{1}H NMR (600 MHz, acetonitrile-d_3) δ 10.23 (s, 2H), 10.16 (s, 2H), 10.10 (d, J = 8.6 Hz, 2H), 10.05 (d, J = 8.6 Hz, 2H), 9.94 (d, J = 8.6 Hz, 2H), 9.72 (s, 2H), 9.12 (s, 4H), 8.94 (s, 2H), 8.57 (dd, 4H), 8.46 (tdd, J = 8.8, 7.1, 1.4 Hz, 4H), 8.11 (m, 4H), 8.09 (dd, J = 8.7, 1.4 Hz, 2H), 7.94 (m, 4H), 7.91 (m, 2H), 7.89 (m, 2H), 7.77 (dd, 4H), 7.73 (m, 2H), 7.71 (m, 2H), 7.63 (ddd, J = 8.0, 1.9, 1.0 Hz, 2H), 7.55 (t, J = 7.8 Hz, 2H), 7.49 (d, J = 2.2 Hz, 2H), 7.47 (dd, J = 8.4, 2.3 Hz, 2H), 7.16 (s, 2H), 7.14 (s, 2H), 7.08 (s, 2H), 7.03 (d, J = 8.3 Hz, 2H), 3.92 (t, J = 7.1 Hz, 2H), 2.50 (s, 6H), 2.45 (s, 6H), 2.41 (s, 6H), 1.75 (p, J = 7.3 Hz, 2H), 1.41 (q, J = 7.2 Hz, 2H), 1.27 (dt, J = 7.2, 3.7 Hz, 4H), 0.83 (td, J = 5.8, 4.6, 3.1 Hz, 3H). ESI-HRMS calculated for $[Pd_2(c\text{-}\mathbf{L^a})_3\mathbf{L^f}+BF_4]^{3+}$ m/z = 918.7851, found m/z = 918.7906.

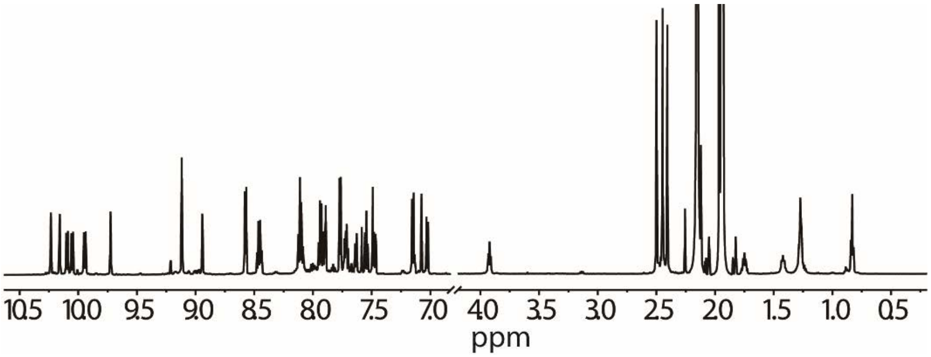

Figure S41. ^{1}H NMR spectrum (600 MHz, acetonitrile-d_3) of (R)-c-**B**-$\mathbf{L^f}$.

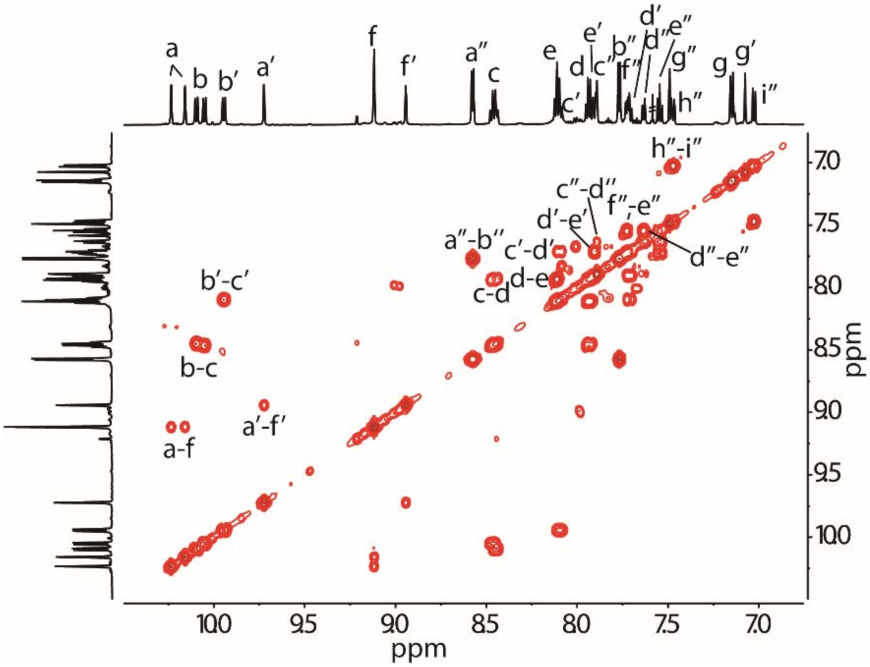

Figure S42. ^{1}H–^{1}H COSY spectrum (600 MHz, acetonitrile-d_3) of (R)-c-**B**-$\mathbf{L^f}$ (only showing aromatic region).

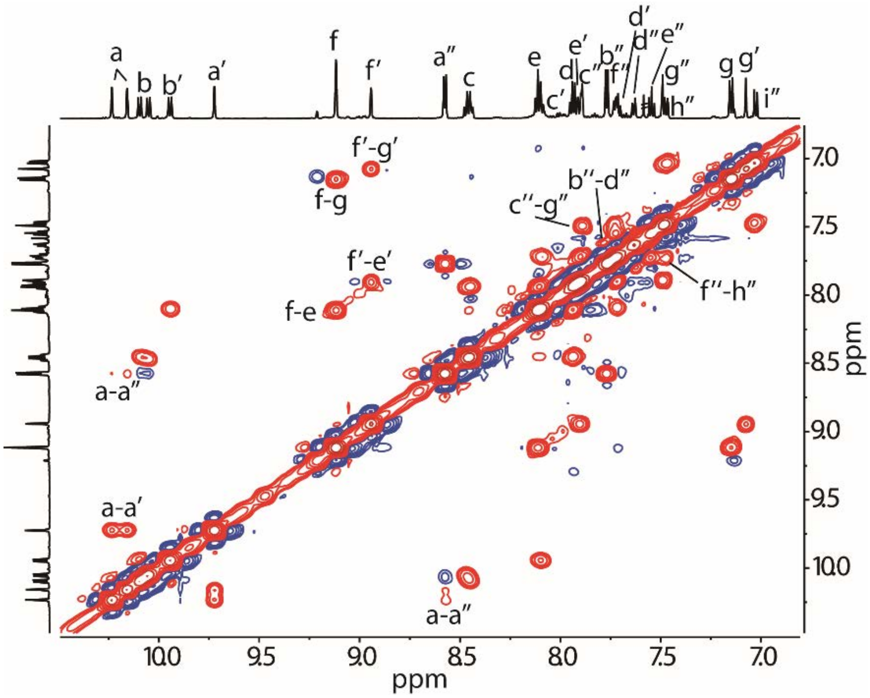

Figure S43. ^1H–^1H NOESY spectrum (600 MHz, acetonitrile-d_3) of (*R*)-*c*-**B**-**L**$^{\mathbf{f}}$ (only showing aromatic region).

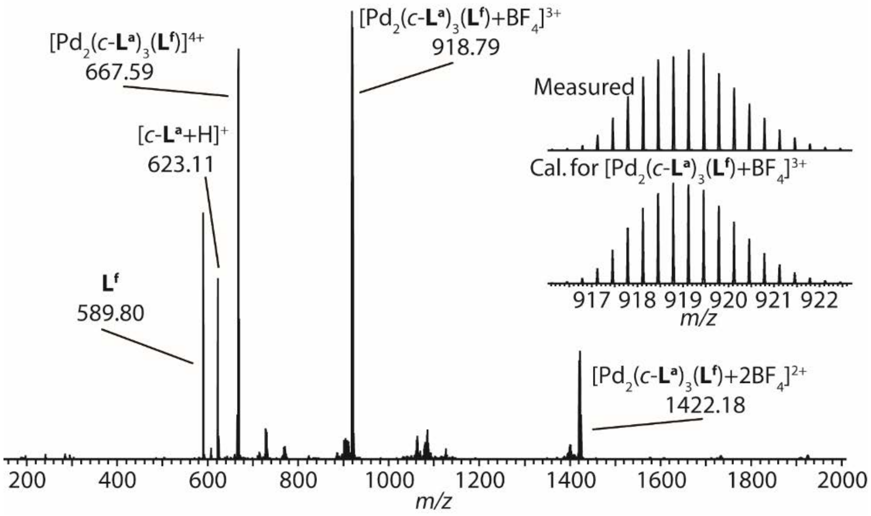

Figure S44. ESI-HRMS spectrum of *c*-**B**-**L**$^{\mathbf{f}}$.

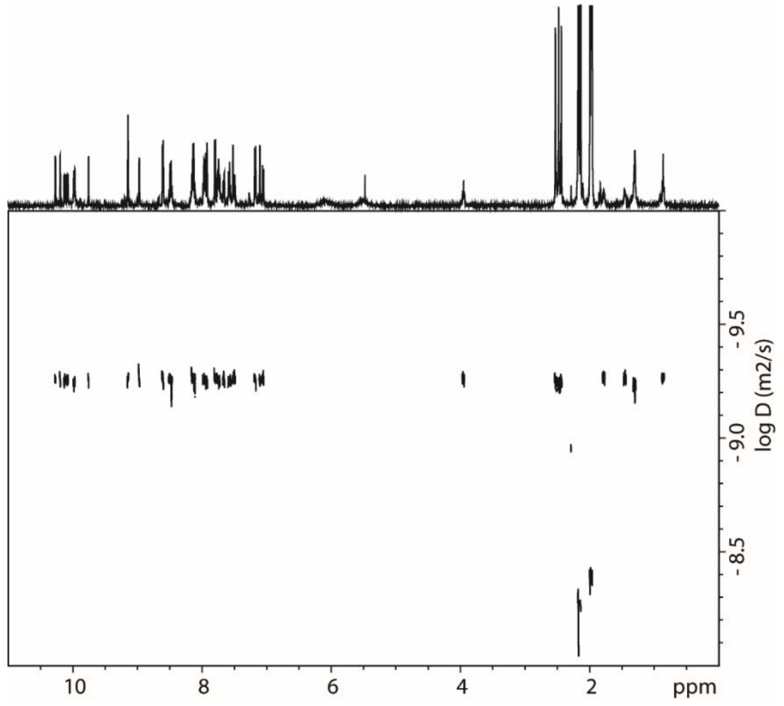

Figure S45. DOSY NMR (500 MHz, 298 K acetonitrile-d_3) spectrum of (*R*)-*c*-**B**-**L**$^{\mathbf{f}}$.

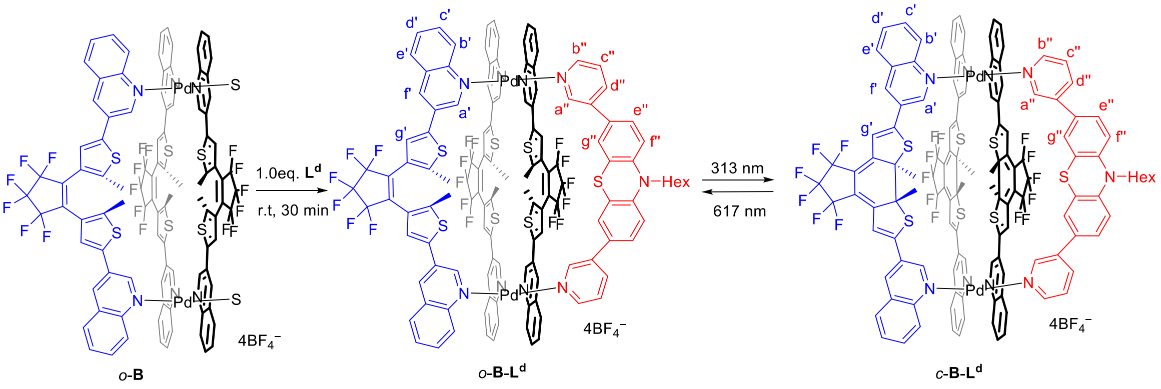

Scheme S12 Synthesis of *o*-**B**-**L**$^{\mathbf{d}}$.

The heteroleptic cage $[Pd_2(o\text{-}\mathbf{L}^{\mathbf{a}})_3\mathbf{L}^{\mathbf{d}}](BF_4)_4$ (*o*-**B**-**L**$^{\mathbf{d}}$) can be synthesized in the same way as the analogous heteroleptic cage, with the backbone in its closed form. Stirring a mixture of $[Pd_2(o\text{-}\mathbf{L}^{\mathbf{a}})_3(CH_3CN)_2](BF_4)_4$ in CD_3CN (0.7 µmol, 976.7 µL) and **L**$^{\mathbf{d}}$ (0.7 µmol, 23.3 µL of a 4 mM stock solution in CD_3CN) at room temperature for 30 minutes in a closed vial yield 1000 µl of a 0.7 mM solution of *o*-**B**-**L**$^{\mathbf{d}}$. It can also be obtained from *c*-**B**-**L**$^{\mathbf{d}}$ by light irradiation under 617 nm. However, the proton NMR suffer broadening of the signals. **G1@***o*-**B**-**L**$^{\mathbf{d}}$ ^{1}H NMR spectrum is shown here, allowing to analyze clearer signals. ESI-HRMS calculated for $[Pd_2(o\text{-}\mathbf{L}^{\mathbf{a}})_3(\mathbf{L}^{\mathbf{d}})]^{4+}$ *m/z* = 868.0991, found *m/z* = 868.1021.

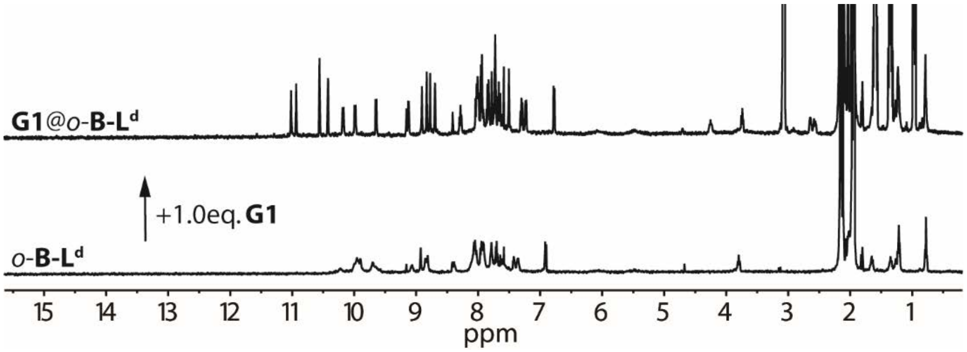

Figure S46. ^{1}H NMR spectrum (500 MHz, acetonitrile-d_3) of *o*-**B**-**L**$^{\mathbf{d}}$ and **G1**@ *o*-**B**-**L**$^{\mathbf{d}}$.

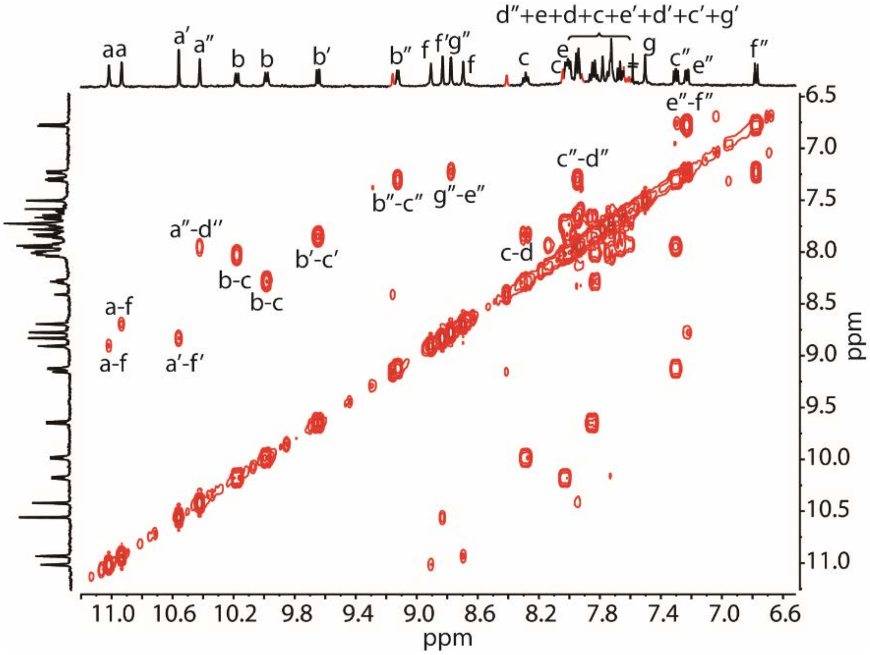

Figure S47. ^{1}H–^{1}H COSY spectrum (500 MHz, acetonitrile-d_3) of **G1**@ *o*-**B**-**L**$^{\mathbf{d}}$ (only showing aromatic region).

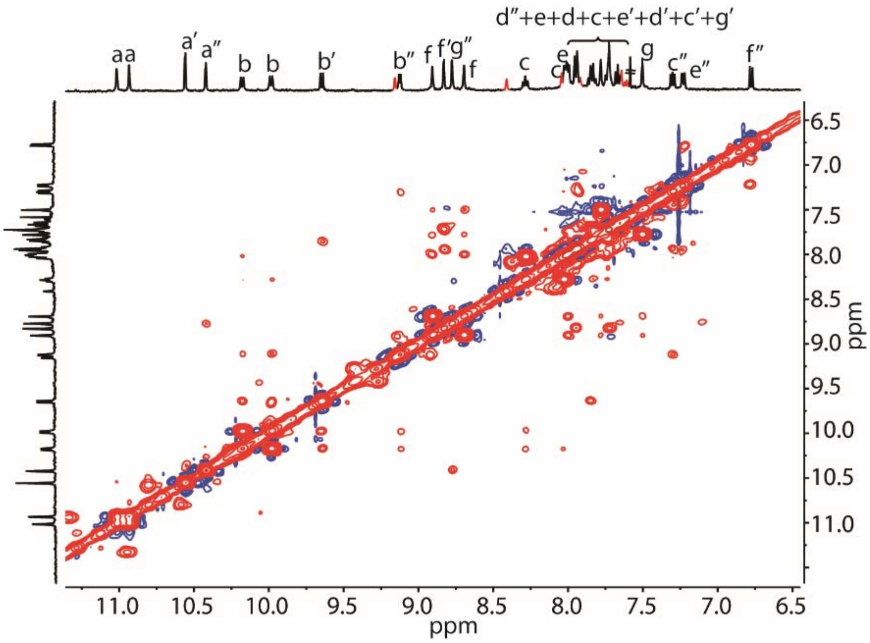

Figure S48. ^{1}H–^{1}H NOESY spectrum (500 MHz, acetonitrile-d_3) of **G1@** *o*-**B**-$\mathbf{L^d}$ (only showing aromatic region).

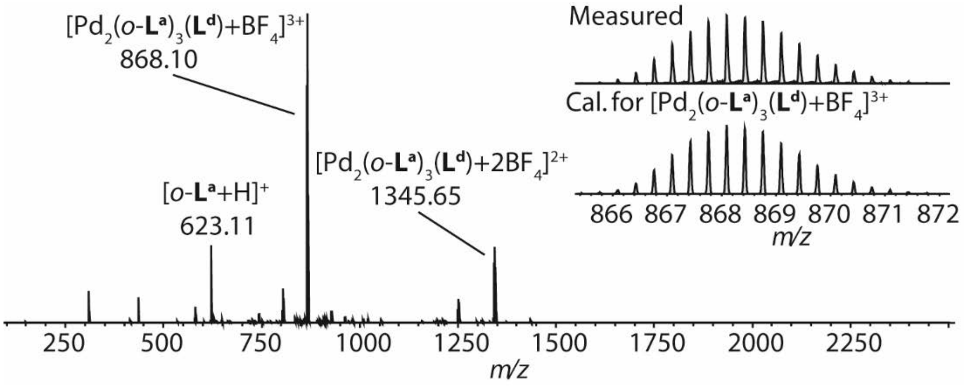

Figure S49. ESI-HRMS spectrum of *o*-**B**-$\mathbf{L^d}$.

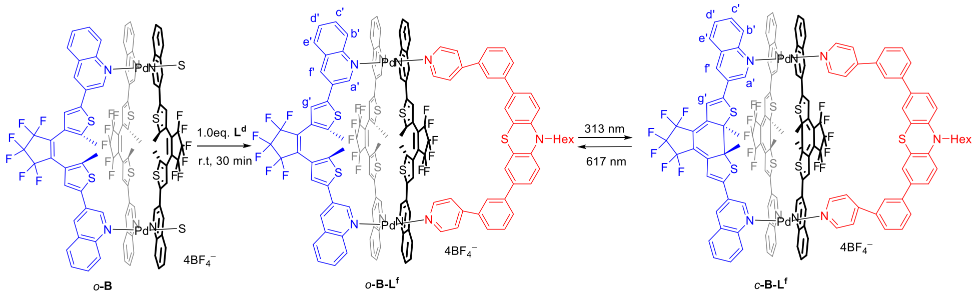

Scheme S13 Synthesis of *o*-**B**-$\mathbf{L^f}$.

Similarly, the heteroleptic cage $[Pd_2(o\text{-}\mathbf{L^a})_3\mathbf{L^f}](BF_4)_4$ (*o*-**B**-$\mathbf{L^f}$) can be synthesized in the same way as the *c*-**B**-$\mathbf{L^f}$ analogous cage with the backbone in its close form. Stirring a mixture of $[Pd_2(o\text{-}\mathbf{L^a})_3(CH_3CN)_2](BF_4)_4$ in CD_3CN (0.7 μmol, 976.7 μL) and $\mathbf{L^f}$ (0.7 μmol, 23.3 μL of a 4 mM stock solution in CD_3CN) at room

temperature for 30 minutes in a closed vial, yield 1000 µl of a 0.7 mM solution of *o*-**B**-**L**$^{\mathbf{f}}$. It can also be obtained from *c*-**B**-**L**$^{\mathbf{f}}$ by light irradiation under 617 nm. ESI-HRMS calculated for $[Pd_2(o\text{-}\mathbf{L^a})_3(\mathbf{L^f})]^{4+}$ *m/z* = 918.7868, found *m/z* = 918.7901.

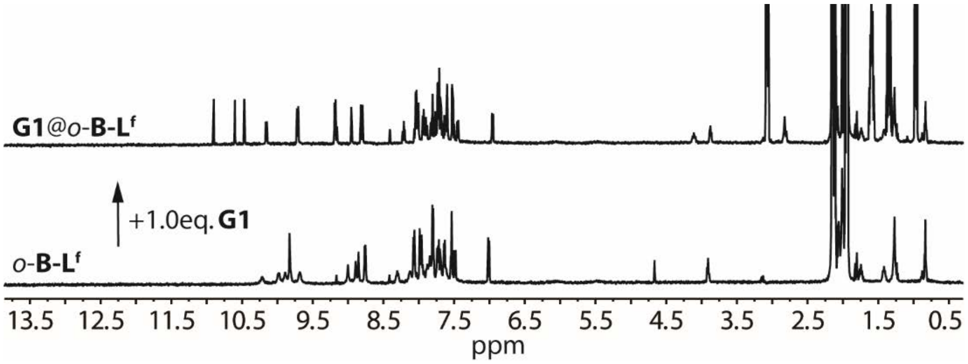

Figure S50. ^{1}H NMR spectrum (500 MHz, acetonitrile-d_3) of *o*-**B**-**L**$^{\mathbf{f}}$ and **G1**@ *o*-**B**-**L**$^{\mathbf{f}}$.

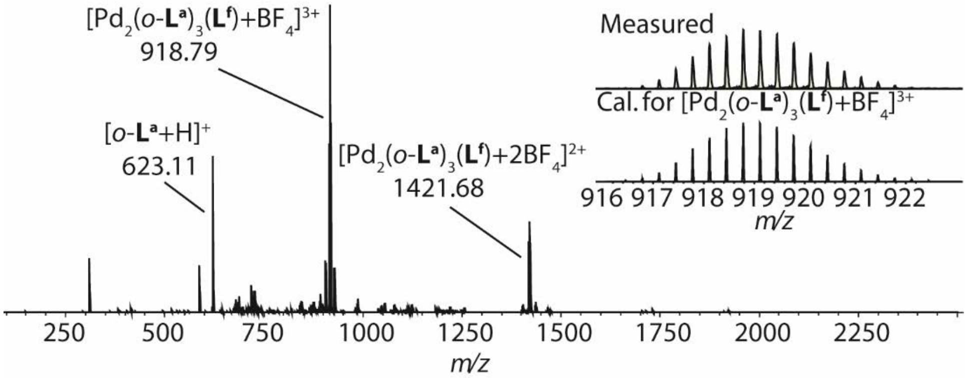

Figure S51. ESI-HRMS spectrum of *o*-**B**-**L**$^{\mathbf{f}}$.

3. Symmetry

Similar to our previous reported cage,[S1] there are 6 possible stereoisomers of the bowl-like complex *c*-**B**: three pairs of enantiomers *PPP*/*MMM* (I), *PMM*/*MPP* (II) and *PMP/MPM* (III) that differ in the composition concerning *P*- or *M*-configured, *C*2-symmetric ligands *c*-**L**$^{\mathbf{a}}$ occupying the three positions around the two metal centers. According to a statistical analysis based on the assumption that all isomers are energetically equal, their ratio (I) : (II) : (III) is (1+1) : (2+2) : (1+1) (Σ=8). However, for each enantiomer *PMM*/*MPP* (II), there are two different top views. Therefore, there would be four different chemical environments of *c*-**B** with 2 : 2 : 2 : 2 ratio (see the following figure). Which was consistent with the experimental ^{1}H NMR spectrum of *c*-**B**. We can clearly see four sets of NMR signals, especially for $H_{a'}$ and $H_{g'}$, giving a 1 : 1 : 1 : 1 ratio.

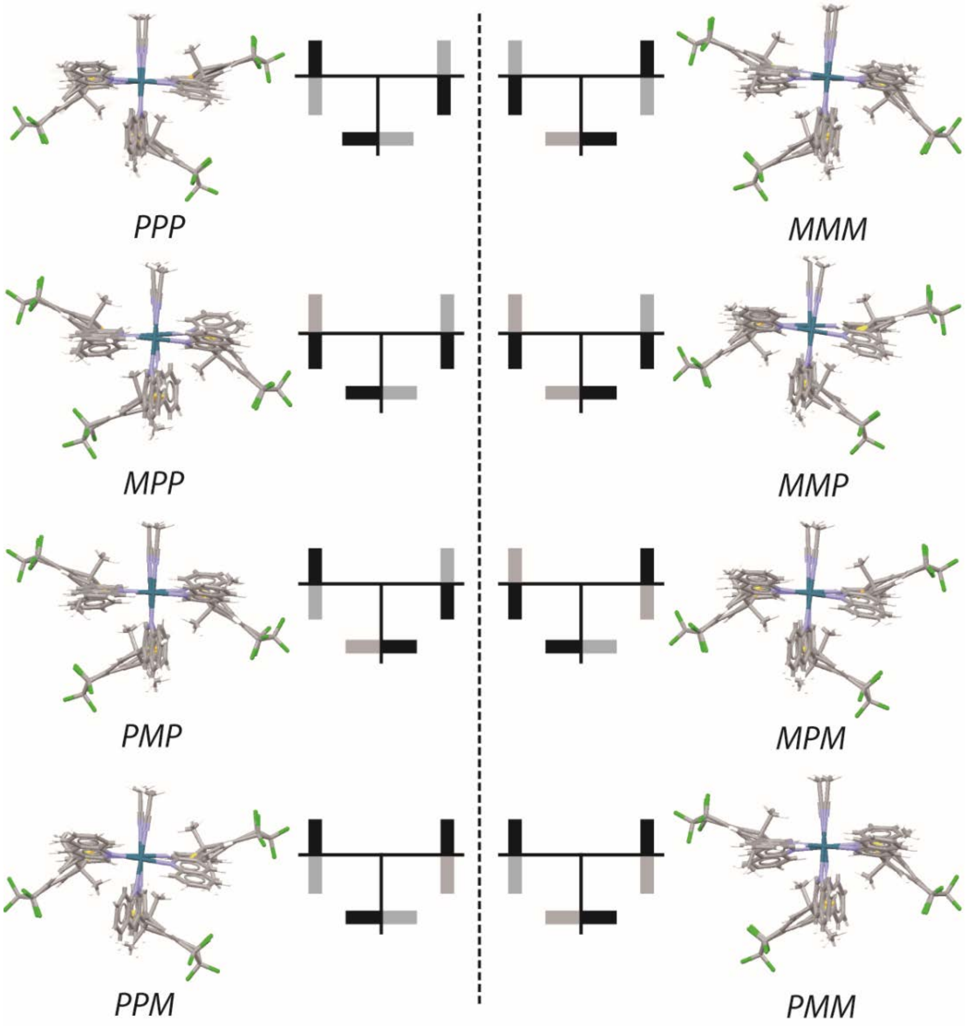

Figure S52. All possible isomeric bowl structures (built using the software Spartan '14) and their corresponding stereo-chemical relationships between the three ligands viewed from the top (black/gray bars: close/remote methyl groups).

4. Absolute configuration

The open-form ligand *o*-**L**^{a} (10 mg) was dissolved in CD_2Cl_2 (1 mL) and then irradiated with 313 nm UV light. The pure (*R,R*) and (*S,S*) ligand enantiomers were separated by preparative chiral HPLC using a Daicel IC column (250 x 10 mm) under strict exclusion of daylight. The purity of separated **1** (the first fraction of HPLC) and **2** (the second fraction of HPLC) enantiomers were checked using analytical HPLC using a Daicel IA column (250 x 4.6 mm). In order to determine the absolute stereochemistry of the enantiomers, CD spectra were calculated by TD-DFT methods at the B3LYP/6-31G(d) level of theory in the Gaussian 09 software[S2] (using keyword iop(9/40=2)).

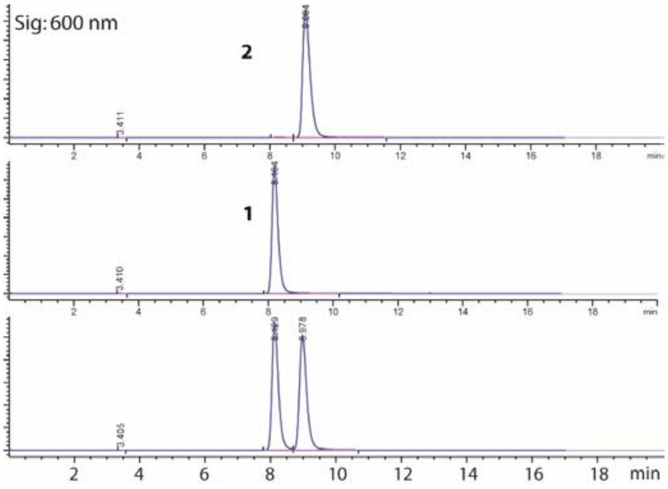

Figure S53. Chiral HPLC chromatograms of racemic *c*-**L**[a]. (Chromatographic column: Daicel IC. Mobile phase: hexane/ CH_2Cl_2/methanol = 60 : 39.1 : 0.9. Run time: 20 min. Flow rate: 1.0 mL/min).

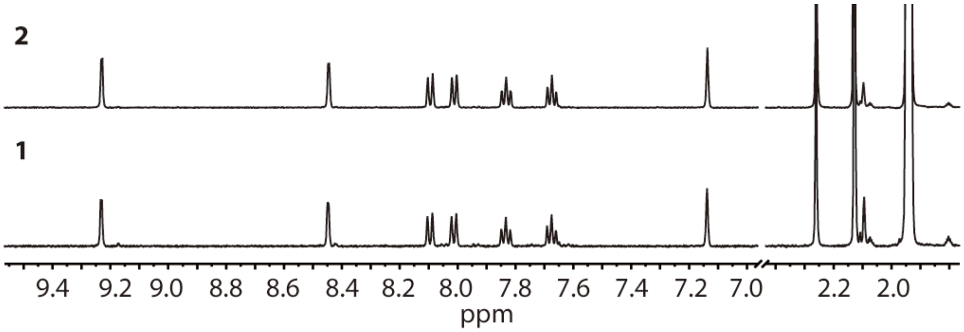

Figure S54. ^{1}H NMR spectra (500 MHz, acetonitrile-d_3) of enantiomeric pure fraction **1** and fraction **2**.

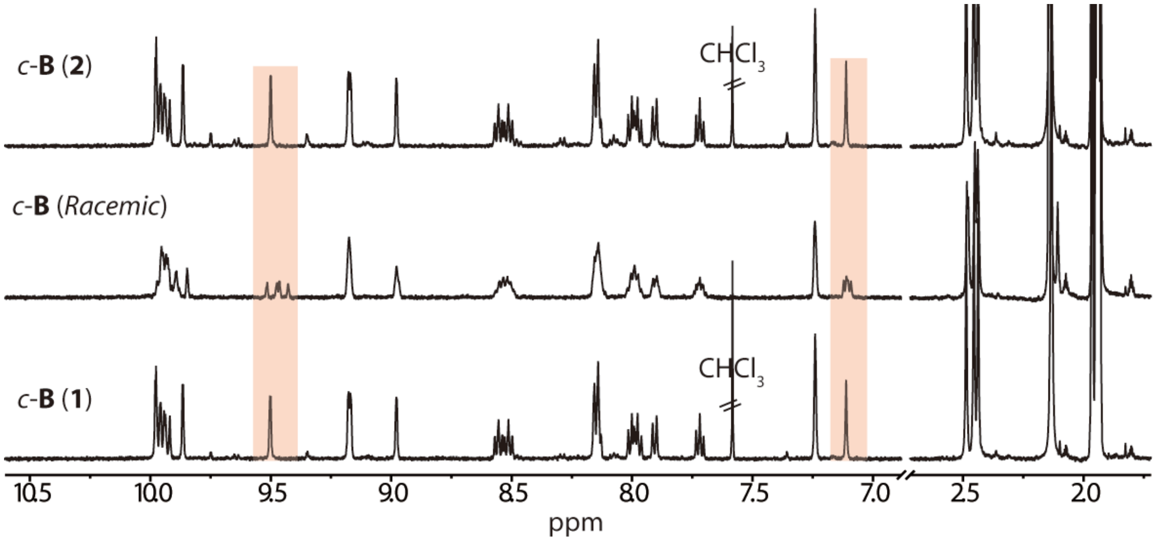

Figure S55. ^{1}H NMR spectra (500 MHz, acetonitrile-d_3) of racemic *c*-**B** and enantiomeric pure *c*-**B** (**1**) and *c*-**B** (**2**) made from enantiomerically pure ligands **1** and **2**, respectively. As can be seen, the signals of the enantiomeric pure cages *c*-**B** are sharper and better resolved than those of the racemic mixture of all possible *c*-**B** diastereomers. From the red marked signals, there are four times ^{1}H NMR diastereomeric splitting, which was consistence with symmetry analysis above.

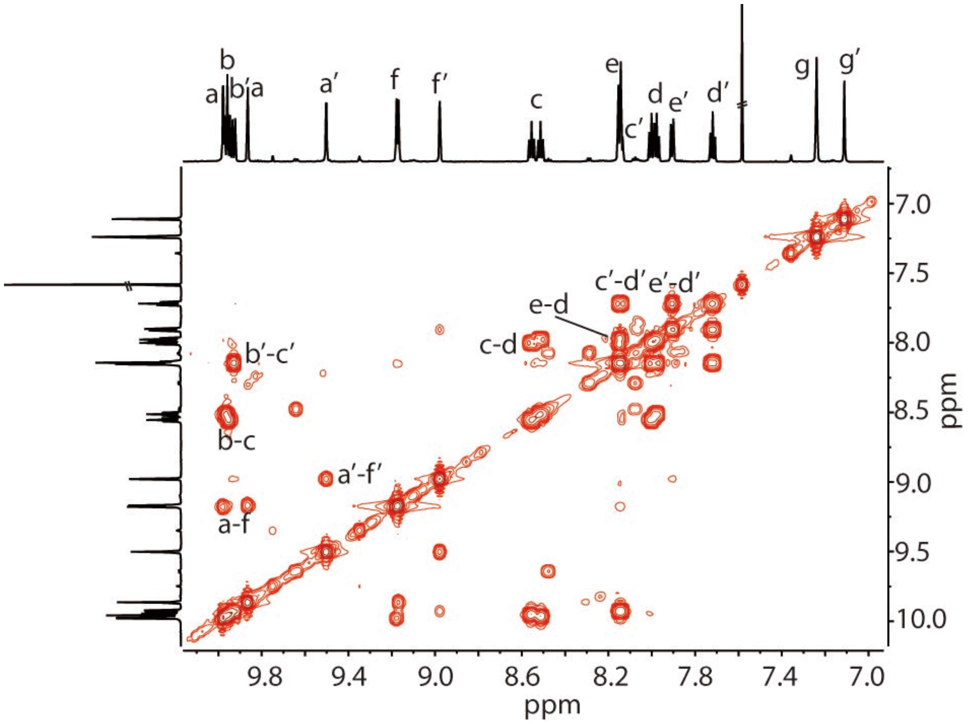

Figure S56. ^1H–^1H COSY spectrum (600 MHz, acetonitrile-d_3) of *c*-**B** (**1**) (only showing aromatic region).

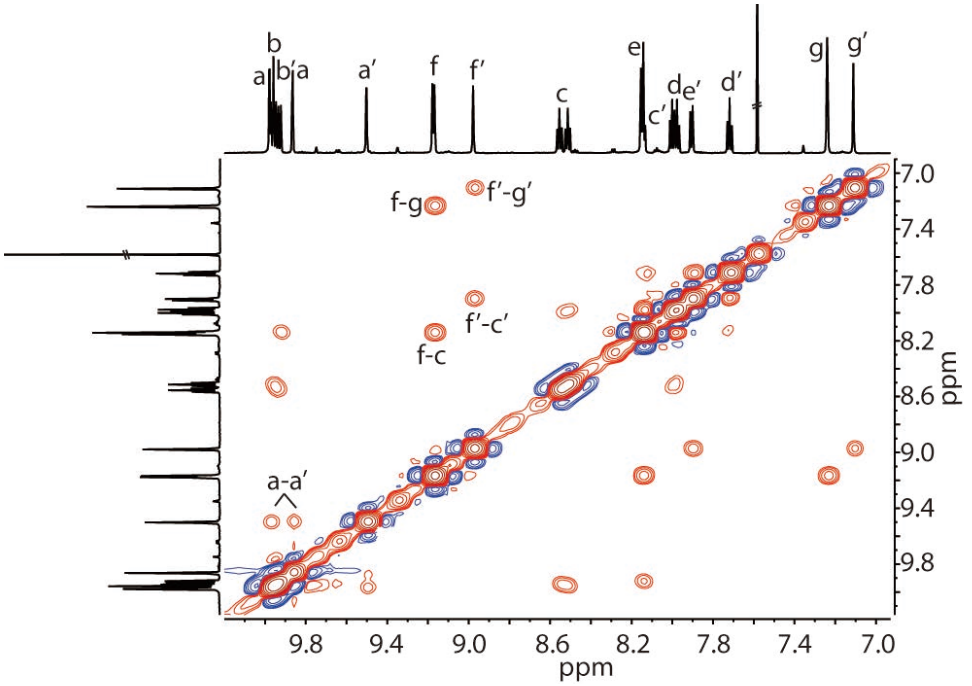

Figure S57. ^1H–^1H NOESY spectrum (600 MHz, acetonitrile-d_3) of *c*-**B** (**1**) (only showing aromatic region).

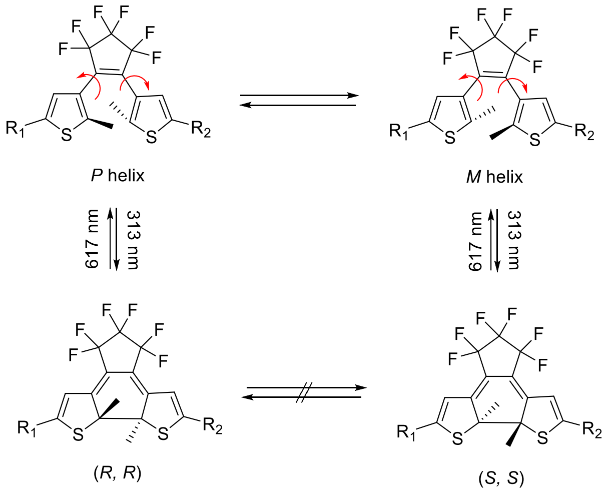

Scheme S14. Photoisomerization of a diarylethene (DTE). The photocyclization reactions from *P*- and *M*-helical conformers of the open-ring isomer produce (*R,R*) and (*S,S*) enantiomers of the closed-ring isomer, respectively.

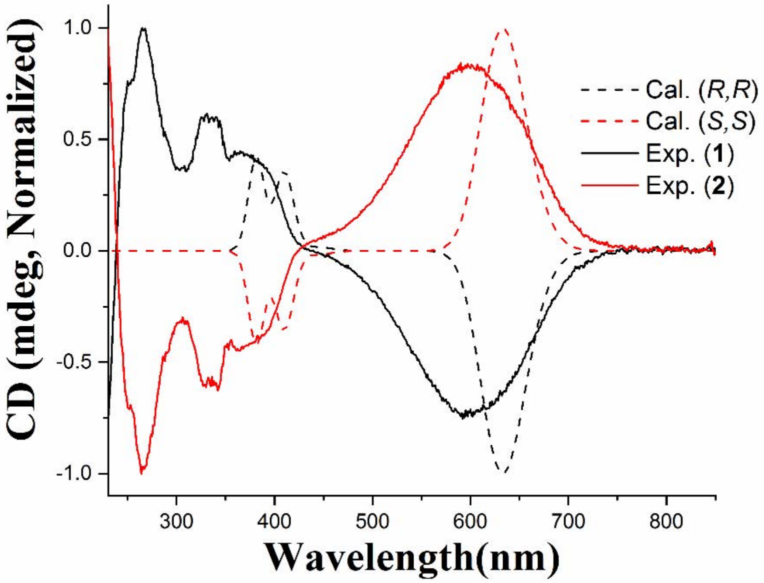

Figure S58. a) Calculated CD spectra of (*R,R*) and (*S,S*) closed form ligand enantiomers and measured CD spectra of closed ligand enantiomers from chiral HPLC resolved fraction **1** and **2** (0.1 mM, CH_3CN);. Both of the calculated CD spectrum of *c*-**L** (*R,R*) and fraction **1** give negative cotton effect from 500 nm to 800 nm (this area shows the characteristic peak of closed form DTE derives), which means fraction **1** has *R,R* configuration, in contrast, fraction **2** is *S,S* configuration.

5. Computational studies

All models were constructed using SPARTAN and were first optimized on a PM6 level of theory (no counter ions were included) without constrains. The resulting structures were than further refined by DFT gas-phase calculations (B3LYP/LANL2DZ) using Gaussian 09 software.

5.1 Optimized structure of heteroleptic cages

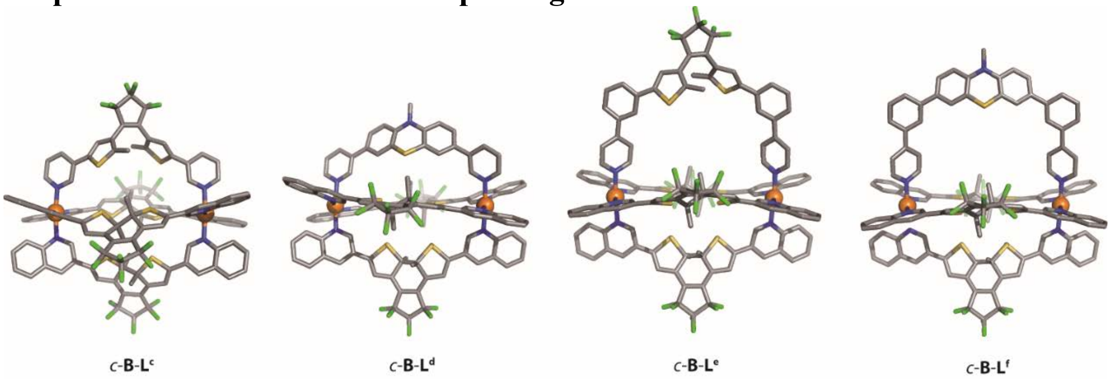

Figure S59. DFT energy minimized structures of *o*-**B**-**L**^{c}, *o*-**B**-**L**^{d}, *o*-**B**-**L**^{e} and *o*-**B**-**L**^{f}.

5.2 Comparison of the energy between cage and bowl structures

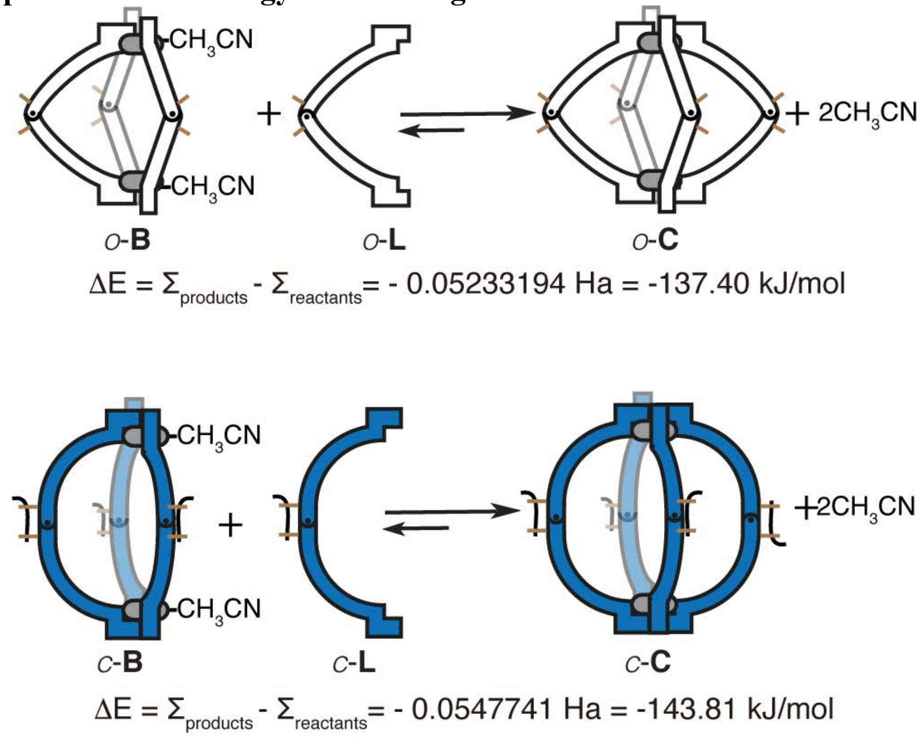

Figure S60. Scheme showing reaction equations between cages & bowls and their reaction energies as obtained from respective DFT gas-phase minimized structures.

6. Titrations

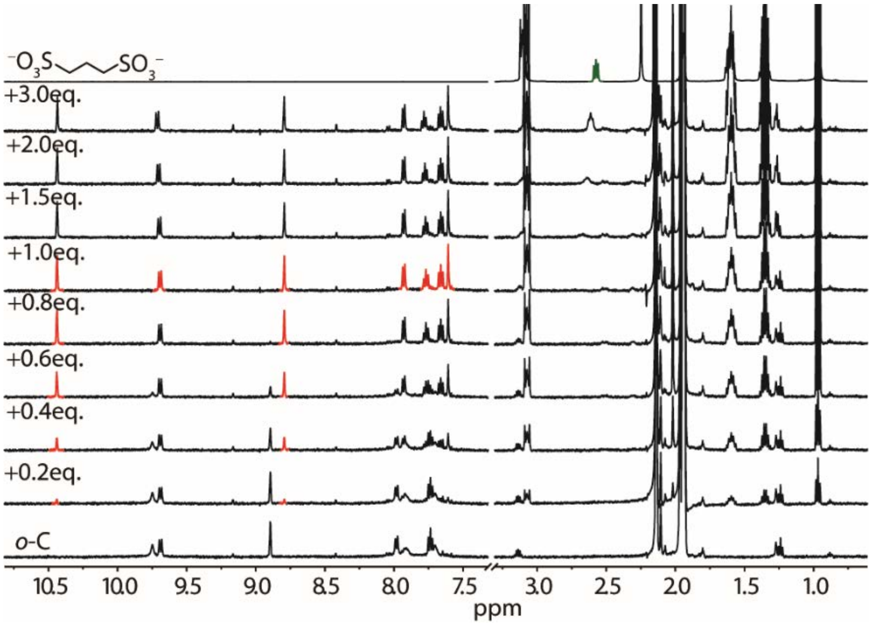

Figure S61. ^{1}H NMR titration (500 MHz, 298 K, acetonitrile-d_3) of *o*-**C** with $(NBu_4)_2$**G1**. Upon addition of one equivalent of guest **G1**, the *o*-**C** cage transforms into [**G1**@*o*-**C**]. Excess addition of **G1** leads to precipitation. New set of NMR signals is highlighted in red.

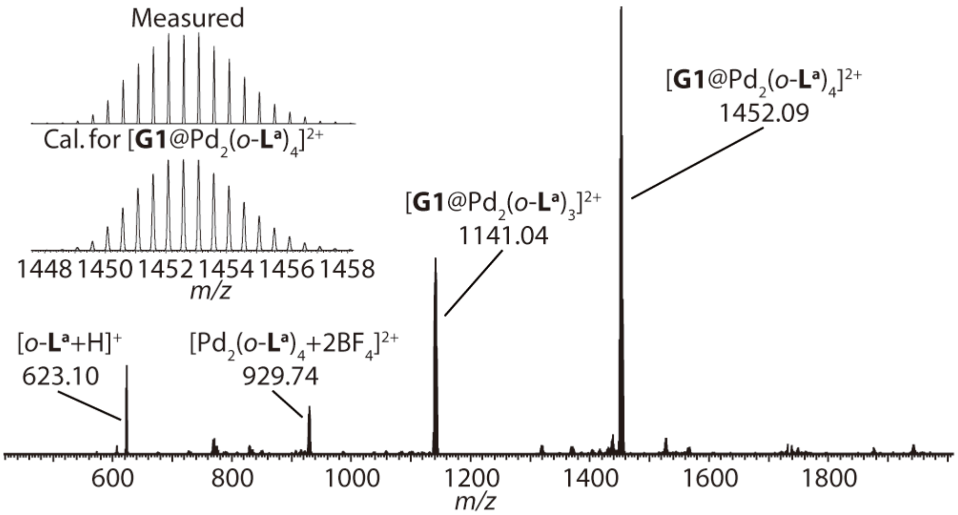

Figure S62. ESI-HRMS spectrum of [**G1**@*o*-**C**].

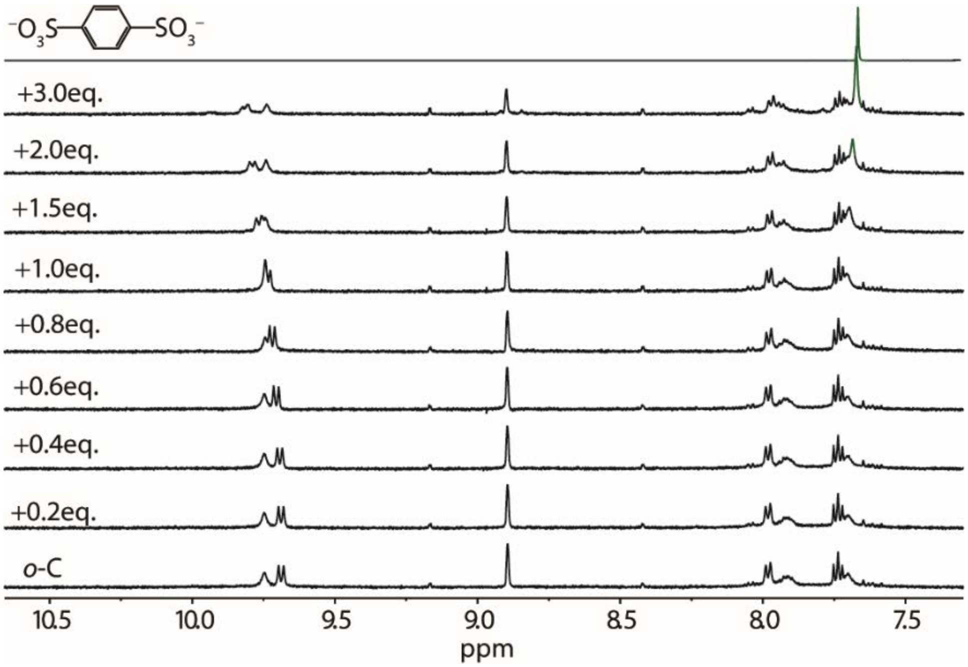

Figure S63. ^{1}H NMR titration (500 MHz, 298 K, acetonitrile-d_3) of *o*-**C** with $(NBu_4)_2$**G2**. Upon addition of guest **G2**, inward-pointing proton H_a did not show any shifting, while outward-pointing proton H_a shows downfield shifting, indicating that the larger size guest **G2** cannot be encapsulated. Excess addition of **G2** leads to precipitation.

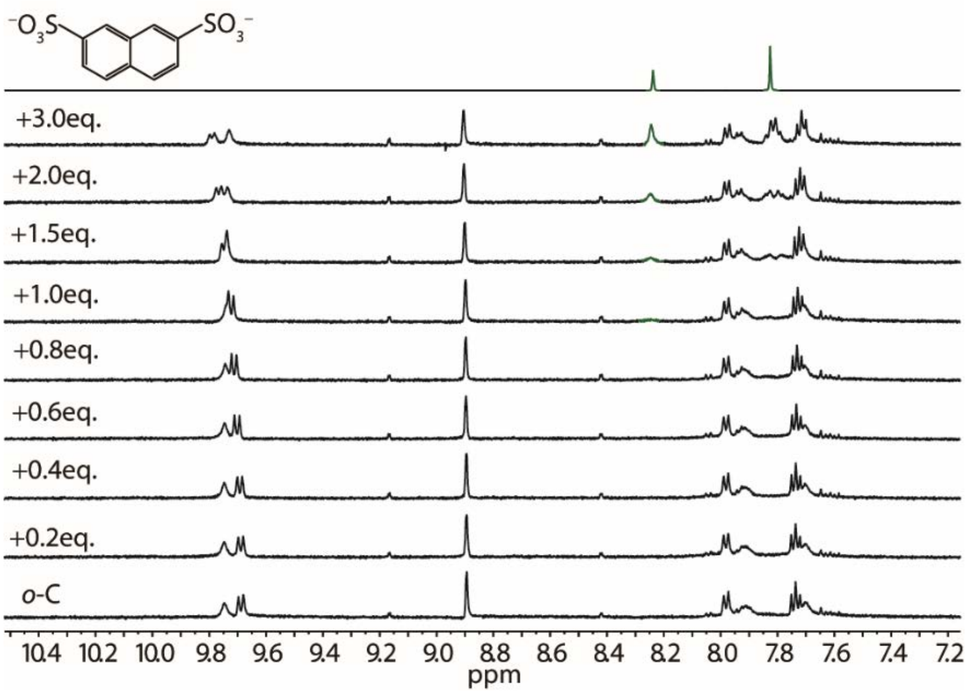

Figure S64. ^{1}H NMR titration (500 MHz, 298 K, acetonitrile-d_3) of *o*-**C** with $(NBu_4)_2$**G3**. Similar to $(NBu_4)_2$**G2**, upon addition of guest **G3**, only outward-pointing proton H_a shows downfield shifting, indicating that the larger size guest **G3** cannot be encapsulated. Excess addition of **G3** leads to precipitation.

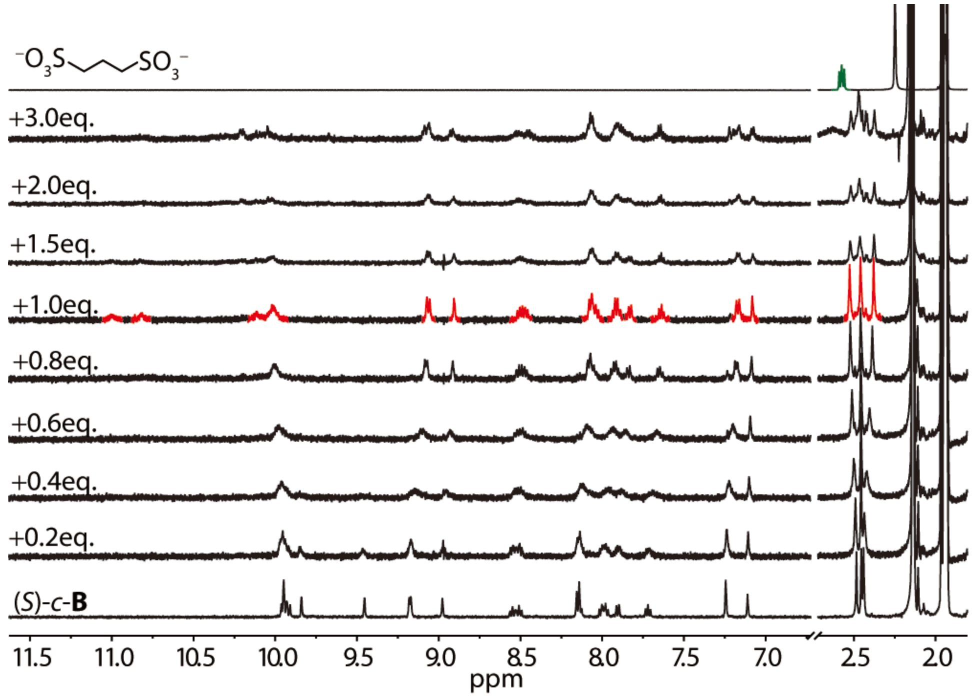

Figure S65. ^{1}H NMR titration (500 MHz, 298 K, acetonitrile-d_3) of (*S*)-*c*-**B** with $(NBu_4)_2$**G1**. Upon addition of one equivalent of guest **G1**, the (*S*)-*c*-**B** cage transforms into [**G1**@(*S*)-*c*-**B**]. Excess addition of **G1** leads to precipitation. New set of NMR signals is highlighted in red.

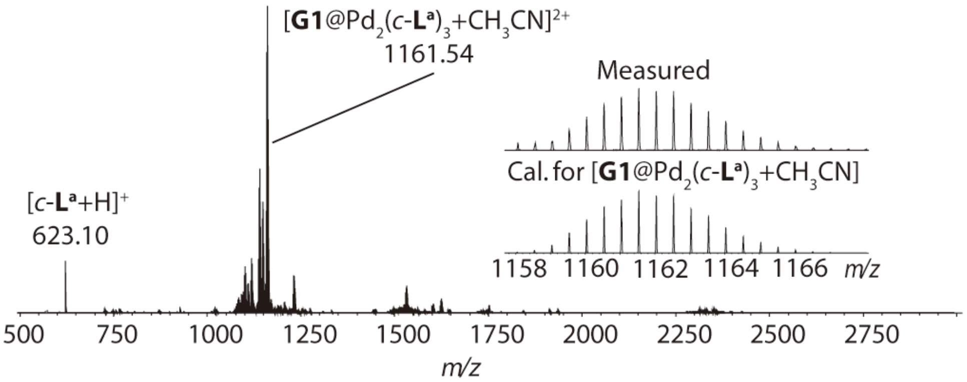

Figure S66. ESI-HRMS spectrum of [**G1**@*c*-**B**].

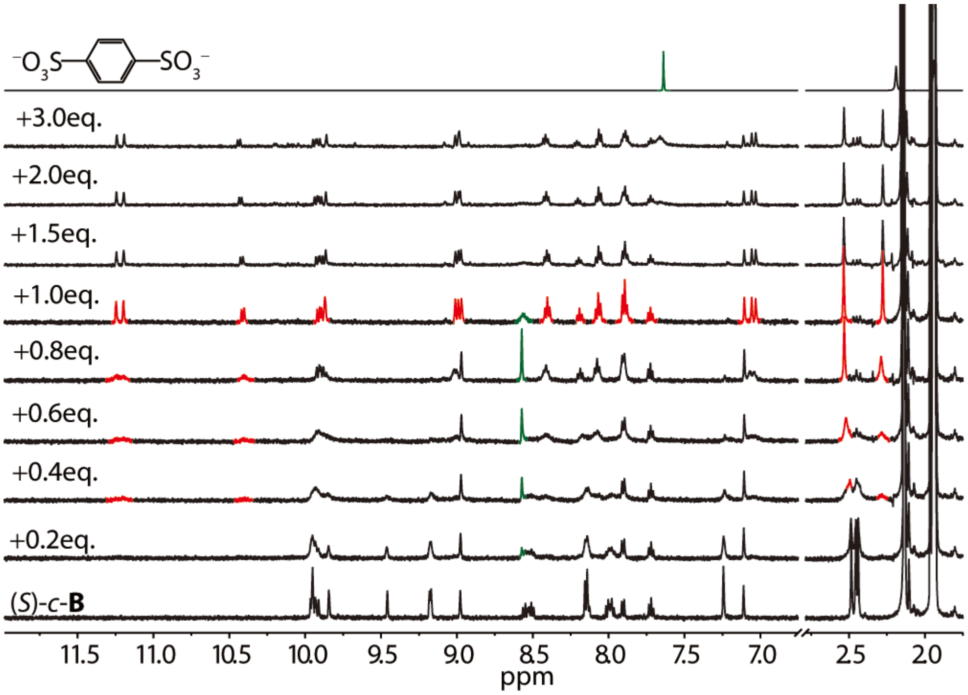

Figure S67. ^{1}H NMR titration (500 MHz, 298 K, acetonitrile-d_3) of (*S*)-*c*-**B** with $(NBu_4)_2$**G2**. Upon addition of one equivalent of guest **G2**, the (*S*)-*c*-**B** cage transforms into [**G2**@(*S*)-*c*-**B**]. New set of NMR signals is highlighted in red. Signal of encapsulated **G2** is highlighted in green.

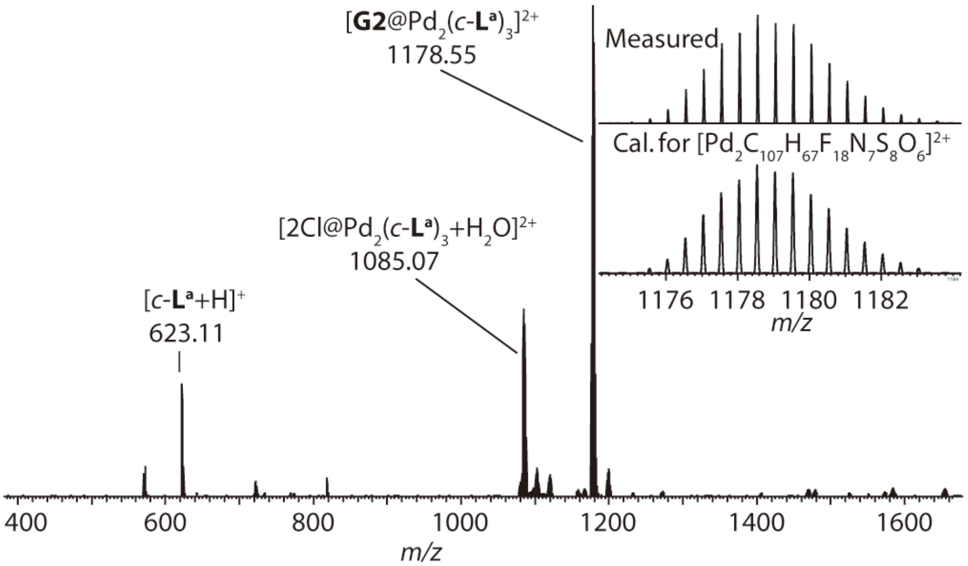

Figure S68. ESI-HRMS spectrum of [**G2**@*c*-**B**].

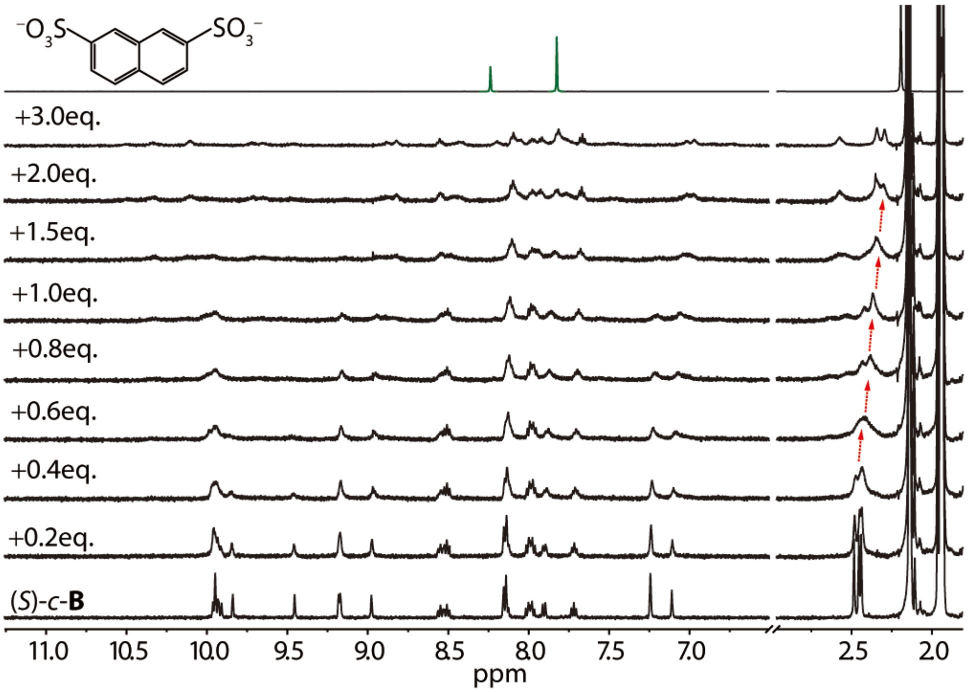

Figure S69. ^{1}H NMR titration (500 MHz, 298 K, acetonitrile-d_3) of (*S*)-*c*-**B** with $(NBu_4)_2$**G3**. Upon addition of one equivalent of guest **G3**, the (*S*)-*c*-**B** cage transforms into [**G3**@(*S*)-*c*-**B**]. Excess addition of **G3** leads to precipitation.

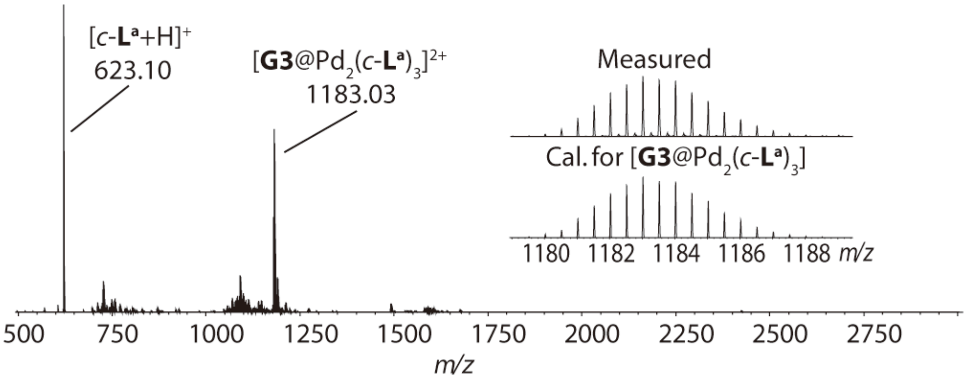

Figure S70. ESI-HRMS spectrum of [**G3**@*c*-**B**].

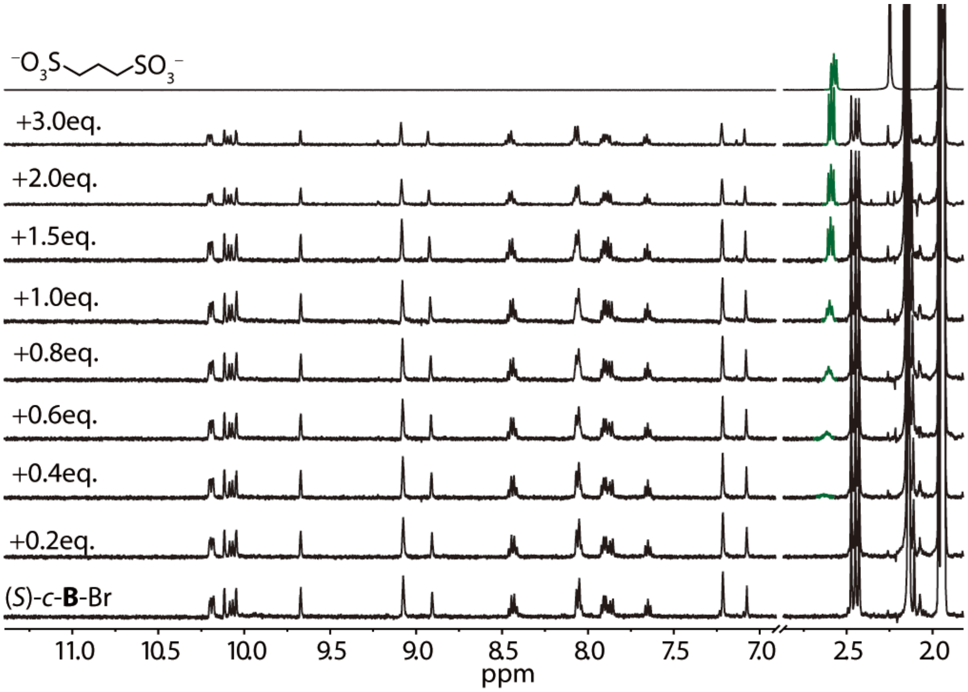

Figure S71. ^{1}H NMR titration (500 MHz, 298 K, acetonitrile-d_3) of (*S*)-*c*-**B**-**Br** with $(NBu_4)_2$**G1**. Upon addition of guest **G1**, both inward-pointing and outward-pointing proton did not show any shifting, indicating that there is no encapsulation or interaction of **G1**. Excess addition of **G1** leads to precipitation.

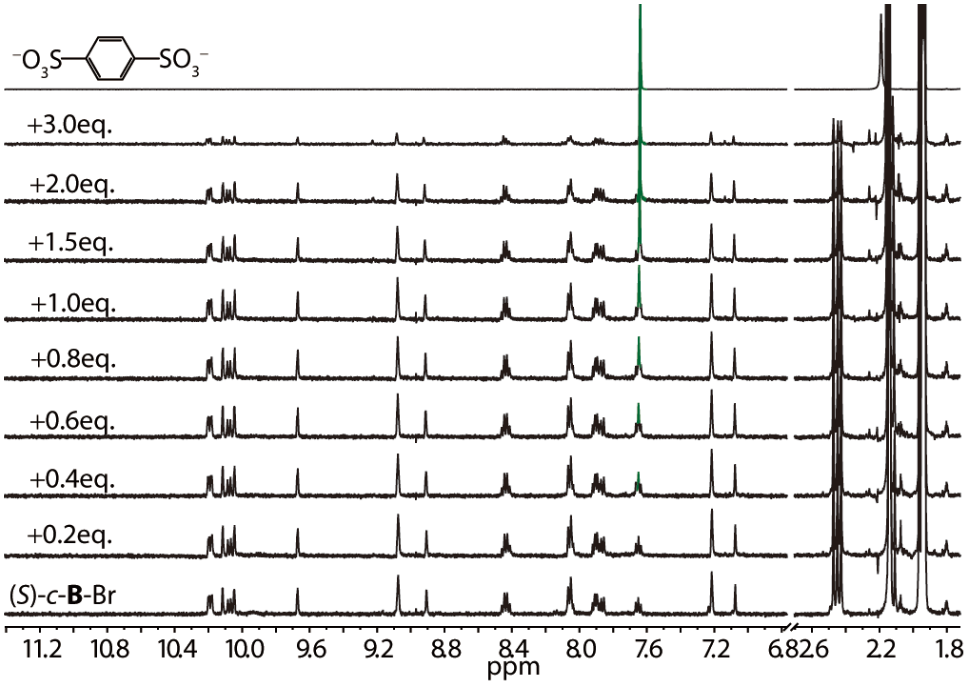

Figure S72. ^{1}H NMR titration (500 MHz, 298 K, acetonitrile-d_3) of (*S*)-*c*-**B**-**Br** with $(NBu_4)_2$**G2**. Upon addition of guest **G2**, both inward-pointing and outward-pointing proton did not show any shifting, indicating that there is no encapsulation or interaction of **G2**. Excess addition of **G2** leads to precipitation.

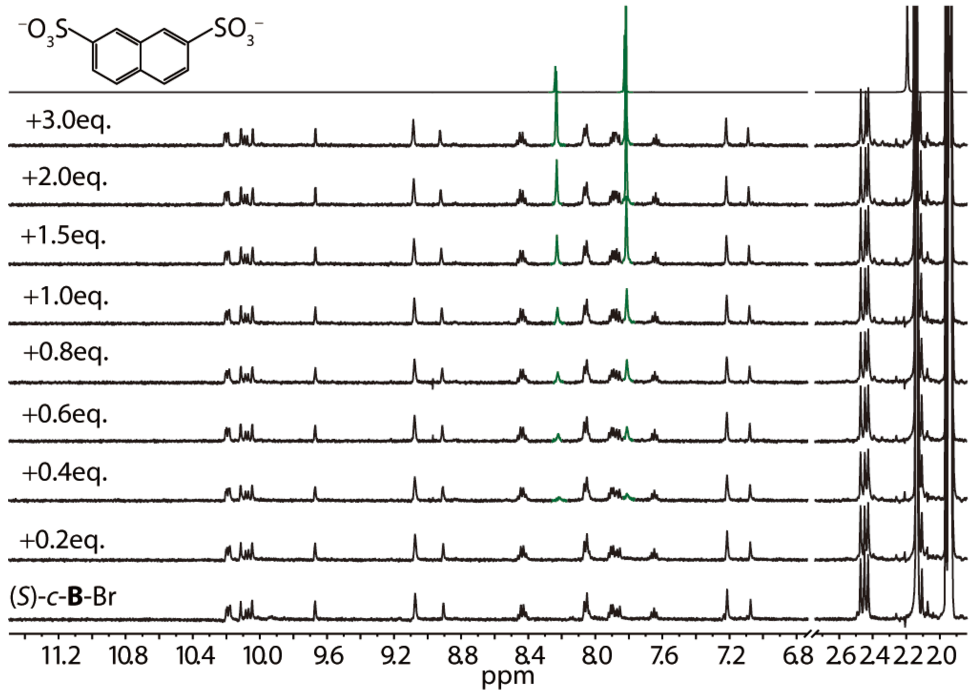

Figure S73. ^{1}H NMR titration (500 MHz, 298 K, acetonitrile-d_3) of (*S*)-*c*-**B-Br** with $(NBu_4)_2$**G3**. Upon addition of guest **G3**, both inward-pointing and outward-pointing protons did not show any shifting, indicating that there is no encapsulation or interaction of **G3**. Excess addition of **G3** leads to precipitation.

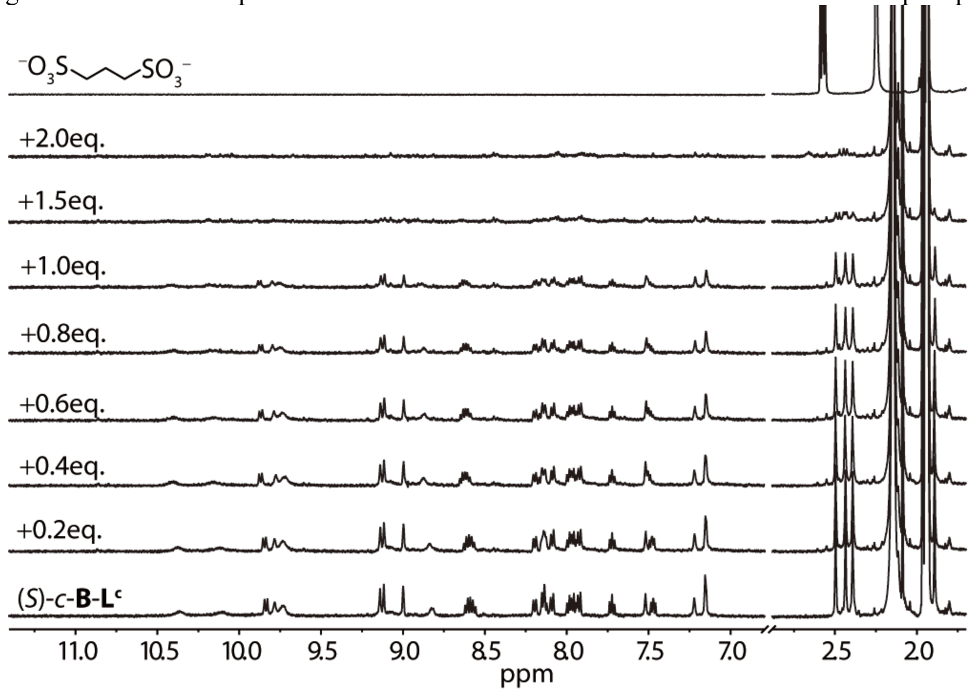

Figure S74. ^{1}H NMR titration (500 MHz, 298 K, acetonitrile-d_3) of (*S*)-*c*-**B-L**^{c} with $(NBu_4)_2$**G1**. Upon addition of guest **G1**, outward-pointing protons H_b and $H_{b'}$ show broadening, indicating that the guest **G1** cannot be encapsulated. Excess addition of **G1** leads to precipitation.

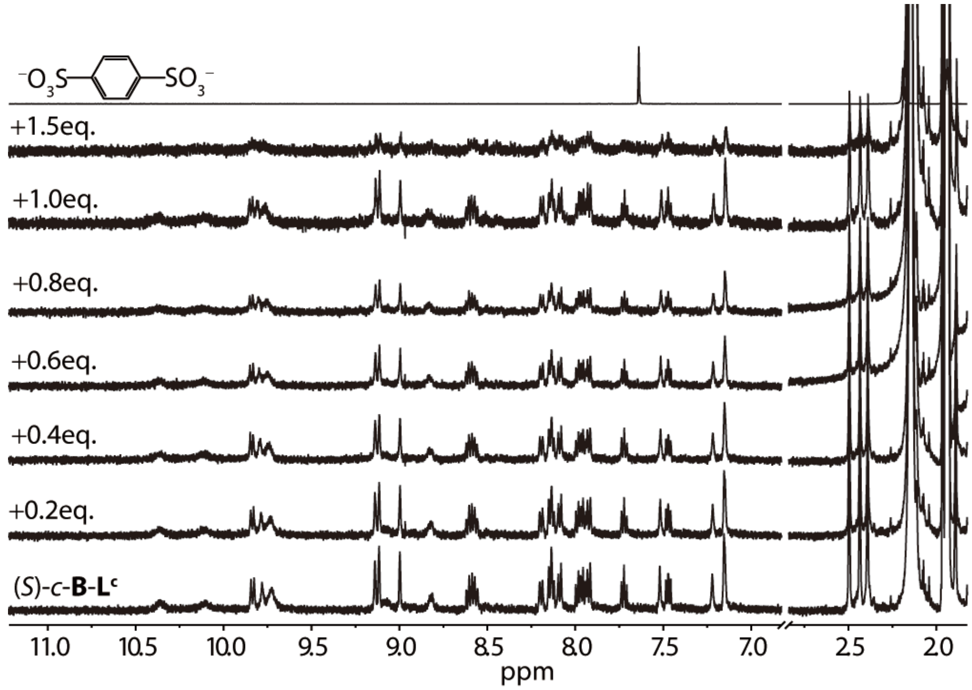

Figure S75. ^{1}H NMR titration (500 MHz, 298 K, acetonitrile-d_3) of (*S*)-*c*-**B**-**L**^{c} with $(NBu_4)_2$**G2**. Upon addition of guest **G2**, precipitation occurs immediately. Outward-pointing protons H_b and $H_{b'}$ show significant broadening, indicating that the guest **G2** cannot be encapsulated.

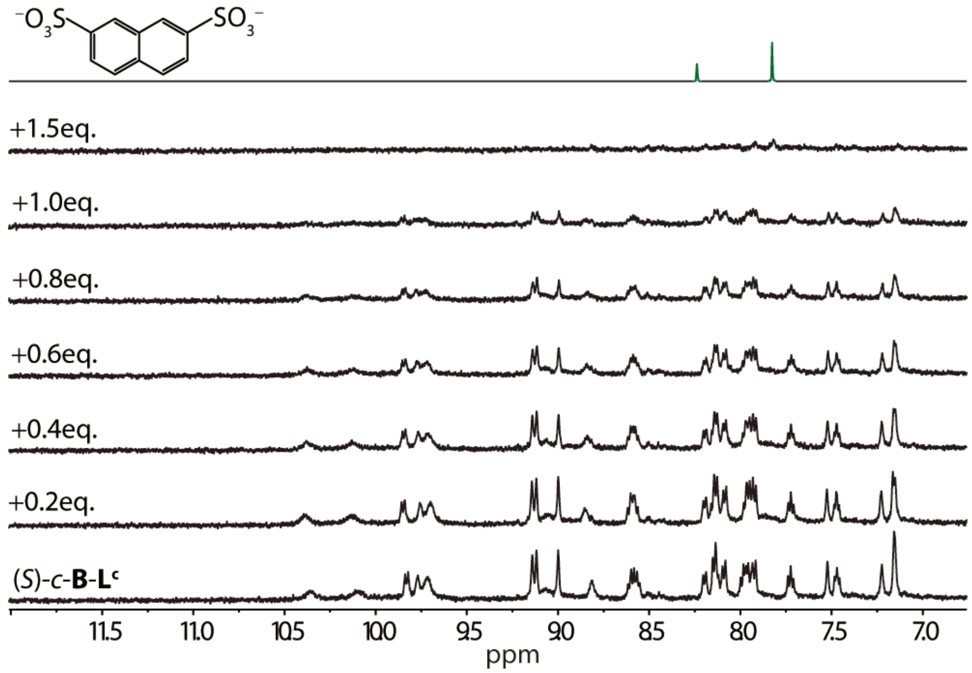

Figure S76. ^{1}H NMR titration (500 MHz, 298 K, acetonitrile-d_3) of (*S*)-*c*-**B**-**L**^{c} with $(NBu_4)_2$**G3**. Similar to **G2**, upon addition of guest **G3**, precipitation occurs immediately, indicating that the guest **G3** cannot be encapsulated.

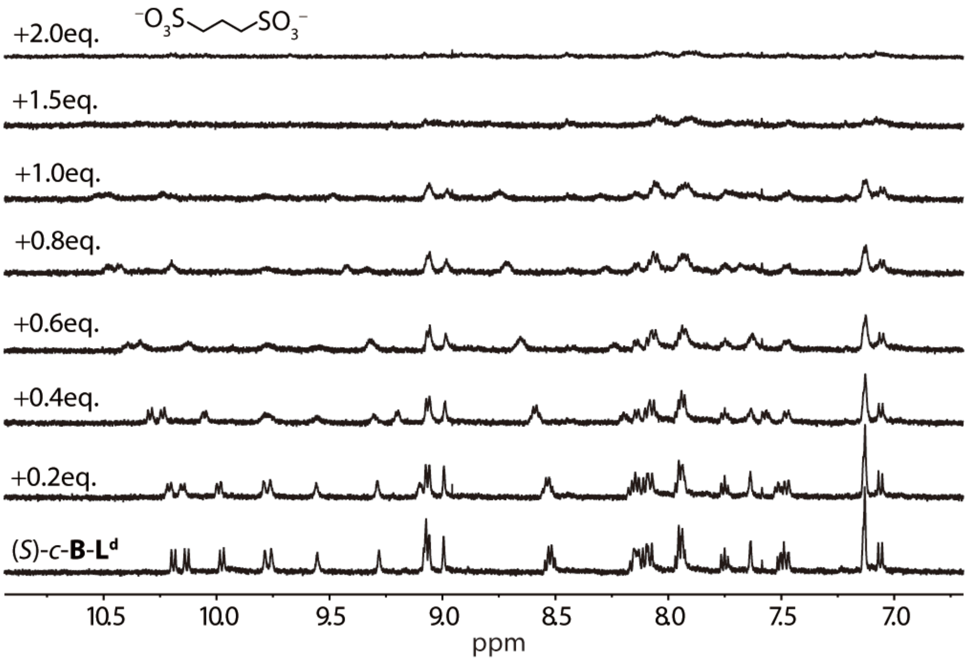

Figure S77. ^{1}H NMR titration (500 MHz, 298 K, acetonitrile-d_3) of (*S*)-*c*-**B**-**L**$^{\mathbf{d}}$ with $(NBu_4)_2$**G1**. Upon addition of guest **G1**, outward-pointing protons H_b and $H_{b'}$ show downfield shifting, indicating that the guest **G1** cannot be encapsulated. Excess addition of **G1** leads to precipitation.

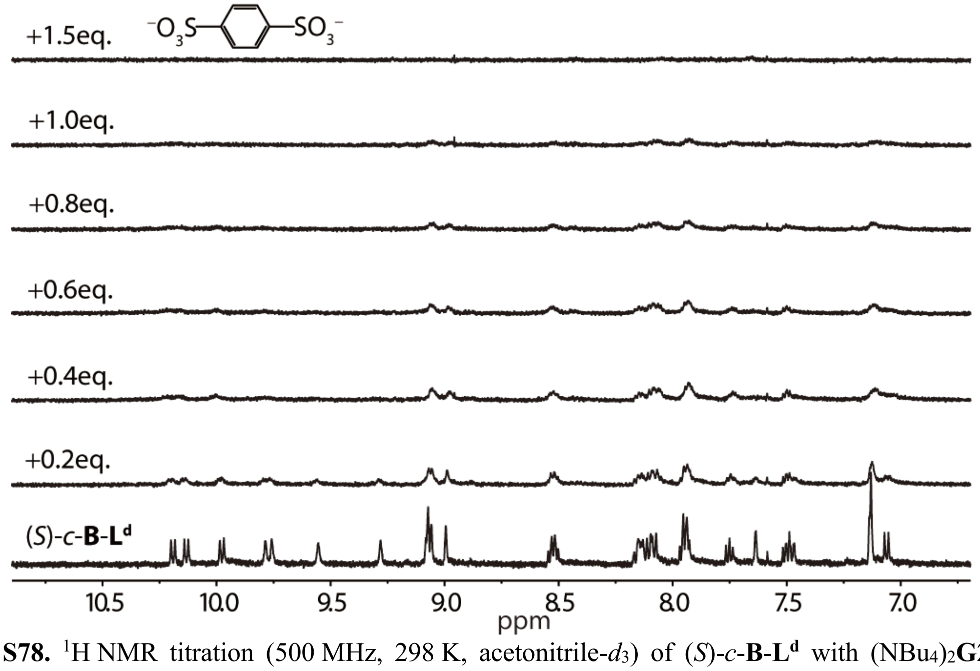

Figure S78. ^{1}H NMR titration (500 MHz, 298 K, acetonitrile-d_3) of (*S*)-*c*-**B**-**L**$^{\mathbf{d}}$ with $(NBu_4)_2$**G2**. Upon addition of guest **G2**, precipitation occurs immediately, indicating that the guest **G2** cannot be encapsulated.

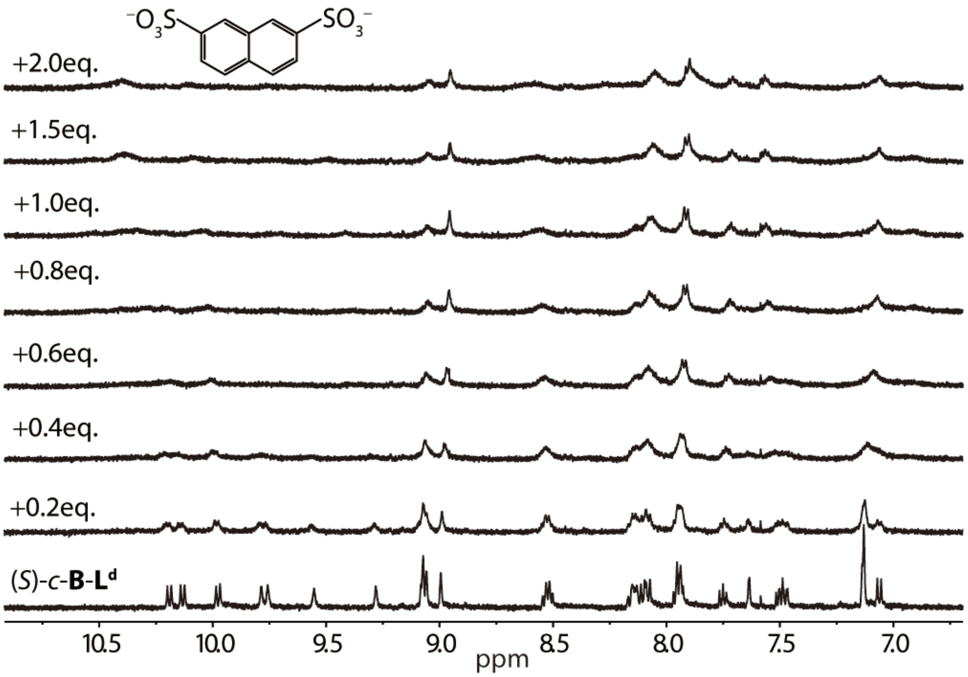

Figure S79. ^{1}H NMR titration (500 MHz, 298 K, acetonitrile-d_3) of (*S*)-*c*-**B**-**L**$^{\mathbf{d}}$ with $(NBu_4)_2$**G3**. Similar to **G2**, upon addition of guest **G3**, precipitation occurs immediately. Even low intensity and broad signals, we can still see only outward-pointing protons H_b and $H_{b'}$ show downfield shifting, indicating that the guest **G3** cannot be encapsulated.

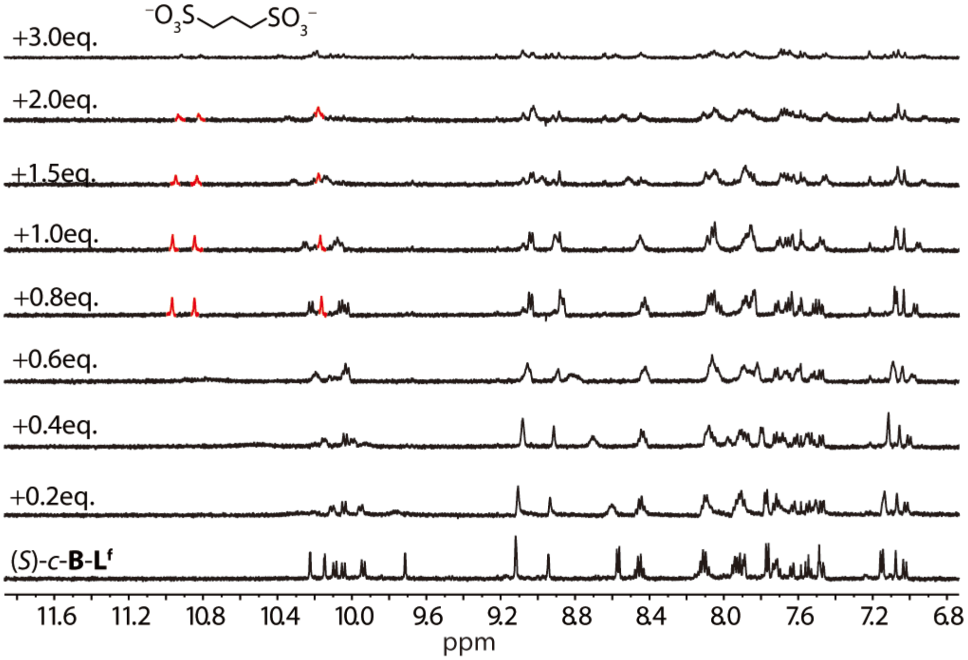

Figure S80. ^{1}H NMR titration (500 MHz, 298 K, acetonitrile-d_3) of (*S*)-*c*-**B**-**L**$^{\mathbf{f}}$ with $(NBu_4)_2$**G1**. Upon addition of one equivalent of guest **G1**, the (*S*)-*c*-**B**-**L**$^{\mathbf{f}}$ cage transforms into [**G1**@(*S*)-*c*-**B**-**L**$^{\mathbf{f}}$]. New set of NMR signals is highlighted in red. Excess addition of **G1** leads to precipitation.

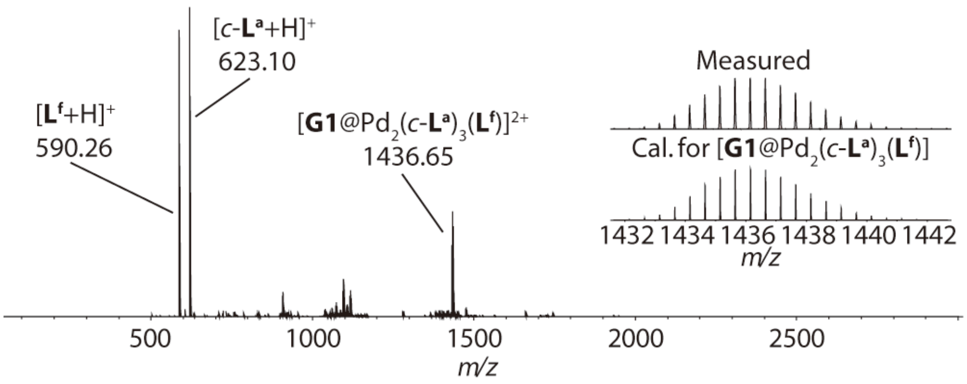

Figure S81. ESI-HRMS spectrum of [**G1**@*c*-**B**-**L**^{f}].

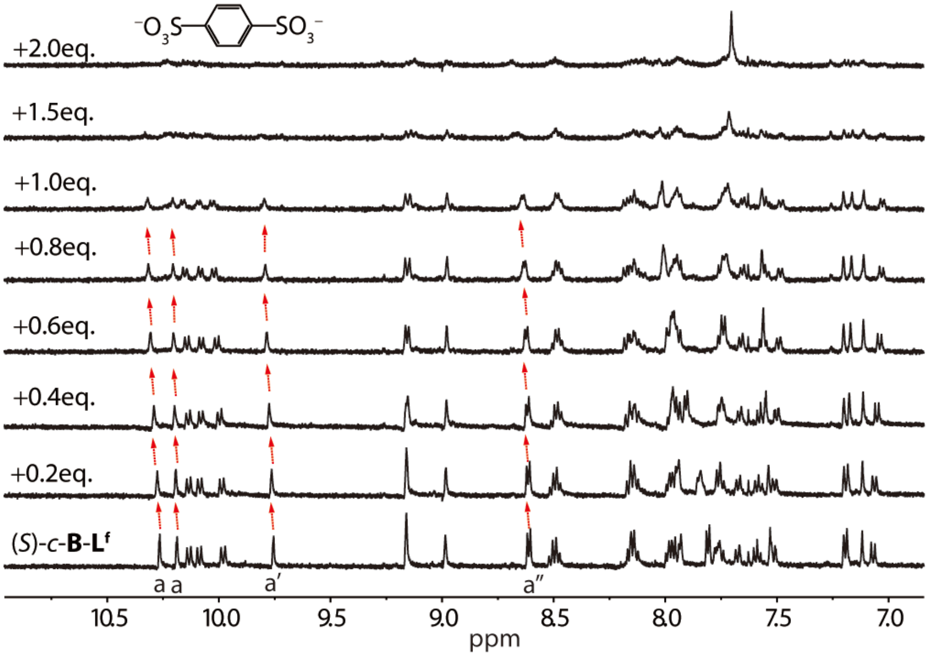

Figure S82. ^{1}H NMR titration (500 MHz, 298 K, acetonitrile-d_3) of (*S*)-*c*-**B**-**L**^{f} with $(NBu_4)_2$**G2**. Upon addition of guest **G2**, the inward-pointing protons H_a and $H_{a'}$ show downfield shifting (highlighted by red arrows), while outward-pointing proton signals do not show any shifting. (*S*)-*c*-**B**-**L**^{f} cage transforms into [**G2**@(*S*)-*c*-**B**-**L**^{f}]. Excess addition of **G2** leads to precipitation.

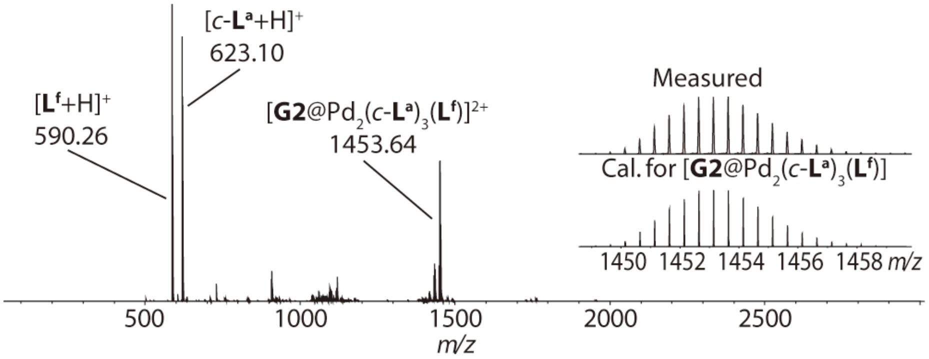

Figure S83. ESI-HRMS spectrum of [**G2**@*c*-**B**-**L**^{f}].

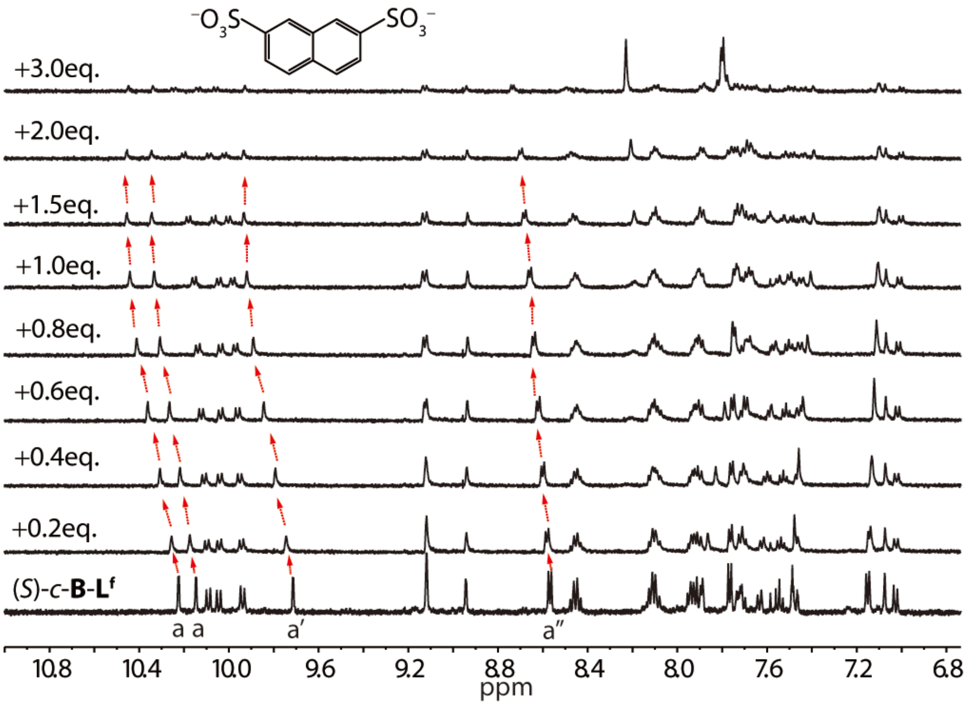

Figure S84. ^{1}H NMR titration (500 MHz, 298 K, acetonitrile-d_3) of (*S*)-*c*-**B**-$\mathbf{L}^\mathbf{f}$ with $(NBu_4)_2$**G3**. Upon addition of guest **G3**, the inward-pointing protons H_a and $H_{a'}$ show downfield shifting (highlighted by red arrows), while outward-pointing proton signals do not show any shifting. (*S*)-*c*-**B**-$\mathbf{L}^\mathbf{f}$ cage transforms into [**G3**@(*S*)-*c*-**B**-$\mathbf{L}^\mathbf{f}$]. Excess addition of **G3** leads to precipitation.

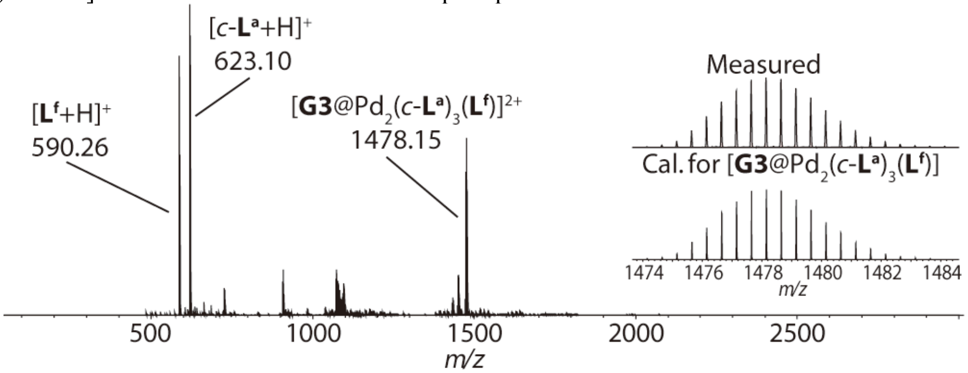

Figure S85. ESI-HRMS spectrum of [**G3**@*c*-**B**-$\mathbf{L}^\mathbf{f}$].

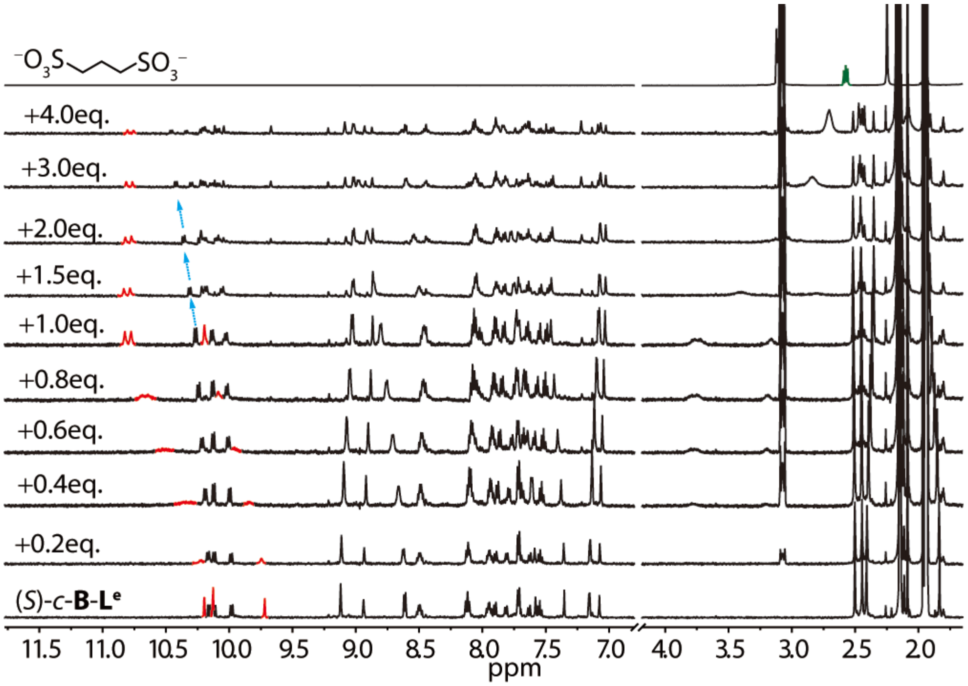

Figure S86. ^{1}H NMR titration (500 MHz, 298 K, acetonitrile-d_3) of (*S*)-*c*-**B**-$\mathbf{L^e}$ with $(NBu_4)_2$**G1**. Upon addition of guest **G1**, the inward-pointing protons H_a and $H_{a'}$ show downfield shifting (highlighted in red), while outward-pointing signals did not show any shifting, indicating the encapsulation of **G1** into (*S*)-*c*-**B**-$\mathbf{L^e}$. Excess addition of **G1** leads to precipitation.

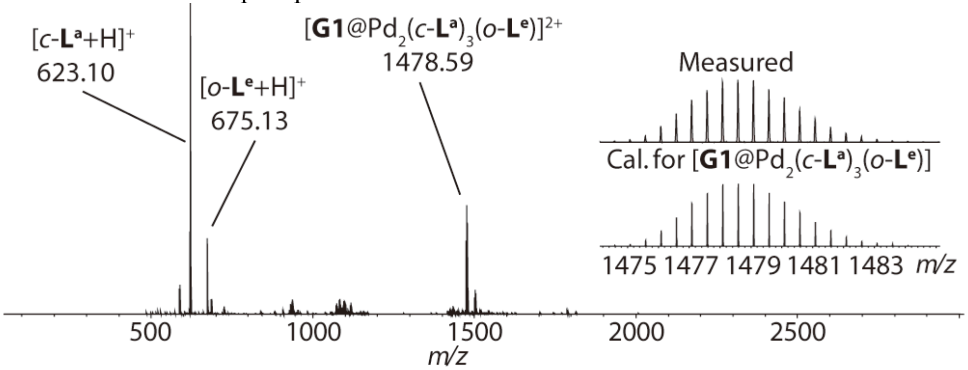

Figure S87. ESI-HRMS spectrum of [**G1**@*c*-**B**-$\mathbf{L^e}$].

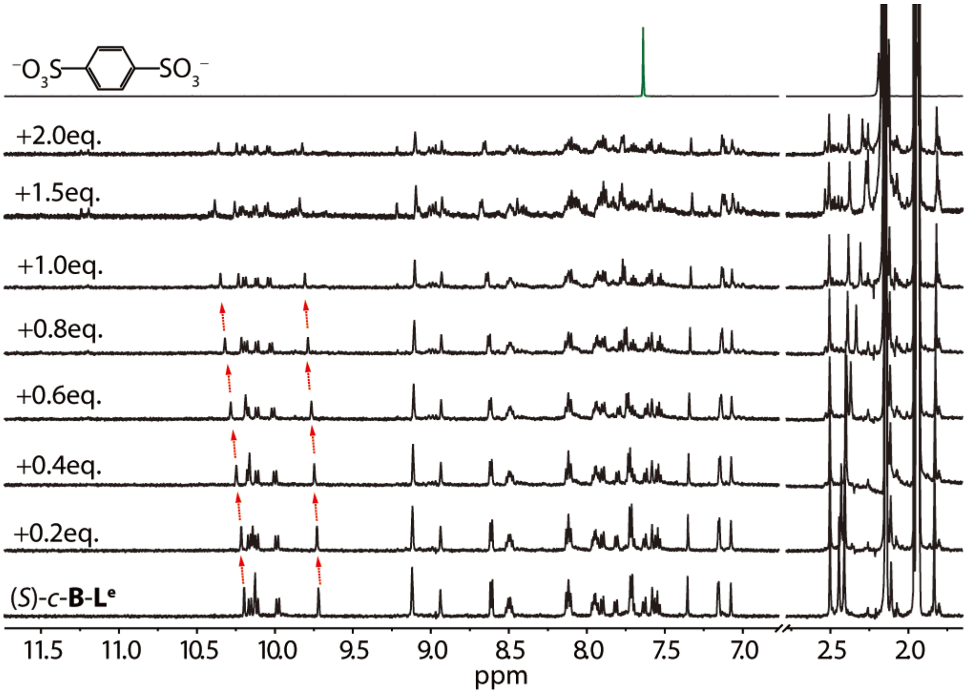

Figure S88. ^{1}H NMR titration (500 MHz, 298 K, acetonitrile-d_3) of (*S*)-*c*-**B**-**L**^{e} with $(NBu_4)_2$**G2**. Upon addition of guest **G2**, the inward-pointing protons H$_a$ and H$_{a'}$ show downfield shifting (highlighted by red arrows), while outward-pointing signals do not show any shifting, indicating the encapsulation of **G2** into (*S*)-*c*-**B**-**L**^{e}. Excess addition of **G2** leads to precipitation.

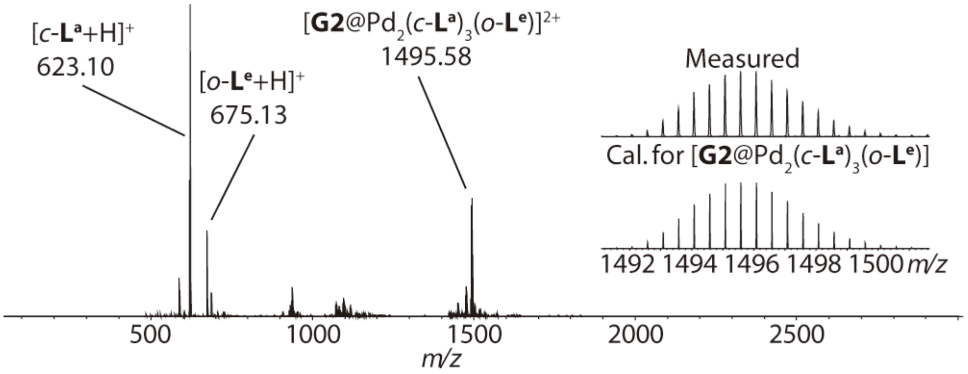

Figure S89. ESI-HRMS spectrum of [**G2**@*c*-**B**-**L**^{e}].

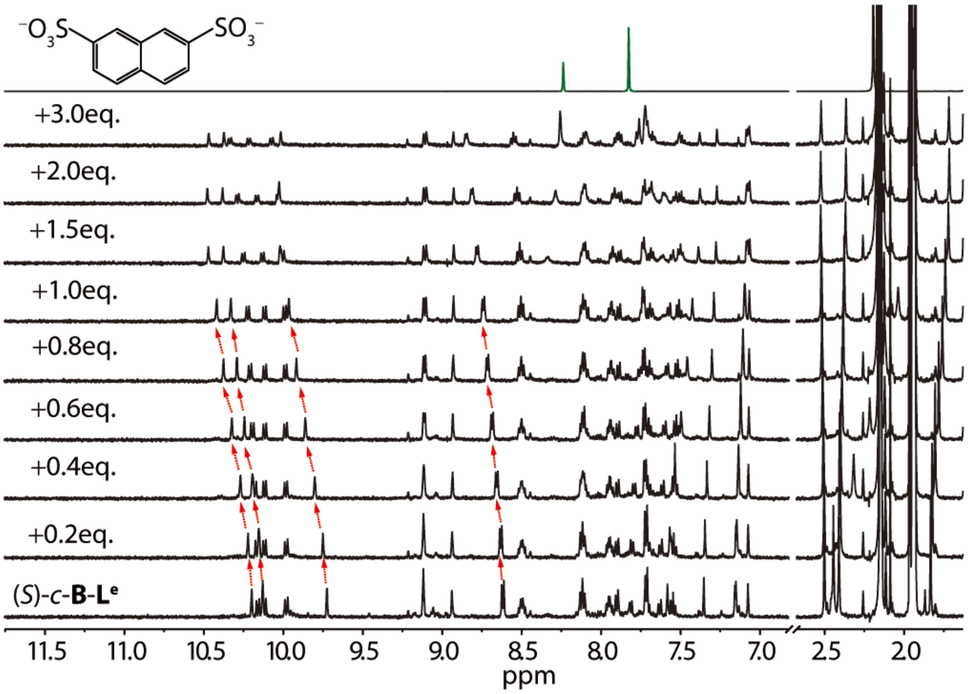

Figure S90. ^{1}H NMR titration (500 MHz, 298 K, acetonitrile-d_3) of (*S*)-*c*-**B**-**L**[e] with $(NBu_4)_2$**G3**. Upon addition of guest **G3**, the inward-pointing protons H_a and $H_{a'}$ show downfield shifting (highlighted by red arrows), while outward-pointing signals do not show any shifting, indicating the encapsulation of **G3** into (*S*)-*c*-**B**-**L**[e]. Excess addition of **G3** leads to precipitation.

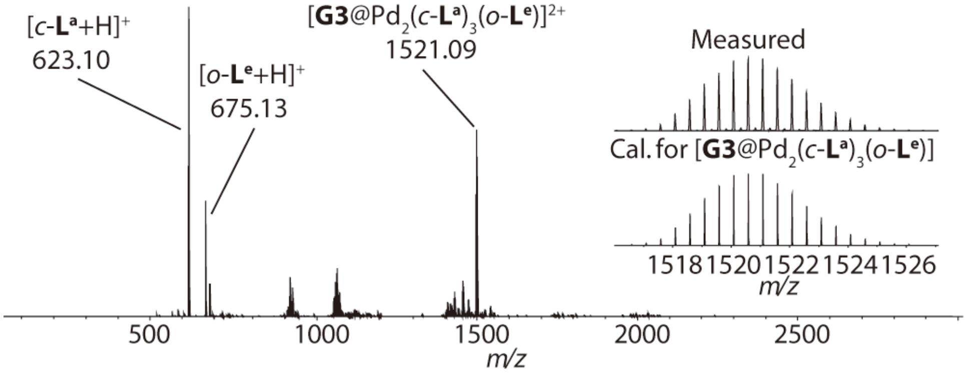

Figure S91. ESI-HRMS spectrum of [**G3**@*c*-**B**-**L**[e]].

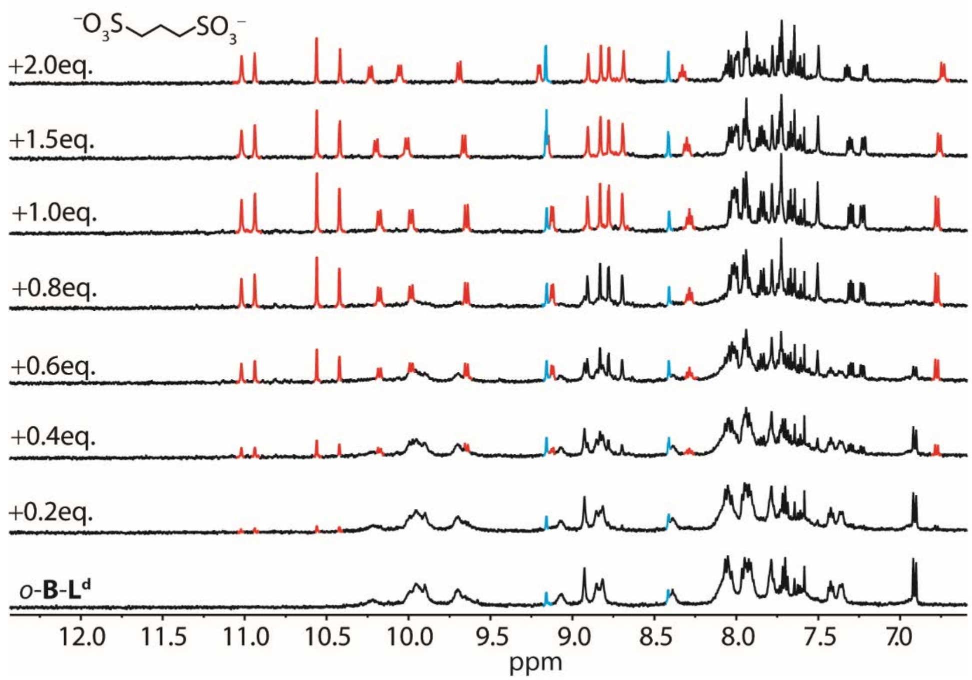

Figure S92. ^{1}H NMR titration (500 MHz, 298 K, acetonitrile-d_3) of *o*-**B**-**L**$^{\mathbf{d}}$ with $(NBu_4)_2$**G1**. Upon addition of one equivalent of guest **G1**, the *o*-**B**-**L**$^{\mathbf{d}}$ cage transforms into [**G1**@*o*-**B**-**L**$^{\mathbf{d}}$]. New set of NMR signals is highlighted in red, the 'free' ligand signals of *o*-**L**$^{\mathbf{a}}$ are marked in blue. Excess addition of **G1** leads to precipitation.

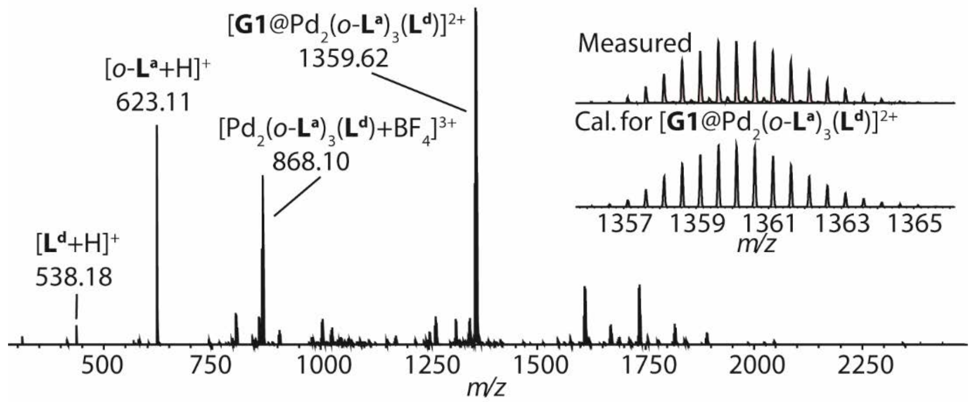

Figure S93. ESI-HRMS spectrum of [**G1**@*o*-**B**-**L**$^{\mathbf{d}}$].

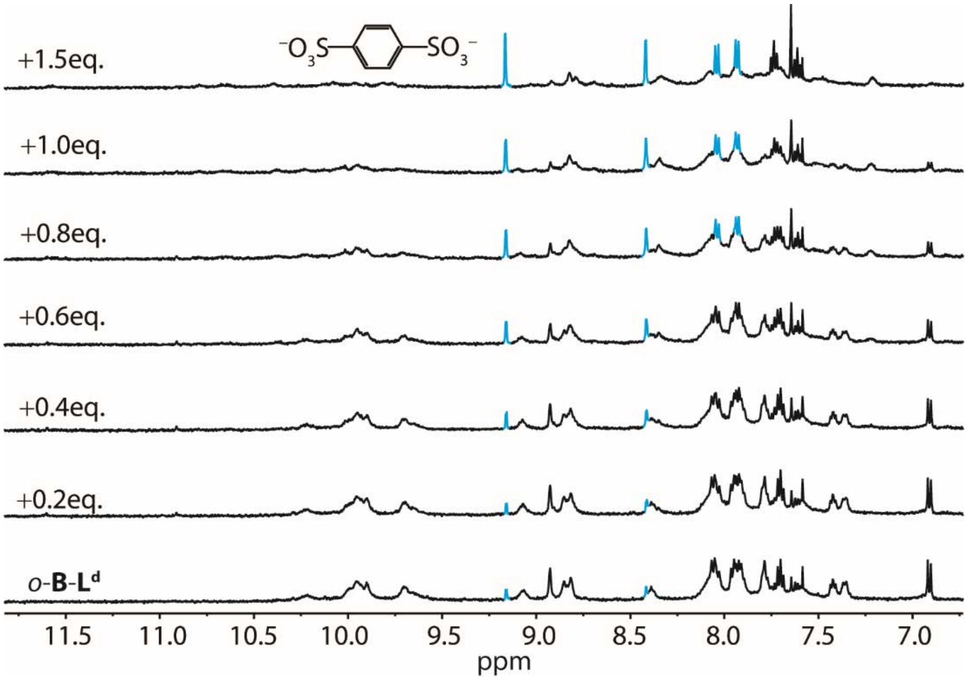

Figure S94. ^{1}H NMR titration (500 MHz, 298 K, acetonitrile-d_3) of *o*-**B**-**L**[d] with $(NBu_4)_2$**G2**. Stepwise addition of guest **G2** lead to the decomposing of *o*-**B**-**L**[d] to 'free' ligand *o*-**L**[a] (marked in blue).

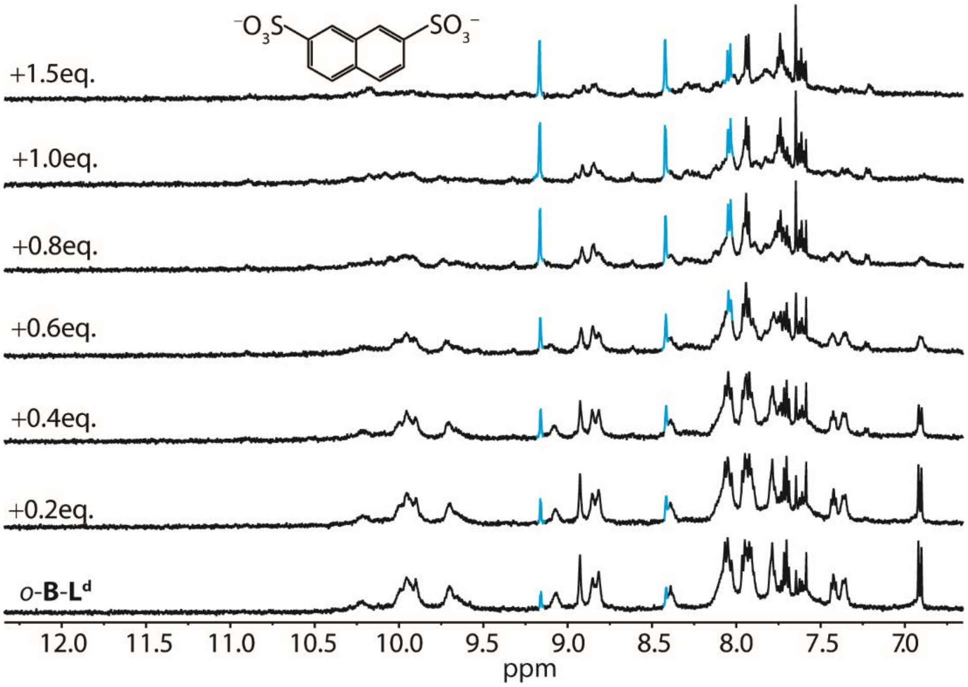

Figure S95. ^{1}H NMR titration (500 MHz, 298 K, acetonitrile-d_3) of *o*-**B**-**L**[d] with $(NBu_4)_2$**G3**. Stepwise addition of guest **G3** lead to the decomposing of *o*-**B**-**L**[d] to 'free' ligand *o*-**L**[a] (marked in blue).

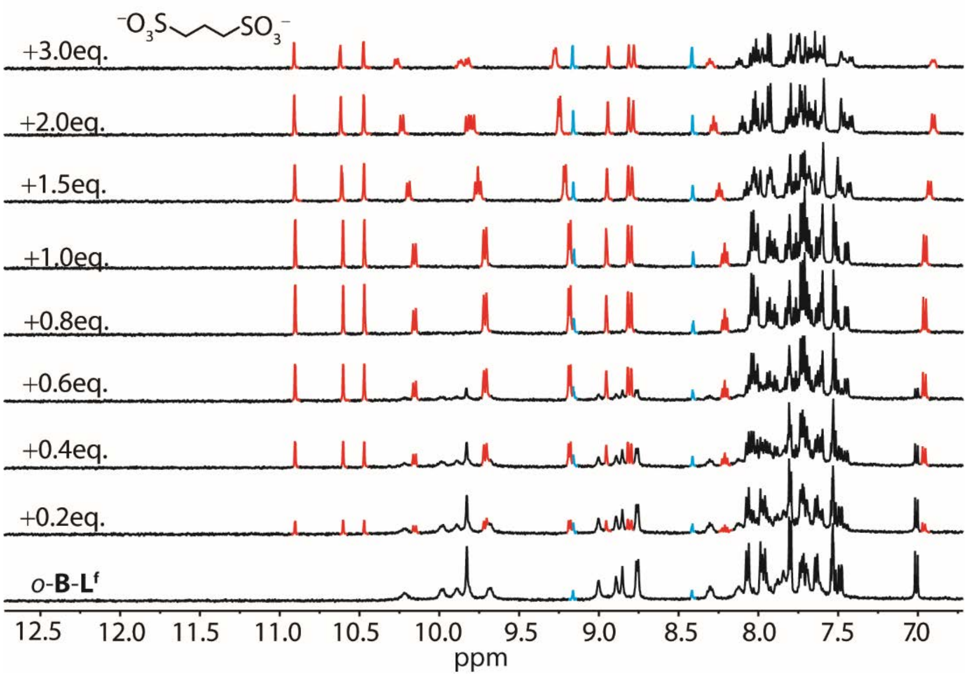

Figure S96. ^{1}H NMR titration (500 MHz, 298 K, acetonitrile-d_3) of *o*-**B**-**L**$^{\mathbf{f}}$ with $(NBu_4)_2$**G1**. Upon addition of one equivalent of guest **G1**, the *o*-**B**-**L**$^{\mathbf{f}}$ cage transforms into [**G1**@*o*-**B**-**L**$^{\mathbf{f}}$]. New set of NMR signals is highlighted in red, the 'free' ligand signals of *o*-**L**$^{\mathbf{a}}$ are marked in blue. Excess addition of **G1** leads to precipitation.

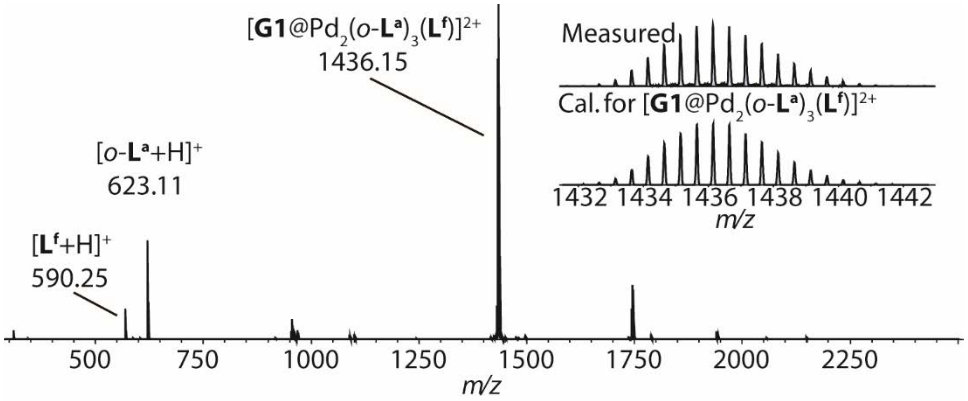

Figure S97. ESI-HRMS spectrum of [**G1**@*o*-**B**-**L**$^{\mathbf{f}}$].

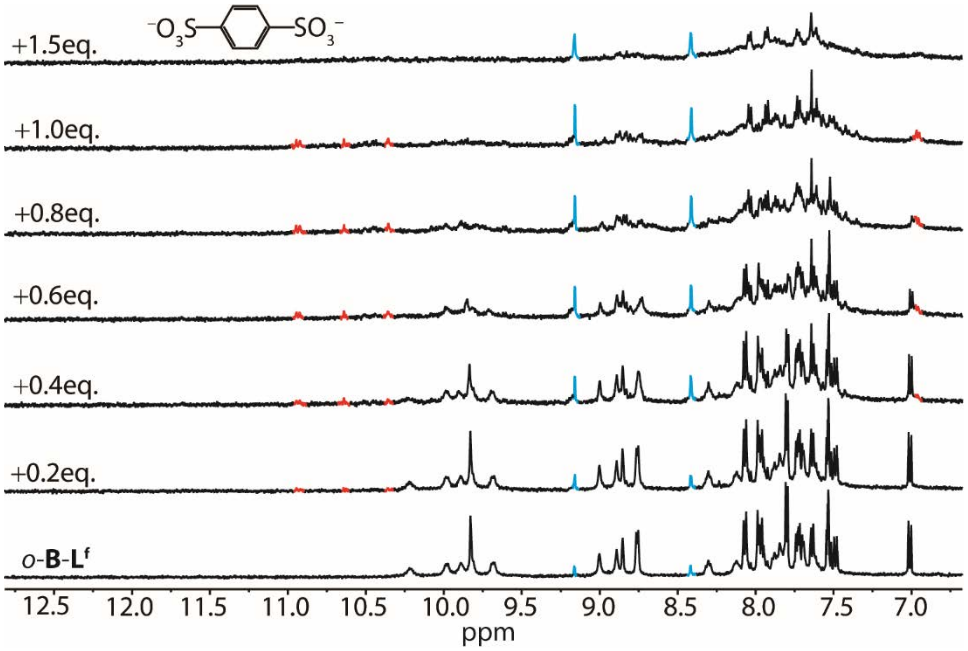

Figure S98. ^{1}H NMR titration (500 MHz, 298 K, acetonitrile-d_3) of *o*-**B**-**L**$^{\mathbf{f}}$ with $(NBu_4)_2$**G2**. Stepwise addition of guest **G2** result in partly decomposing of *o*-**B**-**L**$^{\mathbf{f}}$ to 'free' ligand *o*-**L**$^{\mathbf{a}}$ (marked in blue) and new set of NMR signals belongs to [**G2**@*o*-**B**-**L**$^{\mathbf{f}}$] (highlight in red). Excess addition of **G2** leads to precipitation.

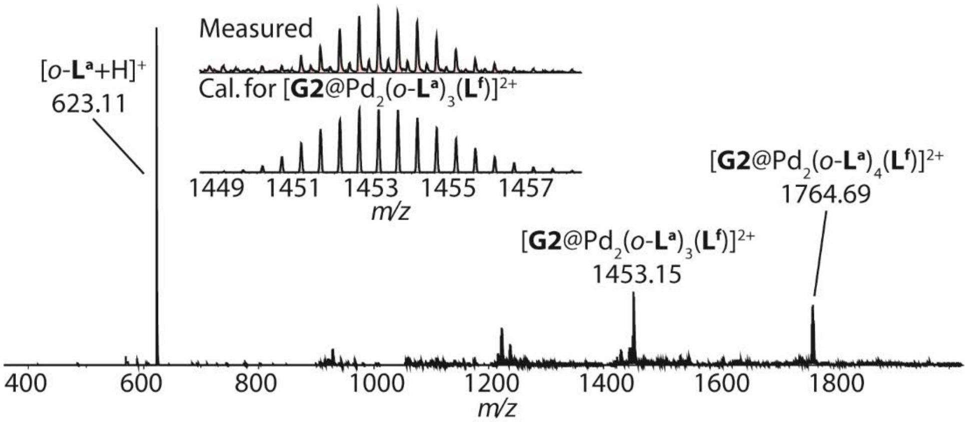

Figure S99. ESI-HRMS spectrum of [**G2**@*o*-**B**-**L**$^{\mathbf{f}}$].

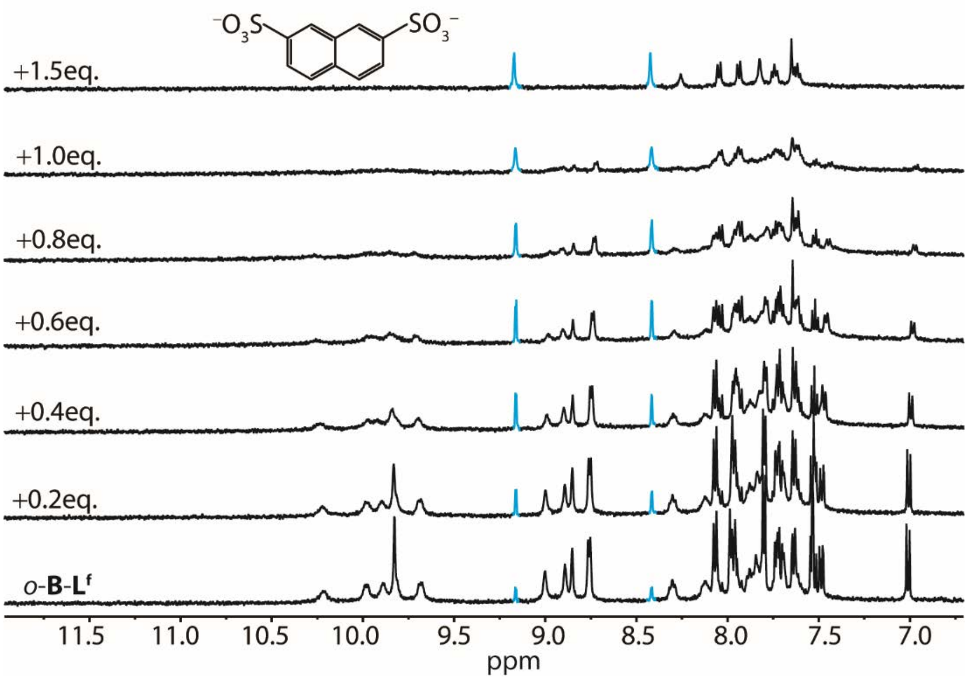

Figure S100. ^{1}H NMR titration (500 MHz, 298 K, acetonitrile-d_3) of *o*-**B**-**L**$^{\mathbf{f}}$ with $(NBu_4)_2$**G3**. Stepwise addition of guest **G3** leads to the decomposing of *o*-**B**-**L**$^{\mathbf{f}}$ to 'free' ligand *o*-**L**$^{\mathbf{a}}$ (marked in blue).

7. Photoswitching

Irradiations at 313 nm were performed by placing a quartz NMR tube in a distance of 5 cm in front of a 300 W Hg arc lamp from LOT-Oriel equipped with a dichroic mirror and 313 nm bandpass filter. The irradiation from *o*-**L**$^{\mathbf{a}}$ (4 mM, ACN) to *c*-**L**$^{\mathbf{a}}$ took around 5 min and from *o*-**C** (1.0 mM, ACN) to *c*-**B** mixture took around 25 min. Completion was checked by NMR spectroscopy. Irradiations at 617 nm were performed by placing a quartz NMR tube 2.5 cm in front of a LED irradiation apparatus (3x 1.4 W 617 nm Power LED, 25 nm FWHM) from Sahlmann Photonics, Kiel. The irradiation from *c*-**L**$^{\mathbf{a}}$ (4 mM, ACN) to *o*-**L**$^{\mathbf{a}}$ took around 5 min and from *c*-**B** (1.0 mM, ACN) to *o*-**B** took around 15 min. Completion was judged from the color (from deep blue to colorless) and checked by NMR spectroscopy.

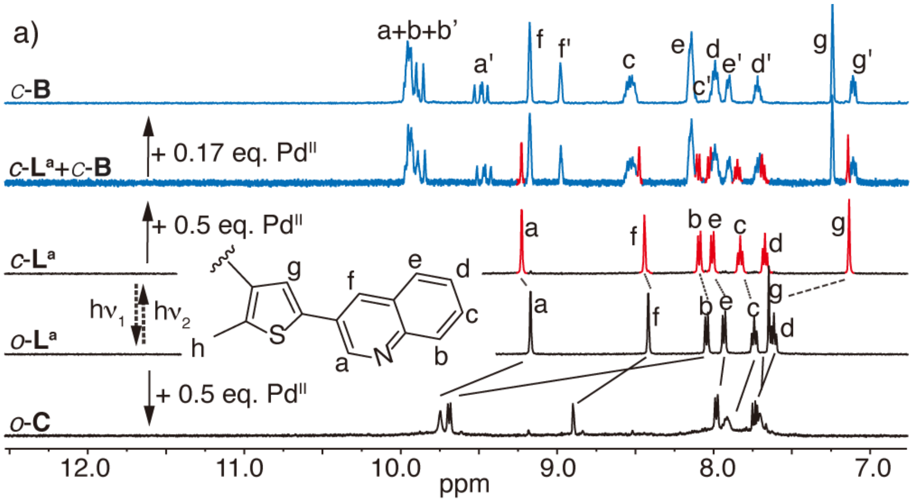

Figure S101. ^{1}H NMR spectra (500 MHz, 298 K, acetonitrile-d_3) of ligands *o*-**L^a**, *c*-**L^a**, open form cage *o*-**C** and closed form bowl *c*-**B** (before and after adjusting stoichiometry by addition of extra palladium).

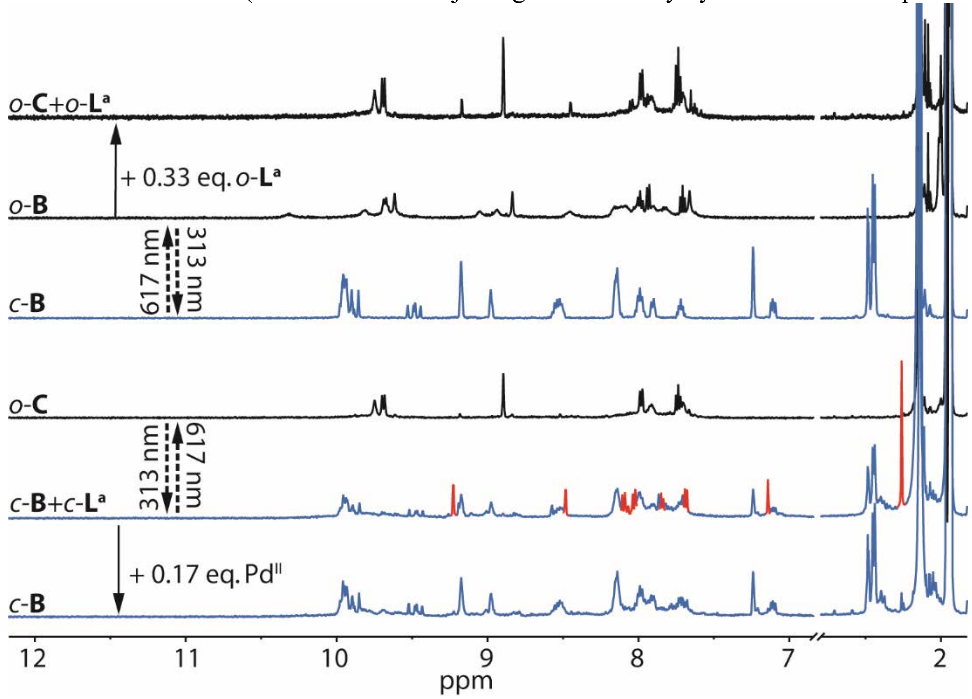

Figure S102. ^{1}H NMR spectra (500 MHz, 298 K, acetonitrile-d_3) of *o*-**C** and *c*-**B**, indicating the changes upon assembly and photoswitching between the cage and bowl (each before and after adjusting stoichiometry by addition of extra Pd(II) or ligand, respectively. Note: both, the *c*-**B** to *o*-**C** as well as the *o*-**C** to *c*-**B** transformations can be achieved either with or without addition of extra ligand or palladium, respectively, to balance the stoichiometry).

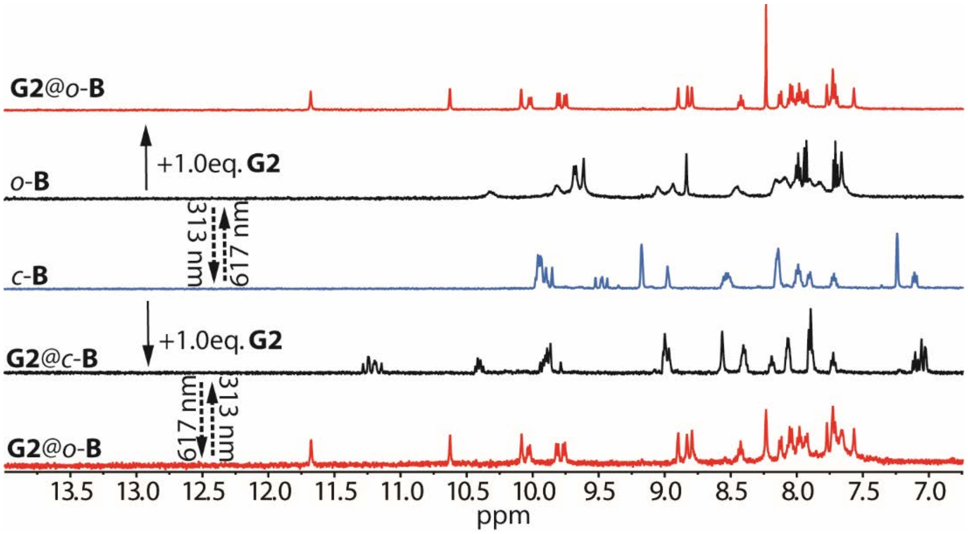

Figure S103. ^{1}H NMR spectra (500 MHz, 298 K, acetonitrile-d_3) of *c*-**B**, *o*-**B**, **G2@*o*-B** and **G2@*c*-B**.

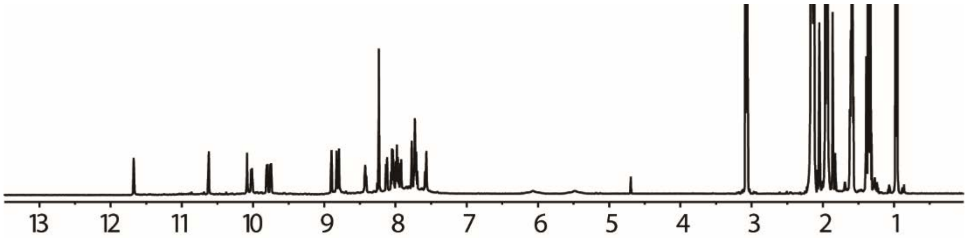

Figure S104. ^{1}H NMR spectra (500 MHz, 298 K, acetonitrile-d_3) of **G2@*o*-B**.

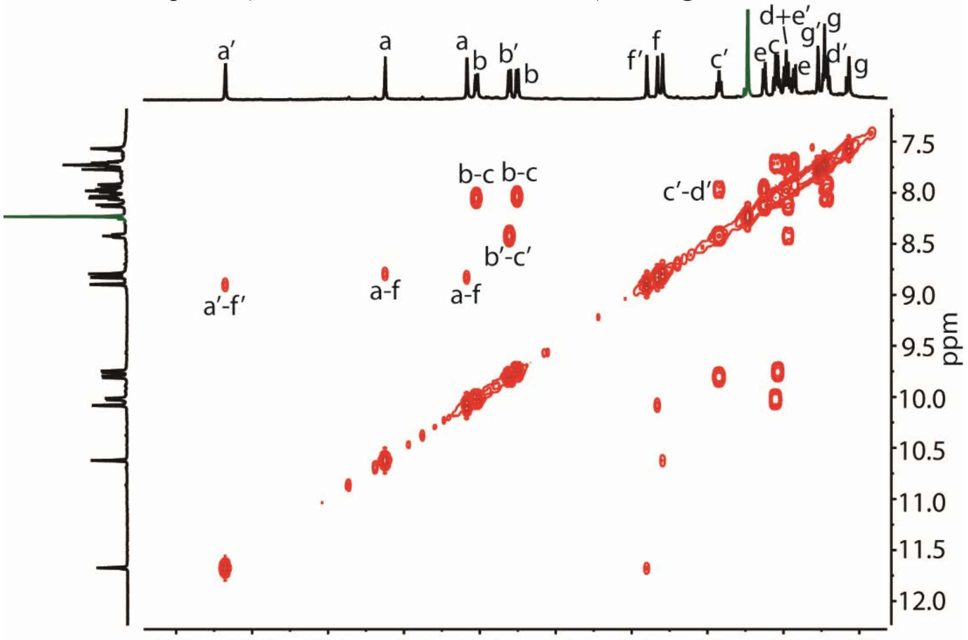

Figure S105. ^{1}H–^{1}H COSY spectrum (500 MHz, 298 K, acetonitrile-d_3) of **G2@*o*-B** (only showing aromatic region).

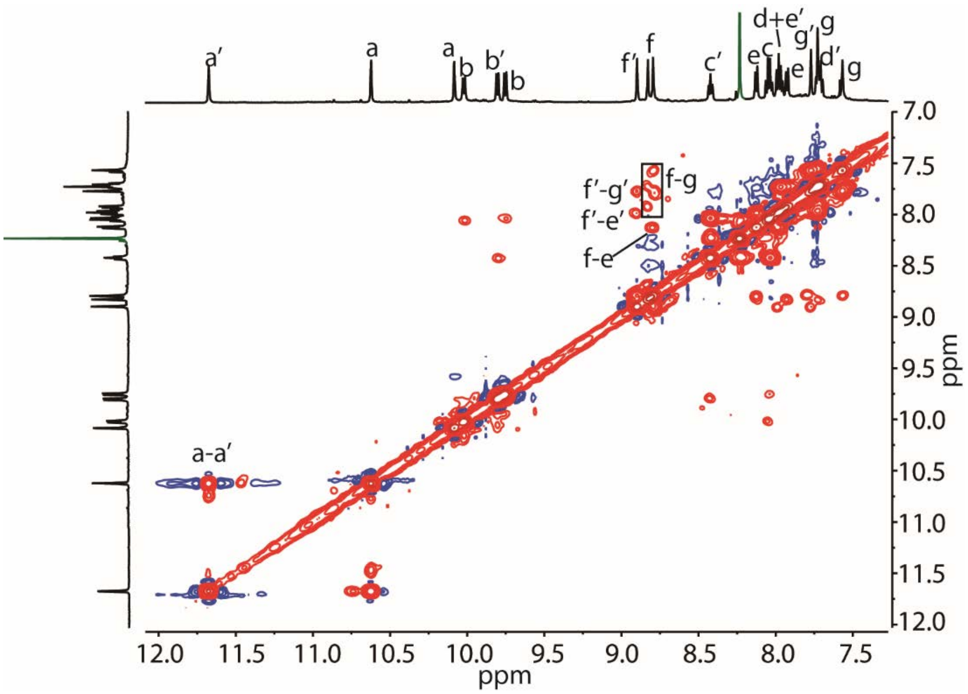

Figure S106. $^{1}H–^{1}H$ NOESY spectrum (500 MHz, 298 K, acetonitrile-d_3) of **G2**@*o*-**B** (only showing aromatic region).

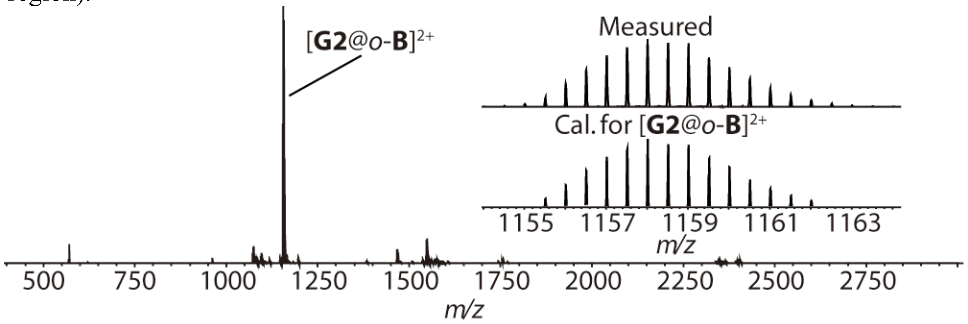

Figure S107. ESI-MS spectrum of **G2**@*o*-**B**.

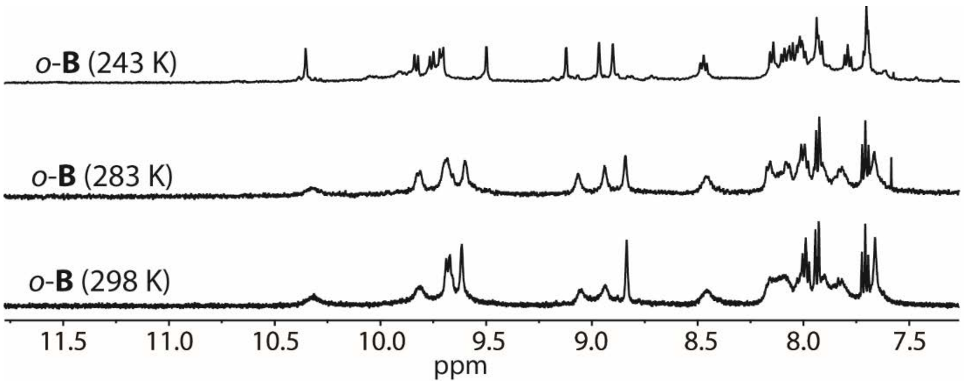

Figure S108. ^{1}H NMR spectra (500 MHz, acetonitrile-d_3) of *o*-**B** at different temperatures.

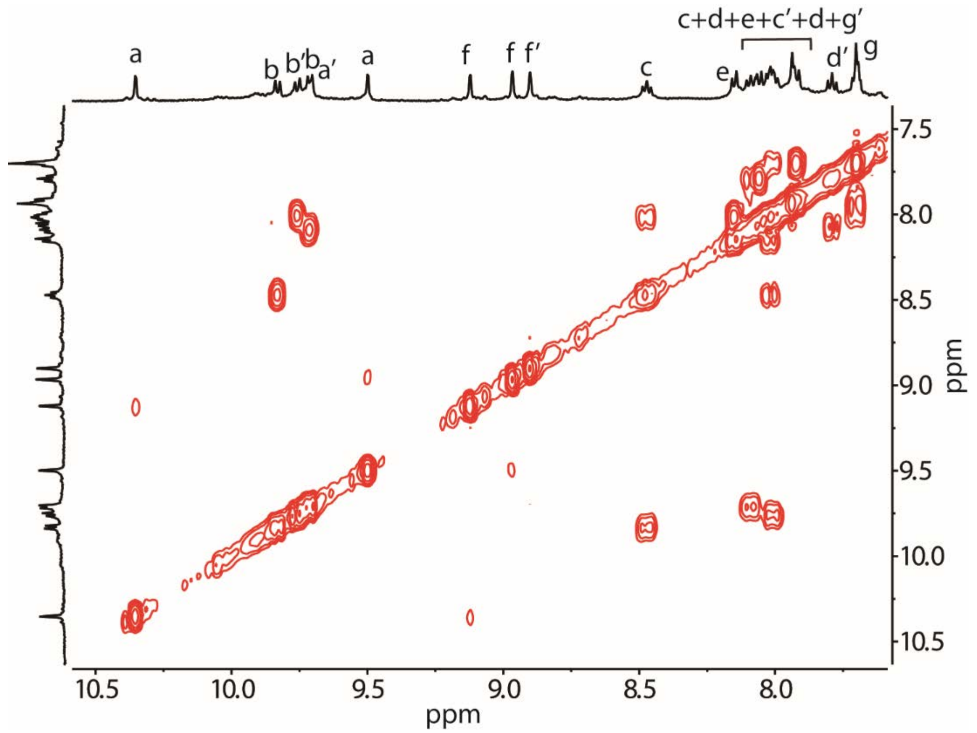

Figure S109. ^{1}H–^{1}H COSY spectrum (500 MHz, 243 K, acetonitrile-d_3) of *o*-**B** (only showing aromatic region).

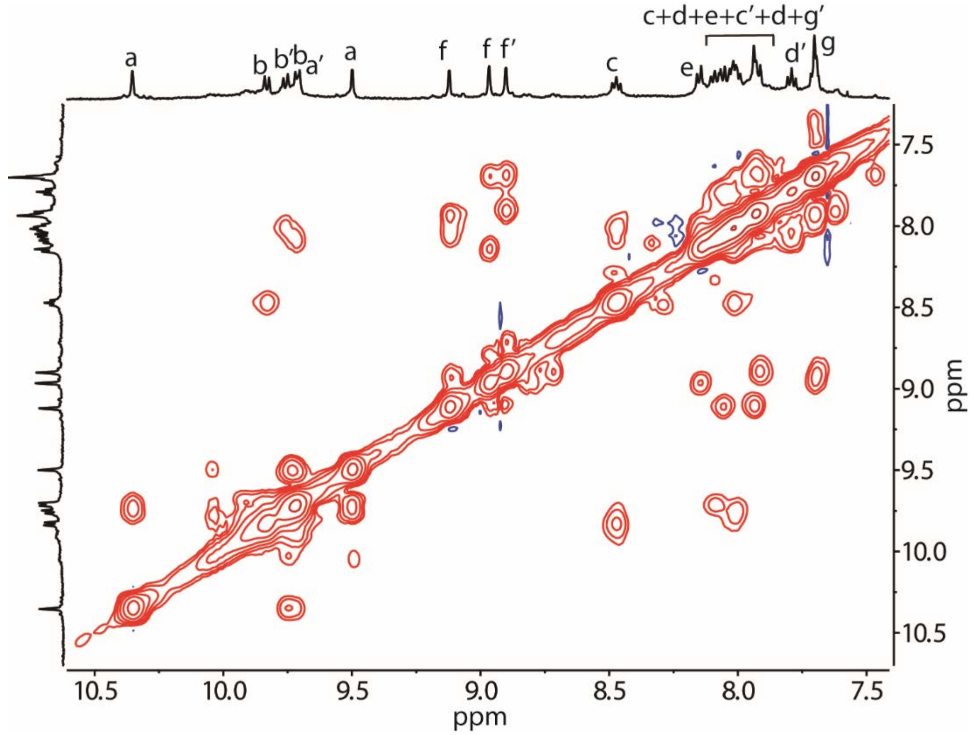

Figure S110. ^{1}H–^{1}H NOESY spectrum (500 MHz, 243 K, acetonitrile-d_3) of *o*-**B** (only showing aromatic region).

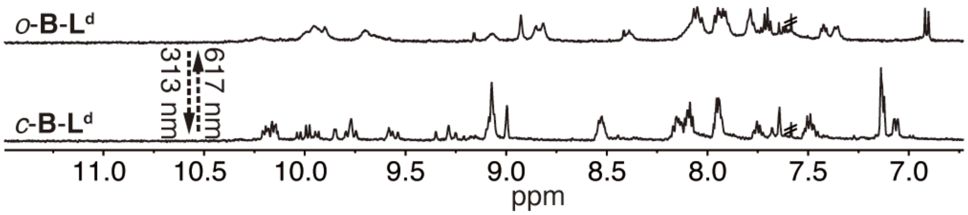

Figure S111. ^{1}H NMR spectra (500 MHz, 298 K, acetonitrile-d_3) showing photoswitching of *c*-**B-L**$^{\mathbf{d}}$ and *o*-**B-L**$^{\mathbf{d}}$ (only showing aromatic region).

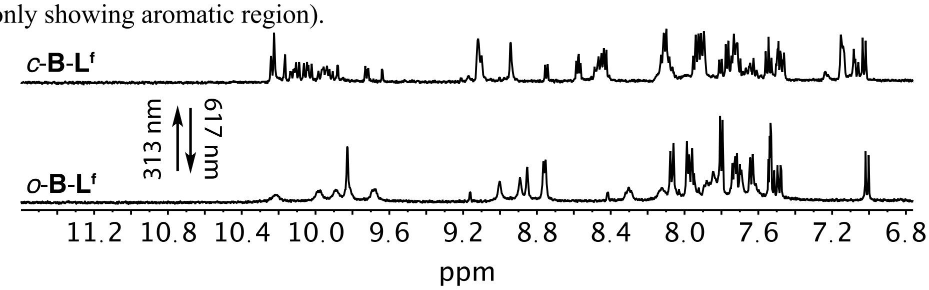

Figure S112. ^{1}H NMR spectra (500 MHz, 298 K, acetonitrile-d_3) showing photoswitching of *c*-**B-L**$^{\mathbf{f}}$ and *o*-**B-L**$^{\mathbf{f}}$ (only showing aromatic region).

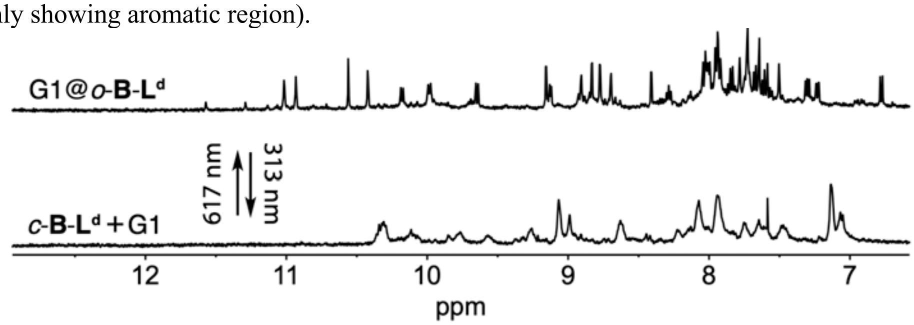

Figure S113. ^{1}H NMR spectra (500 MHz, 298 K, acetonitrile-d_3) showing photoswitching of *c*-**B-L**$^{\mathbf{d}}$+**G1** (**G1** not bound, as compared to titration result in Fig. S77, but there conducted with enantiomerically pure cage (*S*)-*c*-**B-L**$^{\mathbf{d}}$) and G1@*o*-**B-L**$^{\mathbf{d}}$ (only showing aromatic region).

8. Stability of *c*-B

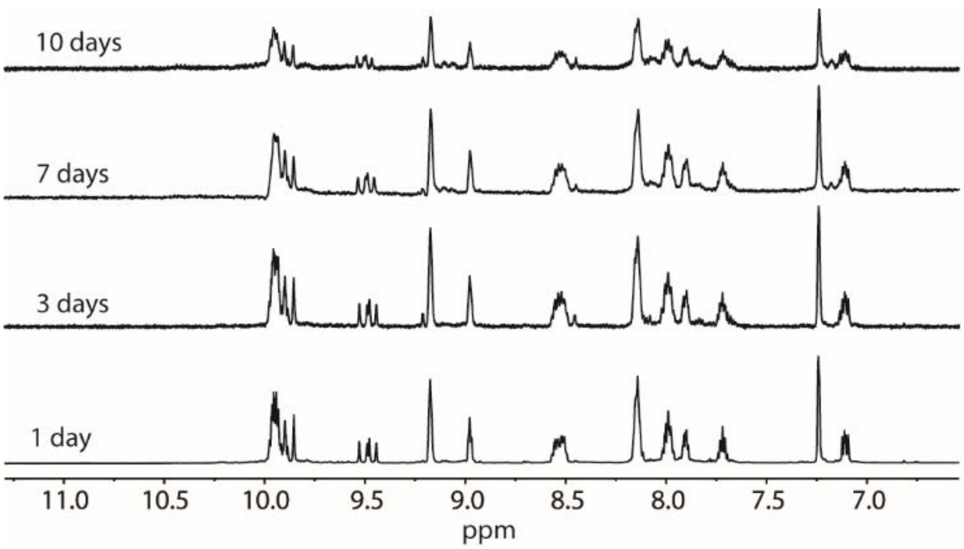

Figure S114. ^{1}H NMR spectra (500 MHz, 298 K, acetonitrile-d_3) of *c*-**B** in 10 days heating under 70 ºC.

9. Structure interconversion

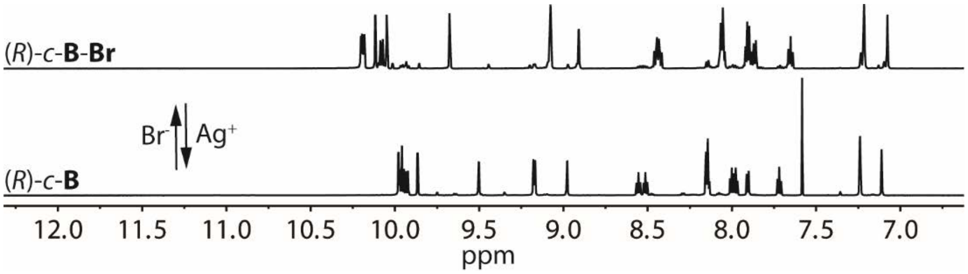

Figure S115. ^{1}H NMR spectra (500 MHz, 298 K, acetonitrile-d_3) of (*R*)-*c*-**B** and (*R*)-*c*-**B-Br**, showing the reversible switching under chemical stimuli of Ag^{+}/Br^{-}.

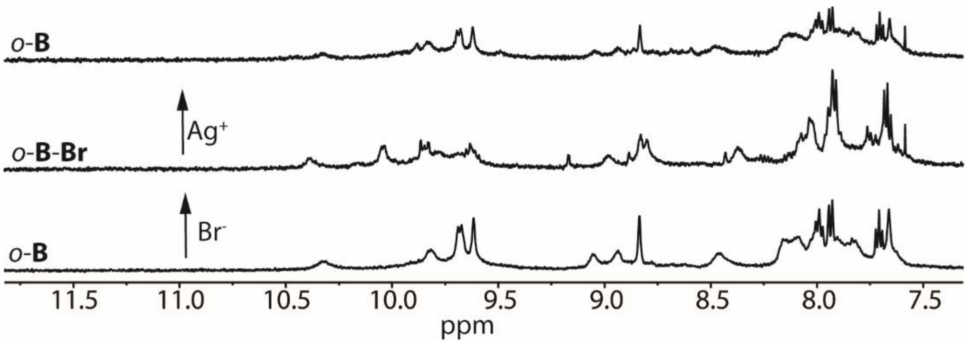

Figure S116. ^{1}H NMR spectra (500 MHz, 298 K, acetonitrile-d_3) of *o*-**B** and *o*-**B-Br**, showing the reversible switching under chemical stimuli of Ag^{+}/Br^{-}.

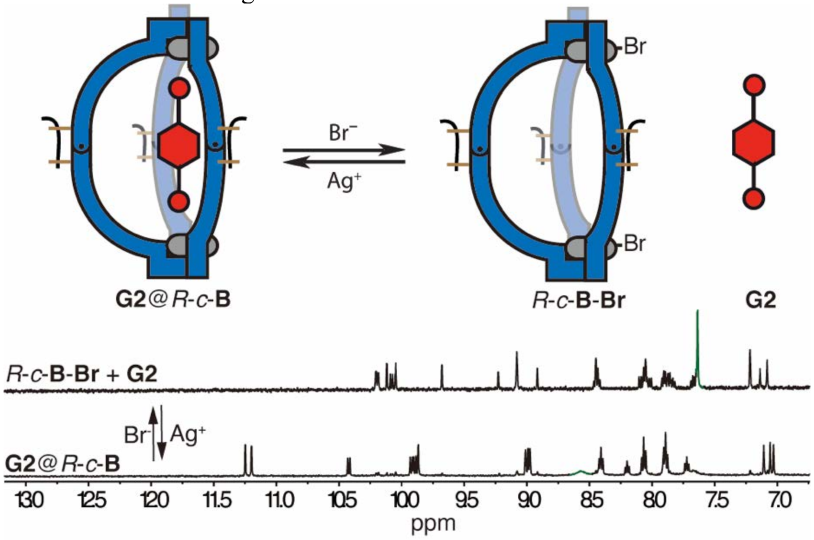

Figure S117. ^{1}H NMR spectra (500 MHz, 298 K, acetonitrile-d_3) of **G2@**(*R*)-*c*-**B** and (*R*)-*c*-**B-Br** with **G2**, showing the reversible capture/release **G2** upon adding Ag^{+}/Br^{-}.

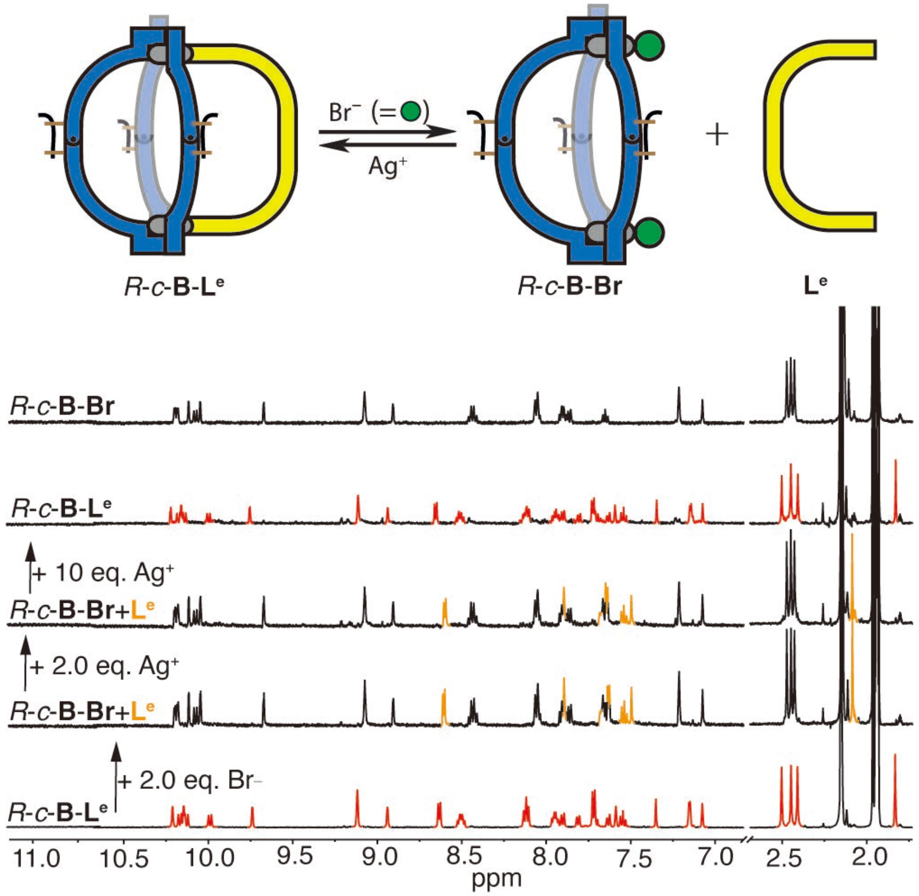

Figure S118. ^{1}H NMR spectra (500 MHz, 298 K, acetonitrile-d_3) of disassembly and re-assembly of heteroleptic cage. The first spectrum on the bottom shows the ^{1}H NMR spectrum of (*R*)-*c*-**B**-**L**^{e}. Stacking up one by one, the following spectrum shows the mixture of releasing **L**^{e} with 2.0 eq. of Br^{-}. After addition of 2.0 eq. or even 10 eq. Ag^{+} into the mixture, the disassembled sample recovered back to (*R*)-*c*-**B**-**L**^{e}. The ^{1}H NMR spectrum on the top setting for reference is (*R*)-*c*-**B**-**Br**. Red color marks the signals of (*R*)-*c*-**B**-**L**^{e}, and green color marks uncoordinated 'free' **L**^{e}.

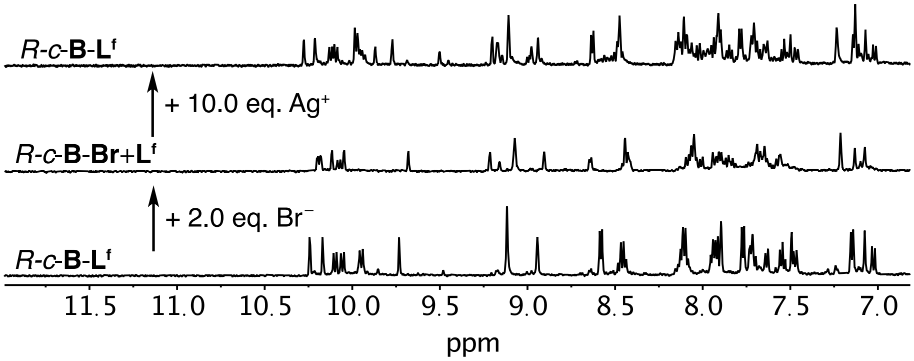

Figure S119. ^{1}H NMR spectra (500 MHz, 298 K, acetonitrile-d_3) of disassembly of heteroleptic cage *R*-*c*-**B**-**L**^{f} into bowl *R*-*c*-**B**-**Br** and reassembly.

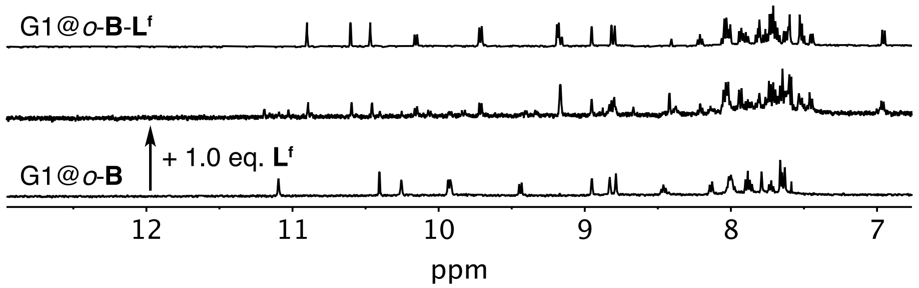

Figure S120. ^{1}H NMR spectra (500 MHz, 298 K, acetonitrile-d_3) of assembly of **G1@*o*-B-L^f** through addition of **L^f** to **G1@*o*-B**. The first spectrum on the bottom shows the ^{1}H NMR spectrum of **G1@*o*-B**, the following the assembly of **G1@*o*-B-L^f** and the top spectrum is **G1@*o*-B-L^f** prepared by guest addition to *o*-**B**-**L^f** as reference.

10. X-Ray data

Crystals of *o*-**C**, *c*-**C** of were obtained by slow vapor diffusion of ethyl ether into 1.0 mM *o*-**C** or *c*-**B** solution in CD_3CN in the dark separately. **G1@*c*-B** and **G1@*o*-B-L^f** crystals were found to grow in the NMR tube after several days in the dark after titrating around 3.0 eq. of **G1** solution (15 mM, CD_3CN) into *c*-**B** (0.5 mM, CD_3CN) or *o*-**B-L^f** (1.0 mM, CD_3CN). Crystals of *o*-**L^e** were obtained after evaporating *o*-**L^e** solution (5 mM, CH_3CN) within several days in the dark.

10.1. Data Collection

Suitable single crystals for X–ray structural analysis of *o*-**C**, *c*-**C** and **G1@*c*-B** were mounted at room temperature in NVH oil. Crystals were stored at cryogenic temperature in dry shippers, in which they were safely transported to macromolecular beamline P11[S7] at the Petra III synchrotron, DESY, Germany. X-ray diffraction data was collected at 80(2) K on a single axis goniometer, equipped with an Oxford Cryostream 800 low temperature device and a Pilatus 6M fast detector. The data integration and reduction were performed with XDS[S8]. The structure was solved by direct methods. The structure model was refined against all data by full-matrix least-squares methods on F^2 with the program SHELXL2014[S9]. The SQUEEZE[S10] method provided by the program Platon[S11] was used to improve the contrast of the electron density map the structure. For **G1@*o*-B**-L^f, the crystal was mounted at ambient temperature in NVH oil and measured on a Bruker D8 Venture diffractometer with an INCOATEC microfocus sealed tube, Iys 3.0 source and Bruker D8 Venture CMOS with Photon 100 detector. Data reduction was performed with SAINT v8.30C (Bruker, 2009a) out of the APEX 3 (Bruker, 2019) program package. SADABS[S12] (version 2014/4) was employed for the incident beam scaling, determination of the spherical harmonic coefficients, outlier rejection and determination of the error model parameters. All the structures were solved by direct methods with SHELXT[S13]. They were refined by full-matrix least-squares against F^2 using SHELXL2014 with the help of the SHELXle[S14] graphical user interface.

Suitable single crystals of *o*-**L^e** were mounted in NVH oil on a nylon loop. X-ray diffraction data were collected on a Bruker d8 venture systems based on a kappa goniometer with Incoatec microfocus X-ray

sources (IµS 2.0), Incoatec QUAZAR mirror optics and a Photon 100 detector. The data were collected at 100 K crystal temperature (Oxford Cryosystems CRYOSTREAM 700), 50 kV and 600 µA and an appropriate 0.5° omega scan strategy. Data reduction was performed with SAINT v8.30C (Bruker, 2009a) out of the APEX II v2.2012.2 0 (Bruker, 2009b) program package. SADABS[S12] (version 2014/4) was employed for the incident beam scaling, determination of the spherical harmonic coefficients, outlier rejection and determination of the error model parameters. All the structures were solved by direct methods with SHELXT[S13]. They were refined by full-matrix least-squares against F^2 using SHELXL2014 with the help of the SHELXle[S14] graphical user interface.

Table S1. Crystallographic data of *o*-**C**, *c*-**C**, **G1**@*c*-**B** and **G1**@*o*-**B**-$\mathbf{L^f}$

	o-**C**	*c*-**C**	**G1**@*c*-**B**	**G1**@*o*-**B**-$\mathbf{L^f}$
CCDC number	2003429	2003430	2003431	2003432
Empirical formula	$C_{140}H_{96}B_3F_{40}N_{10}OPd_2S_8$	$C_{132}H_{80}F_{24}N_8Pd_2S_8$	$C_{109}H_{82}F_{18}N_8O_{14}Pd_2S_{10}$	$C_{153}H_{118}F_{18}N_{13}O_{12}Pd_2S_{11}$
Formula weight	3195.97	2703.32	2603.22	3238.06
Temperature (K)	80(2)	80(2)	80(2)	102(2)
Wavelength (Å)	0.5636	0.6888	0.5636	1.54178
Crystal system	Tetragonal	Tetragonal	Triclinic	Monoclinic
Space group	$P4_2/nbc$	$P4_2/mcm$	P-1	$P2_1/n$
a (Å)	a = 23.508(3)	a = 16.324(2)	a = 14.951(3)	a = 17.4739(8)
b (Å)	b = 23.508(3)	b = 16.324(2	b = 20.918(4)	b = 23.9301(12)
c (Å)	c = 25.231(5)	c = 25.539(5)	c = 22.075(4)	c = 46.079(3)
α (°)	90	90	105.03(3)	90
β (°)	90	90	91.07(3)	95.680(3)
γ (°)	90	90	90.59(3)	90
Volume (Å^3)	13943(5)	6805(2)	6666(2)	19173.3(17)
Z	4	2	2	4
Density (calcd) (Mg/m^3)	1.522	1.31	1.297	1.122
Absorption coefficient (mm^{-1})	0.261	0.425	0.270	3.208
F(000)	6436	2728	2636	6612
Crystal size (mm^3)	0.2 x 0.05 x 0.05	0.150 x 0.150 x 0.150	0.1 x 0.1 x 0.1	0.1 x 0.1 x 0.05
Theta range for data collection (°)	0.971 to 17.638	1.209 to 26.972	0.758 to 19.847	1.846 to 54.848
Index ranges	-25<=h<=25, -25<=k<=25, -27<=l<=27	-21<=h<=21, -21<=k<=21, -33<=l<=33	-18<=h<=18, -25<=k<=25, -26<=l<=26	-18<=h<=18, -25<=k<=25, -48<=l<=48
Reflections collected	118072	91427	132278	151751
Independent reflections	4549 [R(int) = 0.1254]	4375 [R(int) = 0.0606]	22919 [R(int) = 0.1253]	23826 [R(int) = 0.0936]
Completeness	99.90%	100.00%	94.00%	99.50%

Absorption correction	None	None	None	Semi-empirical from equivalents
Refinement method	Full-matrix least-squares on F^2	Full-matrix least-squares on F^2	Full-matrix least-squares on F^2	Full-matrix least-squares on F^2
Data / restraints / parameters	4549 / 822 / 642	4375 / 678 / 288	22919 / 3179 / 1751	23826 / 2642 / 1893
Goodness-of-fit on F^2	1.326	2.383	2.458	1.658
Final R indices [I>2sigma(I)]	R1 = 0.0997, wR2 = 0.2987	R1 = 0.1624, wR2 = 0.4683	R1 = 0.1973, wR2 = 0.5235	R1 = 0.1419, wR2 = 0.3963
R indices (all data)	R1 = 0.1274, wR2 = 0.3270	R1 = 0.1667, wR2 = 0.4766	R1 = 0.2091, wR2 = 0.5290	R1 = 0.1640, wR2 = 0.4178
Extinction coefficient	n/a	n/a	n/a	n/a
Largest diff. peak and hole (e.Å^{-3})	1.700 and -0.980	0.762 and -1.961	4.007 and -1.383	0.520 and -1.740

Table S2. Crystallographic data of *o*-**L**$^{\mathbf{e}}$ (no image of this structure provided here;see CIF file for further data)

	o-**L**$^{\mathbf{e}}$
CCDC number	2003433
Empirical formula	$C_{41}H_{30}F_6N_4S_2$
Formula weight	756.81
Temperature (K)	273(2)
Wavelength (Å)	0.71073
Crystal system	Triclinic
Space group	*P*-1
a (Å)	9.7291(5)
b (Å)	13.2496(7)
c (Å)	15.2727(8)
α (°)	76.829(3)
β (°)	88.449(3)
γ (°)	69.950(3)
Volume (Å^3)	1797.98(17)
Z	2
Density (calcd) (Mg/m^3)	1.398
Absorption coefficient (mm^{-1})	0.216
F(000)	780
Crystal size (mm^3)	0.2 x 0.1 x 0.05
Theta range for data collection (°)	2.232 to 34.971
Index ranges	-15<=h<=15, -21<=k<=21, -24<=l<=24
Reflections collected	296085
Independent reflections	15811 [R(int) = 0.1672]
Completeness	99.90%
Absorption correction	Semi-empirical from equivalents
Refinement method	Full-matrix least-squares on F^2
Data / restraints / parameters	15811 / 791 / 686

Goodness-of-fit on F^2	1.293
Final R indices [I>2sigma(I)]	R1 = 0.0579, wR2 = 0.1820
R indices (all data)	R1 = 0.0969, wR2 = 0.2095
Extinction coefficient	n/a
Largest diff. peak and hole (e.Å^{-3})	0.558 and -0.659

10.2. Refinement Details

For all of the crystal structures, the stereochemical restrains for organic ligands (TO3 for *o*-**L^a^**, TC3 for *c*-$\mathbf{L^a}$, LPT for $\mathbf{L^f}$), solvate molecules (ACN for acetonitrile, ETO for diethyl ether), guests (BUS for **G1**), and counter anions of the structures were generated by the GRADE program using the GRADE Web Server (http://grade.globalphasing.org) and applied in the refinement. A GRADE dictionary for SHELXL contains target values and standard deviations for 1.2-distances (DFIX) and 1.3-distances (DANG), as well as restraints for planar groups (FLAT). The refinement of ADP's for non-hydrogen atoms was enabled by using the new rigid bond restraint (RIGU) in the SHELXL 2014 program in combination with SIMU restraints.

The same atom names are used for different disorder components, which only differ in the part numbers. Therefore, the TABS keyword behind the ACTA instruction (Sheldrick, 2017) was employed to generate the CIF.

For *o*-**C**, *c*-**C**, **G1@*c*-B** and **G1@*o*-B**-L^f, these structures were treated with SQUEEZE routine of the PLATON program, because the crystal lattice contains large voids filled with disordered solvent molecules.

For **G1@*o*-B**-L^f, the structure was refined as a 2-component twin (twin matrix: -1 0 0 0 -1 0 0.5 0 1).

10.3. Structural analysis

The compound *o*-**C** crystalized in the tetragonal space group $P4_2$ while compound *c*-**C** crystallizes in the $P4_2/mcm$ space group and the asymmetric unit contains both possible enantiomers with 50:50 occupancy. The crystal structures further showed that in *o*-**C** the C_2-symmetric ligands are all slightly twisted in the same direction while in *c*-**C**, all *c*-$\mathbf{L^a}$ are arranged in a straight manner without any twisting in with respect to the Pd-Pd axis. For *o*-**C** the angle between the normal to the plane created by Pd and the four coordinating N atoms and the quinoline plane (two different planes due to symmetric reasons), have values of 73.433(3)° and 75.529(3)°, suggesting a twisting over the coordination environment to release the steric pressure. For *c*-**C**, the same angle is 86.845(7)°, much closer to the perpendicular position of a perfectly straight motif. The twisting difference of the quinoline donors around the Pd center is highlighted also by the H···H distance between neighboring quinoline (measured on $H_b \cdots H_b$). In the flexible *o*-**C** the H···H distance span from 2.289 to 2.311 Å for the neighbor atoms, and from 3.237 to 3.269 Å for the opposite one. In the rigid *c*-**C** the hydrogen atoms surrounding the coordination site are in a much closer environment, with distances of 1.718

Å for the neighbor H, and 2.808 Å for the opposite one (Figure S117). Finally, the difference in flexibility is expressed also in the intramolecular Pd···Pd distances, of 12.533(3) Å and 12.256(14) Å for *o*-**C** and *c*-**C**, respectively (Table S3).

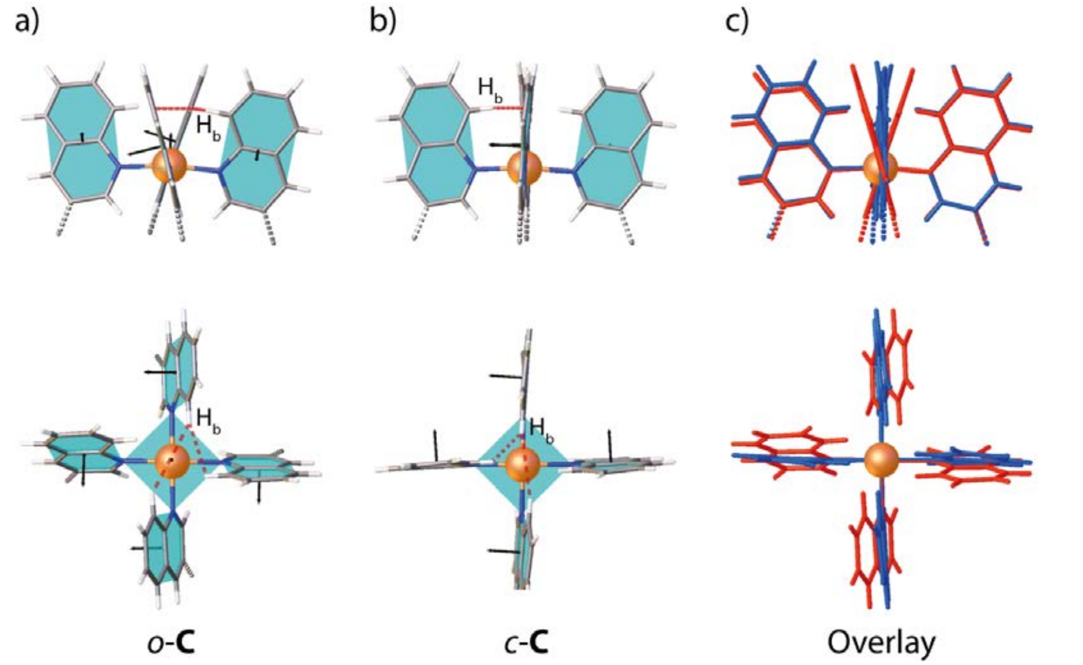

Figure S121. Side and top views of the coordination environment for a) *o*-**C** and b) *c*-**C,** with focus on the H_b···H_b distances (dotted red line) and the twisting between the quinoline planes and the plane created by Pd and the four coordinating N atoms (cyan surfaces); the angle between the planes is measured as the angle between the normal to the planes (black arrow); c) overlay between *o*-**C** (red structure) and *c*-**C** (blue structure) coordination environments.

Table S3. Crystallographic analysis for *o*-**C** and *c*-**C** compared to previously reported *c*-**C**[S15] and *o*-**C**[S1,15].

	o-**C***	*c*-**C**	*o*-**C** [S1]	*o*-**C** [S15]	*c*-**C** [S15]
Pd···Pd distance	12.533(3) Å	12.256(14) Å	16.751(4) Å	15.056(6) Å	14.6707(8) Å
H_b to neighbor H_b distance	2.311 Å and 2.289 Å	1.718 Å	3.2145 (7) and 3.1875 (6) Å ~	From 3.124(1) to 3.998 (1) Å ~	From 2.9192(1) to 3.8073(1) Å ~
H_b to opposite H_b distance	3.269 Å and 3.237 Å	2.808 Å	4.584 (2) and 4.241 (5) Å ~	4.8684(15) and 4.8437(16) Å ~	4.8197(2) and 4.6713(2) Å ~
Twist angle§	73.433(3)° and 75.529(3)°	86.845(7)°	80.84(9)° and 80.7(2)°	From 76.6(3)° to 87.8(3)°	From 79.5(3)° to 88.1(3)°

*there are two values of H···H distances and twist angle due to symmetric reasons.

§calculated as the angle between the normal to the plane created by Pd and the four coordinating N atoms and the normal to the quinoline plane or to the normal to the pyridine plane for the previously reported structures.[S15,S16]

~ pyridine-pyridine outward-pointing protons.

Compound **G1@*c*-B** crystalized in *P-1* space group and the asymmetric unit contains both enantiomers with 50:50 occupancy. Each metal center is coordinated by three quinoline donor groups and one water molecule. **G1** is accommodated in the concave well of the bowl between the two Pd atoms. The following table reports the distances between the oxygen atoms of the sulfonates, pointing inside the bowl cavity, and the neighboring Pd ion and protons H_a of all three ligands. The two disordered parts are reported separately.

Table S4. Crystallographic analysis for **G1@*c*-B**.

	$Pd\cdots O_{water}$	$Pd\cdots O_{SO3}$	Side $H_a\cdots O_{SO3}$	Opposite $H_a\cdots O_{SO3}$
**G1@*c*-B** part1	2.028(10) Å,<br>2.003(11) Å,	3.243(13) Å,<br>3.092(13) Å	2.504(14) Å,<br>2.351(17) Å,<br>2.365(11) Å,<br>2.450(12) Å	2.692(15) Å,<br>2.764(11) Å
**G1@*c*-B** part2	*	3.22(3) Å	2.48(4) Å,<br>2.37(4) Å,<br>2.53(3) Å,<br>2.30(4) Å	2.67(3) Å,<br>2.73(4) Å

* $Pd\cdots O_{water}$ has no disorder

11. UV-vis spectroscopy

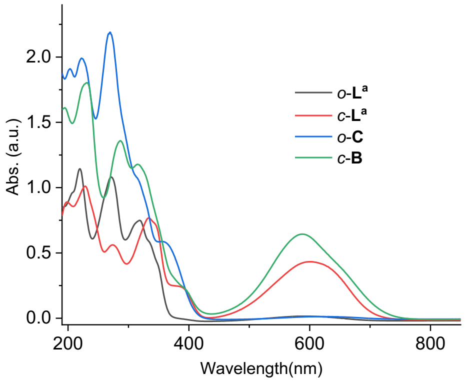

Figure S122. UV-vis spectra of *o*-**L**[a] (0.05 mM), *c*-**L**[a] (0.05 mM), *o*-**C** (0.1 mM) and *c*-**B** (0.1 mM).

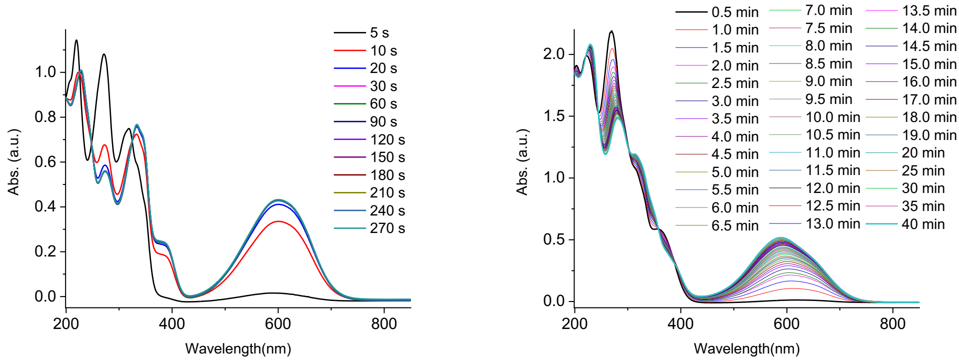

Figure S123. Time-course UV-vis measurements of photocyclization of *o*-**L**[a] (left, 0.05 mM) and *o*-**C** (right, 0.1 mM) under irradiation at 313 nm by placing a quartz cuvette (0.2 mm) in front of a LOT-Oriel 150 W mercury pressure lamp equipped with a dichroic mirror and a 313 nm bandpass filter.

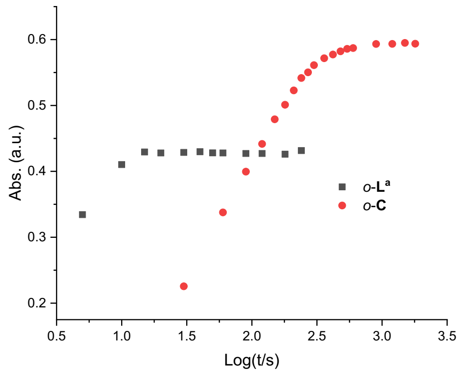

Figure S124. Photocyclization of open-form ligands (*o*-**L**a) and cage (*o*-**C**) under the irradiation at 313 nm over time monitored by UV-vis spectroscopy.

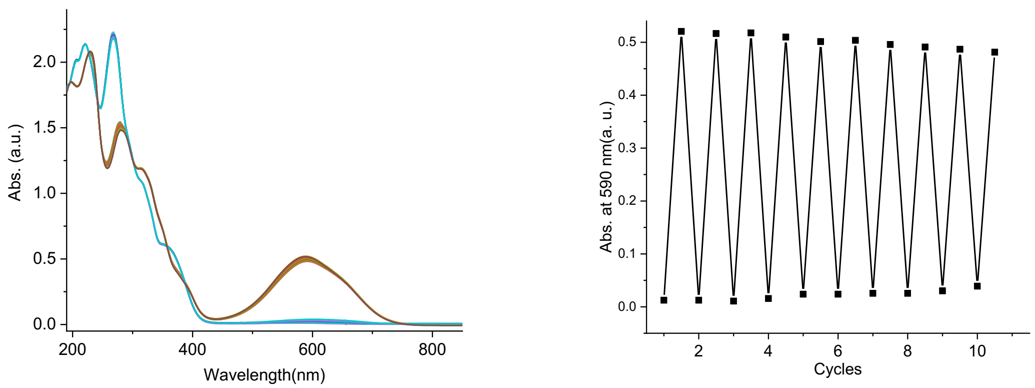

Figure S125. The reversibility of photoswitching between *o*-**C** (0.1 mM) and *c*-**B** was monitored by UV-vis spectroscopy at 590 nm (10 cycles).

12. References

[S1] Han, M.; Michel, R.; He, B.; Chen, Y.; Stalke, D.; John, M.; Clever, G. H. *Angew. Chem. Int. Ed.* **2013**, *52*, 1319.

[S2] Wei, S.; Pan, M.; Fan, Y.; Liu, H.; Zhang, J.; Su, C. *Chem.–Eur. J.* **2015**, *21*, 7418.

[S3] Frank, M. Hey, J. Balcioglu, I. Chen, Y.-S. Stalke, D. Suenobu, T. Fukuzumi, S. Frauendorf, H. Clever, G. *Angew. Chem. Int. Ed.* **2013**, *52*, 10102.

[S4] Löffler, S.; Lübben, J.; Wuttke, A.; Mata, R. A.; John, M.; Dittrich, B.; Clever, G. H. *Chem. Sci.*, **2016**, *7*, 4676.

[S5] Spartan' 08 Version 1.2.0, Wavefunction, Inc., Irvine, CA, **2009**.

[S6] Frisch, M. J.; Trucks, G. W.; Schlegel, H. B.; Scuseria, G. E.; Robb, M. A.; Cheeseman, J. R.; Scalmani, G.; Barone, V.; Mennucci, B.; Petersson, G. A.; Nakatsuji, H.; Caricato, M.; Li, X.; Hratchian, H. P.; Izmaylov, A. F.; Bloino, J.; Zheng, G.; Sonnenberg, J. L.; Hada, M.; Ehara, M.; Toyota, K.; Fukuda, R.; Hasegawa, J.; Ishida, M.; Nakajima, T.; Honda, Y.; Kitao, O.; Nakai, H.; Vreven, T.; Montgomery, J. A., Jr.;

Peralta, J. E.; Ogliaro, F.; Bearpark, M.; Heyd, J. J.; Brothers, E.; Kudin, K. N.; Staroverov, V. N.; Kobayashi, R.; Normand, J.; Raghavachari, K.; Rendell, A.; Burant, J. C.; Iyengar, S. S.; Tomasi, J.; Cossi, M.; Rega, N.; Millam, J. M.; Klene, M.; Knox, J. E.; Cross, J. B.; Bakken, V.; Adamo, C.; Jaramillo, J.; Gomperts, R.; Stratmann, R. E.; Yazyev, O.; Austin, A. J.; Cammi, R.; Pomelli, C.; Ochterski, J. W.; Martin, R. L.; Morokuma, K.; Zakrzewski, V. G.; Voth, G. A.; Salvador, P.; Dannenberg, J. J.; Dapprich, S.; Daniels, A. D.; Farkas, Ö.; Foresman, J. B.; Ortiz, J. V.; Cioslowski, J.; Fox, D. J. Gaussian, Inc., Wallingford CT, **2009**.

[S7] Burkhardt, A.; Pakendorf, T.; Reime, B.; Meyer, J.; Fischer, P.; Stübe, N.; Panneerselvam, S.; Lorbeer, O.; Stachnik, K.; Warmer, M.; Rödig, P.; Göries, D. and Meents, A. *Eur. Phys. J. Plus.*, **2016**, *131*, 56.

[S8] Kabsch, W. *Acta Crystallogr. Sect. D*, **2010**, *66*, 125.

[S9] Sheldrick, G. M. *Acta Crystallogr. Sect. A*, **2008**, *64*, 112.

[S10] Spek,A. *Acta Crystallogr. Sect. C*, **2015**, *71*, 9.

[S11] Spek,A. *Acta Crystallogr. Sect. D*, **2009**, *65*, 148.

[S12] Sheldrick, G. M. *SADABS*, Universität Göttingen, Germany, **2000**.

[S13] Sheldrick, G. M. *Acta Crystallogr. Sect. A*, **2015**, *71*, 3.

[S14] Hubschle, C. B.; Sheldrick, G. M. and Dittrich, B. J. *Appl. Crystallogr.*, **2011**, *44*, 1281.

[S15] Li, R.-J.; Holstein, J. J.; Hiller, W. G.; Andréasson, J.; Clever, G. H. *J. Am. Chem. Soc.* **2019**, *141*, 2097.
